# Role of salt-bridging interactions in recognition of viral RNA by arginine-rich peptides

**DOI:** 10.1016/j.bpj.2021.10.007

**Published:** 2021-10-26

**Authors:** Lev Levintov, Harish Vashisth

**Affiliations:** 1 Department of Chemical Engineering, University of New Hampshire, Durham, New Hampshire

## Abstract

Interactions between RNA molecules and proteins are critical to many cellular processes and are implicated in various diseases. The RNA-peptide complexes are good model systems to probe the recognition mechanism of RNA by proteins. In this work, we report studies on the binding-unbinding process of a helical peptide from a viral RNA element using nonequilibrium molecular dynamics simulations. We explored the existence of various dissociation pathways with distinct free-energy profiles that reveal metastable states and distinct barriers to peptide dissociation. We also report the free-energy differences for each of the four pathways to be 96.47 ± 12.63, 96.1 ± 10.95, 91.83 ± 9.81, and 92 ± 11.32 kcal/mol. Based on the free-energy analysis, we further propose the preferred pathway and the mechanism of peptide dissociation. The preferred pathway is characterized by the formation of sequential hydrogen-bonding and salt-bridging interactions between several key arginine amino acids and the viral RNA nucleotides. Specifically, we identified one arginine amino acid (R8) of the peptide to play a significant role in the recognition mechanism of the peptide by the viral RNA molecule.

## Significance

We reveal key interactions that are involved in the recognition of a viral RNA by a peptide. Specifically, we discovered that the recognition of the peptide depends on the formation of salt bridges and hydrogen bonds that are formed between the arginine residues and the RNA backbone. We also demonstrated that these interactions formed a network of salt bridges that were spanning the major groove of RNA. These results enhance our understanding of the importance of arginine amino acids, or other basic amino acids, in the design of peptides that target viral RNA molecules.

## Introduction

Numerous functions of RNA molecules depend on their interactions with proteins (1), which play a crucial role in various phases of the cell life cycle, including gene regulation (2,3), transcription (4,5), and translation (6). Consequently, misregulation of RNA-protein interactions can lead to neurological disorders, cardiovascular problems, and oncogenic diseases (7, 8, 9, 10). Moreover, the interactions between viral RNA molecules and cellular or viral proteins are involved in the replication and transcription processes of various viruses, for example, human immunodeficiency virus (HIV), hepatitis C virus, and severe acute respiratory syndrome coronavirus (SARS CoV/CoV2) (11, 12, 13, 14). Therefore, resolving the mechanistic details of RNA-protein interactions is essential for understanding various biological and biophysical processes (2, 3, 4, 5, 6, 7, 8, 9, 10, 11, 12, 13, 14).

Proteins and short peptides often interact with RNA molecules by adopting an *α*-helical or a *β*-sheet structure that can fit into the binding pocket of an RNA molecule (15, 16, 17, 18, 19, 20, 21) or through the interactions with the RNA backbone (1,22,23). Specifically, the RNA-peptide complexes are considered good model systems to study RNA-protein interactions and to probe the recognition mechanisms (24,25). A general RNA binding protein domain is the arginine-rich motif (ARM), which is found in ribosomal proteins (26), ribonucleoproteins (1,27), and viral proteins (11,28). The ARMs are short peptides that have a high concentration of arginine residues and have high affinity and specificity of interaction with their targets by adopting various conformations including *α*-helical, *β*-hairpin, or extended conformations (29). The interactions between these ARMs and RNA molecules have been investigated using NMR spectroscopy (11,15,30, 31, 32, 33, 34), circular dichroism spectroscopy (35,36), x-ray crystallography (37,38), and combinations of experimental and computational methods (29,39, 40, 41). Several comprehensive investigations have been conducted on the nucleic-acid-protein interfaces using structural and shape analyses to establish common features across known complexes (42, 43, 44, 45). Overall, these studies showed that the RNA-protein interactions are governed by sequence (e.g., composition of amino acids and nucleotides) or by shape (e.g., recognition of specific shapes of proteins).

However, the role of dynamics in RNA-protein interactions is still not fully understood because of challenges in capturing all the required parameters for describing a complex biomolecular system (23,46,47). Computational methods such as molecular dynamics (MD) simulations that are rooted in biophysical modeling are promising tools to enhance our knowledge of the recognition mechanism between RNA molecules and proteins by characterizing molecular motions at the atomic level (48). Although several RNA-protein complexes, for example, RNA-U1A complex (49, 50, 51, 52, 53) and other RNA recognition motifs (40,54, 55, 56, 57, 58, 59, 60, 61), various double-stranded RNA-protein complexes (39,62, 63, 64, 65, 66, 67, 68), ribosomal RNA-protein complexes (69, 70, 71, 72), and transfer RNA-protein complexes (73, 74, 75, 76, 77, 78, 79), have been investigated using MD simulations and free-energy methods, only a few studies have been conducted to investigate the interactions in viral RNA-protein complexes (80, 81, 82, 83, 84, 85, 86). Specifically, the studies on the viral RNA-protein complexes highlighted the importance of electrostatic interactions and the interactions between water molecules and proteins. However, most of these studies (80, 81, 82, 83, 84) were reported over a decade ago, and the force fields for nucleic acids and proteins have significantly improved in recent years (87). Additionally, the timescales of conventional MD simulations performed in these studies were limited. Thus, we still lack a full understanding of the viral RNA-protein recognition mechanisms and of specific interactions that need to be created or disrupted during the binding-unbinding process.

**Figure 1:**
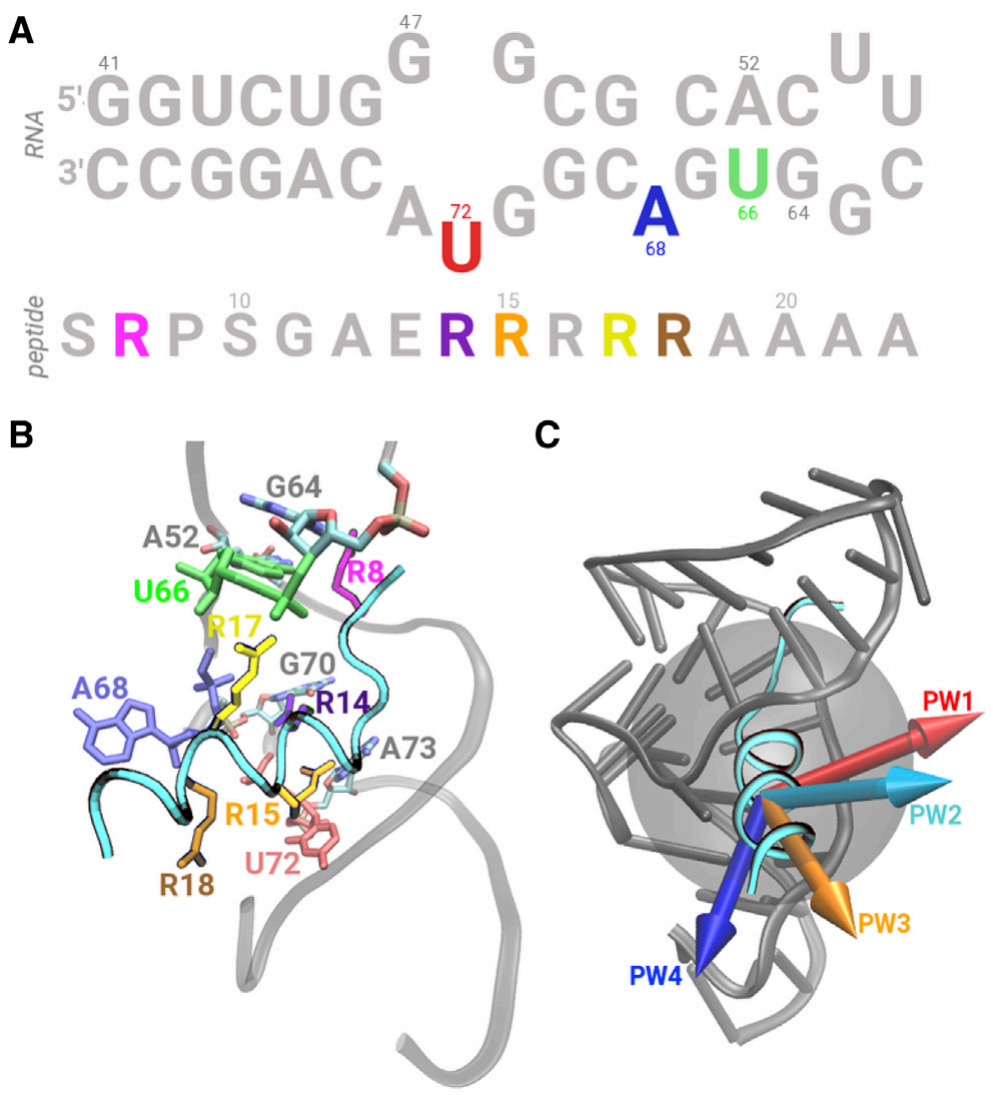
Structural details and system setup. (*A*) The sequences of the HIV-1 RRE RNA and the RSG-1.2 peptide are shown. The key nucleotides and amino acids are highlighted in unique colors. (*B*) A side-view of the binding pocket is shown in which the peptide is rendered as a cyan tube with the side chains of key residues highlighted in stick representations. Each key nucleotide in the RNA and each key amino acid in the peptide are highlighted in a unique color and labeled. (*C*) A side-view of the RRE RNA (*gray cartoon*) and the peptide (*cyan cartoon*) complex is shown. A transparent gray sphere represents the approximate volume of the peptide binding pocket. Each arrow corresponds to the peptide dissociation coordinate or direction for one of the four pathways (PWs): PW1 (*red*), PW2 (*cyan*), PW3 (*orange*), and PW4 (*blue*). To see this figure in color, go online.

To address these questions, we applied nonequilibrium constant velocity steered MD (cv-SMD) simulations to study the binding-unbinding process of a helical arginine-rich peptide (RSG-1.2) from a conserved HIV-1 Rev response element (RRE) RNA segment, which is located in the *env* coding region and plays an essential role in viral replication (Fig. 1 *A*) (88). The RSG-1.2 peptide is a mutated Rev peptide with higher binding affinity and specificity in comparison with the canonical Rev peptide, which binds RRE RNA (88) and is a good model system for studying RNA-protein interactions (Fig. 1 *B*) (25).

Specifically, we conducted cv-SMD simulations along four distinct pathways (defined as PWs; Fig. 1 *C*; Fig. S1; Table S1). In these simulations, we observed the formation of specific interactions and the sequence in which those interactions were forming or rupturing during the dissociation process of the peptide along each pathway, which have not been reported previously. Based on our results, we propose the preferred pathway as well as the mechanism of recognition of the peptide. Additionally, we identified the role of arginine residues in recognition of the peptide by the RRE RNA. Based on our results, we suggest that the atomic scale details on the dissociation process and the recognition mechanism of this peptide by the RRE RNA are potentially useful for designing new therapeutically relevant variants of this peptide.

## Materials and methods

### System setup and equilibration details

In this work, we have studied the (un)binding process of the RSG-1.2 helical peptide from the HIV-1 RRE RNA using steered MD (SMD) simulations along four different pathways (Fig. 1 *C*). We obtained the initial coordinates for our system from the first frame of the NMR structure deposited in the Protein Data Bank (PDB: 1G70) (33). We centered the RNA-peptide complex at the origin and rotated to align the dissociation direction of the peptide in each pathway along the same axis (Fig. S1). We then solvated each system in a periodic simulation domain of three-site transferrable intermolecular potential (TIP3P) water molecules (Fig. S1; Table S1). We neutralized the overall charge of the system with 27 Na^+^ ions.

We energy minimized the system via the steepest descent minimization for 1000 steps that was followed by 500 cycles of conjugate-gradient minimization. To equilibrate the volume of the simulation domain, we conducted a 500-ps MD simulation in the NPT ensemble with a 2-fs timestep. We maintained the temperature and pressure at 310 K and 1 atm using the Langevin thermostat and the Nosé-Hoover barostat in all MD and SMD simulations. We used periodic boundary conditions in all simulations and computed the electrostatic interactions using the particle mesh Ewald method. For the van der Waals interactions, we used a cutoff of 10 Å with switching initiated at 8 Å. We applied weak restraints to the phosphorous atoms in the RNA backbone to prevent the overall rotation and translation of the RNA molecule. We carried out all simulations using the NAnoscale MD (NAMD) (89) software package combined with the AMBER force field for RNA (RNA.ROC, the RNA force-field developed by the Rochester Group) (90) and for the peptide (ff14sb) (91). We used the TIP3P water model (92) for the solvent and the Li-Merz parameters for the ions (93). We analyzed all trajectories using the Visual Molecular Dynamics (VMD) and CPPTRAJ software (94,95).

### Constant velocity cv-SMD simulations

To study the dissociation of the peptide along each of the four pathways, we performed cv-SMD simulations, referred hereafter also as SMD simulations. In cv-SMD simulations, a dummy atom is harmonically coupled to a group of atoms via a virtual spring that is pulled at a constant velocity along a specified direction (reaction coordinate), and the unbinding force is then measured. SMD simulations have been successfully applied to study unfolding of RNA-DNA (96,97) and unbinding mechanisms of protein-ligand (98,99) and RNA-ligand complexes (100, 101, 102) and to study other biophysical systems (103,104). We simulated only the unbinding process of the peptide because of the lack of knowledge of the initial configuration of the unbound RRE-RNA-peptide complex.

To select the four dissociation pathways, we considered a sphere that approximated the volume of the binding pocket (*gray sphere* in Fig. 1 *C*). We then selected points on the surface of the sphere that were radially separated by ∼13 Å to prevent overlap with the RNA molecule. The arrows that are shown in Fig. 1 *C* indicate vectors passing through each of the defined points and represent unique reaction coordinates of dissociation along each of the four pathways. We used the coordinates from the end of the initial MD simulations for subsequent SMD simulations in the NPT ensemble. Specifically, for each of the four pathways, we conducted 75 SMD simulations, each of which was 13 ns long, thereby resulting in a total simulation time of 3900 ns. Our choice of 75 SMD simulations per pathway was based on the convergence of the free-energy profile along each pathway, indicating similar free-energy difference (*ΔG*) between the initial (bound) and the final (dissociated) states. We saved configurations every ps and the SMD output every 20 ps.

Consistent with the stiff-spring approximation (105), we applied a harmonic external force using a spring constant of *k* = 12 kcal/mol Å^2^ that was attached to the center of mass of the peptide residues Gly11 through Ala22, which constitute the helical part of the peptide. We chose this part of the peptide to avoid deviations from the reaction coordinate that could be introduced by the movement of the unfolded segment (residues Ser7 through Ser10). After testing various values, we chose a pulling velocity of 0.00625 Å/ps, which is relatively slower than is commonly used in SMD simulations (0.015–0.02 Å/ps) (106, 107, 108). We also applied a harmonic restraint to prevent the rotation of the peptide to improve convergence of the free-energy profiles (see Supporting materials and methods, Results). As the reference orientation angle, we used the initial coordinates of the peptide, and we used a force constant of 3 kcal/mol deg^2^ for the harmonic potential. We also applied restraints to the atoms forming hydrogen bonds in the peptide residues Gly11 through Ala22 to maintain the secondary structure of the peptide during the dissociation, which prevented the peptide unfolding that could occur in the absence of restraints (see Supporting materials and methods, Results). Although the binding mechanism of the peptide is not fully understood, it was suggested that the peptide is likely partially unfolded in the absence of RRE RNA (33,109). However, in our work, we have focused explicitly on the unbinding process of the peptide while maintaining its secondary structure because resolving both folding and binding processes simultaneously is a challenging task. We also verified that there was no bias introduced by the choice of the ensemble in SMD simulations (see Supporting materials and methods, Results).

### Potential of mean force calculation

We computed the potential of mean force (PMF) as a function of the distance along the reaction coordinate (*r*), which increases at a constant velocity *v* such that *λ*_*t*_ = *λ*_0_ + *vt*, where *λ*_0_ = 0 initially. The *r* vector is commensurate with the pulling direction. The external work performed in a nonequilibrium SMD trajectory is then given by the following equation:

$$W_{0\to t}=-kv\int_{0}^{t}\left(r-\left(\lambda_{0}+vt\right)\right)dt.$$

According to the protocol developed by Jensen et al. (110), we used the exponential averaging of the Jarzynski’s equality (111) to estimate the PMF along the reaction coordinate from work distributions obtained using SMD simulations (105,112). We have previously demonstrated the utility of this approach for studying RNA-ligand interactions (102). The exponential averaging expression is as follows:

$$\Delta G=-\beta^{-1}ln\bar{\text{exp}\left(-\beta W\right)},$$

where *β* = 1/*k*_*B*_*T* with *k*_*B*_ Boltzmann's constant and *T* the temperature, *W* is the nonequilibrium work performed, and *ΔG* is the equilibrium free-energy difference.

### Interaction energies and salt bridges

We also computed the nonbonded interaction energies between a specific amino acid of the peptide and a specific nucleotide of the RRE RNA. In particular, we calculated the van der Waals energy between all atoms in the following pairs of amino acids and nucleotides: Arg8 or R8 and U66; Arg15 or R15 and U72; Arg17 or R17 and A68; and Arg18 or R18 and A68.

We also analyzed a network of hydrogen-bonding and salt-bridging interactions formed between a specific arginine amino acid and a specific RNA nucleotide. Hydrogen bonds were defined between a hydrogen atom of the arginine amino acid and a heavy atom (oxygen or nitrogen atom) of the RNA nucleotide. Salt bridges were defined between a nitrogen atom of the arginine amino acid and the oxygen atom of the phosphate group in the RNA backbone. The definition and the cutoff value of 3.5 Å for hydrogen-bonding and salt-bridging interactions were adopted from a previous study (83). Specifically, we computed the salt bridge distances between the atoms presented in Table S2.

### Solvation of the binding pocket

We also characterized the solvation of the binding pocket during dissociation of the peptide from the RRE RNA. We defined the binding pocket to be comprised of 15 nucleotides (U43, C44, U45, G46, G47, G48, C49, G50, C51, G67, A68, C69, G70, G71, and U72) and computed the number of water molecules that are confined within the volume of the binding pocket formed by these nucleotides.

## Results

### Thermodynamics of peptide dissociation

**Figure 2:**
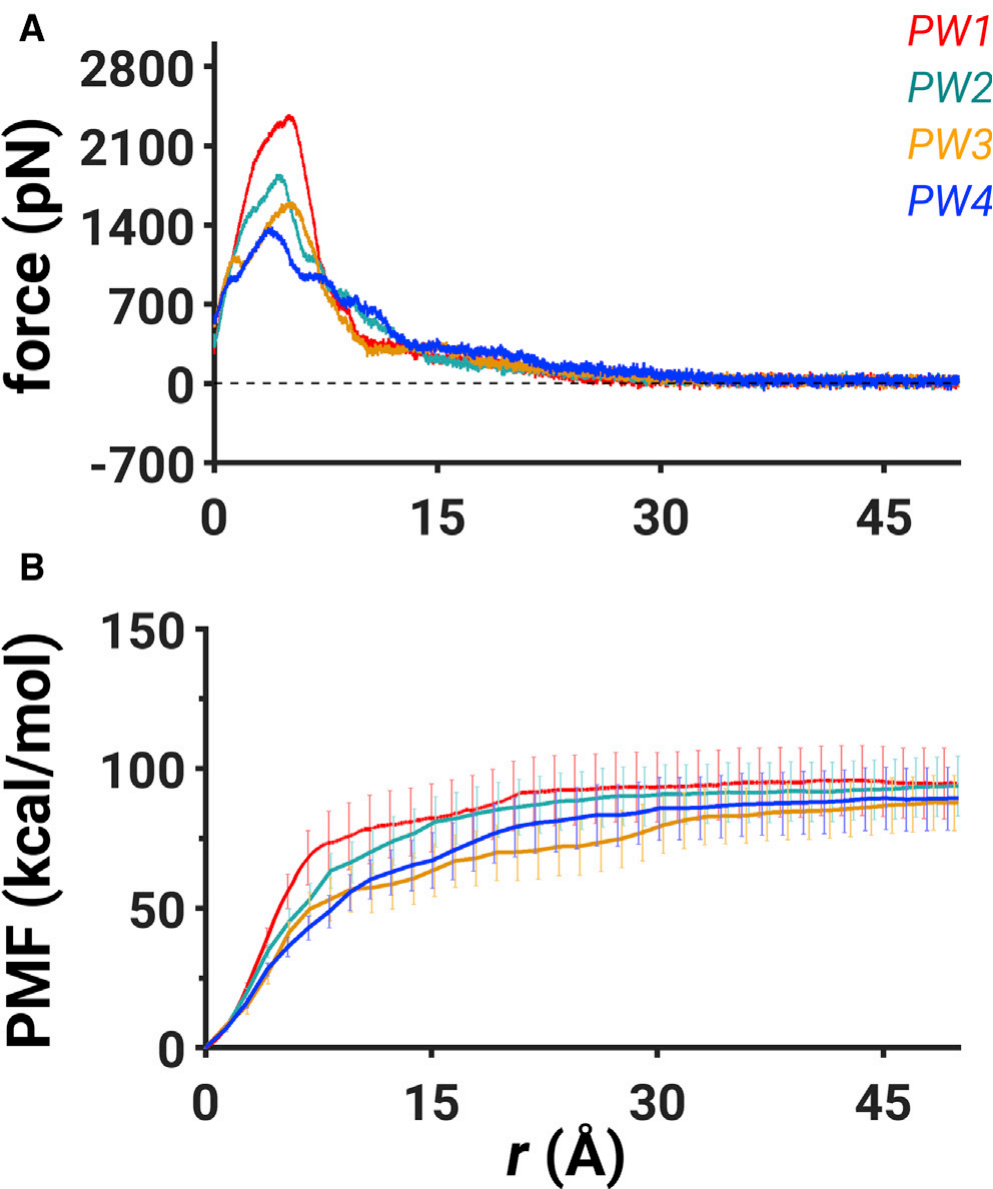
The unbinding force and the free-energy profiles. (*A*) The traces of the averaged unbinding force along each pathway are shown: PW1 (*red*), PW2 (*cyan*), PW3 (*orange*), and PW4 (*blue*). (*B*) The free-energy profile with standard deviations shown as error bars along each pathway is shown. See also Figs. S2 and S6. To see this figure in color, go online.

Using nonequilibrium cv-SMD simulations, we studied the dissociation of the RSG-1.2 peptide from the RRE RNA along four distinct pathways (Fig. 1 *C*). During these SMD simulations, the peptide consistently followed the reaction coordinate (Fig. S2 *A*). We also calculated the unbinding force profiles to ascertain that the average force converged to zero, corresponding to a fully dissociated state of the peptide and with no residual interactions with the RNA. In Fig. S2 *B*, we show the average force profiles with error bars for each pathway, which highlight that the average force for the dissociation of the peptide converged to zero after ∼35–40 Å, depending on the pathway. The convergence to zero is further ascertained by computing the distributions of force values after 40 Å for each pathway that reveal a mean of zero (Fig. S3). Then, we computed the nonequilibrium work required for the dissociation of the peptide from each of the 75 simulations for all four pathways (Figs. S4 and S5). The resulting work distributions were used to estimate the free-energy or PMF profile along the reaction coordinate for each pathway (Fig. 2 *B*) using the Jarzynski’s equality (111), which relates the nonequilibrium work to the equilibrium free-energy difference (*ΔG*). Because nonequilibrium trajectories with the least work have the highest contribution to the equilibrium free-energy difference estimated using the Jarzynski’s equality, we provide mechanistic details from these trajectories.

### Unbinding force profiles

The intermediate steps of the peptide dissociation in each pathway are quantitatively described using the unbinding force profiles (Fig. 2 *A*). At the beginning of each SMD simulation (*r* = 0 Å), the peptide was located in the bound state, interacting with the RNA nucleotides in the binding pocket (Fig. 1 *B*). In particular, the R8 amino acid was initially interacting with the U66, G64, and A52 nucleotides; the R14 amino acid was interacting with the G70 nucleotide; R15 was interacting with the A73 and U72 nucleotides; and the R17 amino acid was initially interacting with the A68 nucleotide (Fig. 1 *B*). A gradual increase in the external force values for each pathway (Fig. 2 *A*) indicates that the peptide began to dissociate from the binding pocket by overcoming the interactions with the binding pocket nucleotides. The peak force values correspond to the stage when the peptide has moved out of the binding pocket by rupturing key interactions with the RNA. The external force values then decreased as the peptide was at a distance of ∼35–40 Å when the force values on average converged to zero, signifying that the peptide reached the dissociated state (Fig. 2 *A*).

We further analyzed the unbinding force profiles that exhibited different magnitudes of the maximal force of dissociation in each pathway. Specifically, we observed that PW1 had the highest value of the maximal force (∼2377 pN) of dissociation occurring at ∼5 Å (*PW1* in Fig. 2 *A*). The force profile in PW2 exhibited the second highest value of the maximal force (∼1850 pN) of dissociation at ∼4.5 Å (*PW2* in Fig. 2 *A*). Additionally, we detected a smaller peak of the unbinding force (∼1090 pN) at ∼7.2 Å in PW2. We observed that PW3 exhibited the third highest value of the maximal force (∼1600 pN) of dissociation at ∼5.2 Å (*PW3* in Fig. 2 *A*). Moreover, we detected a smaller force peak value at 1.3 Å in PW3 corresponding to ∼1120 pN. Finally, we observed the lowest value of the maximal force (∼1360 pN) of dissociation in PW4, which occurred at ∼3.8 Å (*PW4* in Fig. 2 *A*). We also located smaller peaks in force at ∼1.2 Å and at ∼6.6 Å, which were both equal to ∼950 pN. We detected a variability in the location of the maximal force value in the individual trajectories. The maximal force values were located between 4.6 and 5.4 Å in PW1, between 4.3 and 4.8 Å in PW2, between 4.9 and 5.4 Å in PW3, and between 3.6 and 4.5 Å in PW4 (Fig. S2 B). We observed that the unbinding force profiles converged to zero at 35 Å for PW1 and PW2 and at 40 Å for PW3 and PW4 (Fig. 2 *A*; Fig. S2 *B*).

### Free-energy profiles

We report the free-energy profiles for each pathway (Fig. 2 *B*) that provide additional information on the thermodynamics of peptide dissociation, including the free-energy barriers and the metastable states. All reported free-energy values are measured with respect to the initial state. We also show a zoomed view of each free-energy profile for *r*-values between 0 and 15 Å along with the first-order derivative (*m*) of the free-energy profile computed for the same range of *r*-values in each pathway (Fig. S6). The first-order derivative provides information on the rate of change of the free-energy profile and assists in identifying metastable states (*labeled M*, Fig. S6) as well as the free-energy barriers (*labeled double dagger*, Fig. S6). The first-order derivative converges to zero, corresponding to the region of the free-energy or the PMF profile when there is no significant change in the PMF.

We observed that the highest free-energy barrier of dissociation was in PW1, which was equal to 41 ± 3.67 kcal/mol at ∼4.2 Å with an additional free-energy barrier of 61.67 ± 7.41 kcal/mol at 6 Å (*red double dagger*, Fig. S6 *A*). We observed the second highest free-energy barrier in PW2 corresponding to 37.51 ± 2.62 kcal/mol at ∼4.4 Å with an additional free-energy barrier of 58.08 ± 5.96 kcal/mol at ∼7.5 Å (*cyan double dagger*, Fig. S6 *B*). In PW3, we observed several free-energy barriers at ∼1 Å and at ∼4.6 Å, corresponding to the free-energy values of 4.49 ± 0.18 kcal/mol and 31.47 ± 3.77 kcal/mol, respectively (*orange double dagger*, Fig. S6 *C*). Finally, in PW4, we observed four free-energy barriers at ∼0.8, ∼3.8, ∼5.9, and ∼8.5 Å, corresponding to the free-energy values of 3.66 ± 0.34, 24.46 ± 2.08, 38.46 ± 3.59, and 50.48 ± 5.99 kcal/mol (*blue double dagger*, Fig. S6 *D*).

We also observed the formation of metastable states along different pathways (*labeled M* in Fig. S6). We located the metastable states at ∼5.4 Å in PW1 (*red M*, Fig. S6 *A*), ∼5.4 Å in PW2 (*cyan M*, Fig. S6 *B*), 1.8 Å in PW3 (*orange M*, Fig. S6 *C*), and 1.3, 5.1, and 6.9 Å in PW4 (*blue M*, Fig. S6 *D*). The mechanistic details of each metastable state are provided in the following section. Finally, we observed that the free-energy differences between the initial states (*r* = 0 Å) and the dissociated states (*r* = 50 Å) were 96.47 ± 12.63 kcal/mol for PW1, 96.1 ± 10.95 kcal/mol for PW2, 91.83 ± 9.81 kcal/mol for PW3, and 92 ± 11.32 kcal/mol for PW4. Thus, the resulting free-energy differences (*ΔG*) have similar values, falling within the range of error bars for each pathway. Overall, we observed that PW4 has the smallest free-energy barrier for dissociation of the peptide while having additional metastable states in comparison with other pathways.

### Mechanistic details of peptide dissociation pathways

In the initial conformation, the peptide is bound in the major groove of the RRE RNA between the A75-U45 and U66-A52 basepairs while largely maintaining an *α*-helical conformation with five residues constituting a coiled segment at the N-terminus (Fig. 1 *B*) (33). The A68 and U72 nucleotides were in the flipped-out configurations, recognizing the peptide through stacking interactions with the R15 and R18 amino acids, respectively (Fig. 1 *B*). The Hoogsteen edge of the G70 and A73 nucleotides formed hydrogen-bonding interactions with the R14 and R15 amino acids, respectively. The R8 amino acid from the coiled segment of the peptide interacts with the U66 nucleotide, whereas the R17 and R18 amino acids also form contacts with the RNA backbone.

### Pathway 1

**Figure 3:**
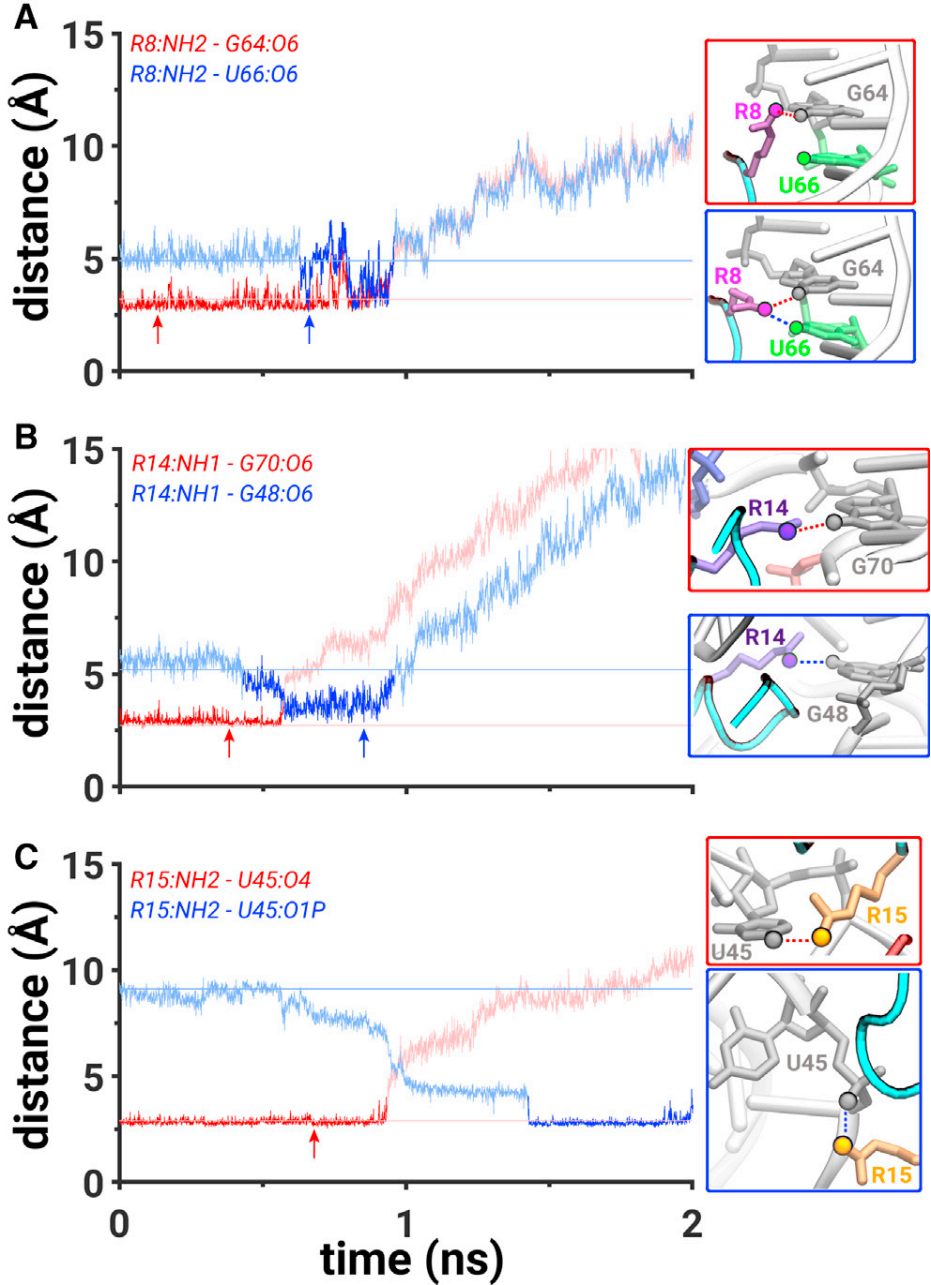
Mechanistic details of PW1. (*A*) The hydrogen bond distances between the NH2 atom of R8 and the O6 atom of G64 (*red trace*) and between the NH2 atom of R8 and the O6 atom of U66 (*blue trace*). (*B*) The hydrogen bond distances between the NH1 atom of R14 and the O6 atom of G70 (*red trace*) and between the NH1 atom of R14 and the O6 atom of G48 (*blue trace*). (*C*) The hydrogen bond distance between the NH2 atom of R15 and the O4 atom of U45 (*red trace*) and the salt bridge between the NH2 atom of R15 and the O1P atom of U45 (*blue trace*). All metrics are computed from the simulation with the lowest-work value. Darker colors signify the regions of interest. Lightly shaded horizontal lines indicate the initial values of the corresponding distances. Each panel is accompanied with snapshots highlighting the corresponding interactions extracted from a time point marked by an arrow. Each amino acid, nucleotide, and atom that participates in hydrogen-bonding or salt-bridging interactions is uniquely colored. The scale on *y* axis is limited to 15 Å because the presented interactions form at distances below 3.5 Å. The data corresponding to all distance values on the *y* axis are shown in Fig. S9*A*. To see this figure in color, go online.

During the early part of the lowest-work SMD simulation in PW1, the peptide began dissociating out of the binding pocket (Fig. S7 *A*), which was also characterized by weakening of interactions between several key amino acids and nucleotides (Fig. S8 *A*). In particular, we observed that the van der Waals interaction energy between the R8 amino acid and the U66 nucleotide, the R15 amino acid and the U72 nucleotide, and the R17 amino acid and the A68 nucleotide approached zero (Fig. S8 *A*), indicating negligible interactions between the residues. Specifically, at t = ∼0.6 ns, the hydrogen bond between the NH2 atom of R8 amino acid and the O6 atom of G64 weakened (*red trace*, Fig. 3 *A*), and a new hydrogen bond was formed between the NH2 atom of R8 amino acid and the O6 atom of U66 (*blue trace*, Fig. 3 *A*). Additionally, at t = ∼0.6 ns, the hydrogen bond between the NH1 atom of R14 amino acid and the O6 atom of G70 broke (*red trace*, Fig. 3 *B*), which led to the formation of a hydrogen bond between the NH1 atom of R14 amino acid and the O6 atom of G48 (*blue trace*, Fig. 3 *B*). This sequence of events was a result of the peptide leaving the initial binding pocket, which was coupled with the formation of new hydrogen-bonding interactions between the R8 and R14 amino acids and the U66 and G48 nucleotides, respectively (Fig. 3*, A and B*).

At t = ∼1 ns, the peptide was located in the proximity of the backbone atoms of the C44, U45, and G46 nucleotides that constitute the major groove of the RNA (Fig. S10) and the van der Waals interactions between the R8, R15, and R17 amino acids, and the U66, U72, and A68 nucleotides diminished (Fig. S8 *A*). This was also characterized by the rupture of the hydrogen bonds that were previously formed at t = ∼0.6 ns between the NH2 atom of R8 amino acid and the O6 atom of U66 and between the NH1 atom of R14 amino acid and the O6 atom of G48 (*blue trace*, Fig. 3*, A and B*). The state when the peptide was located in the proximity of the backbone atoms of the C44, U45, and G46 nucleotides corresponds to a weak metastable state in the free-energy profile (*red M*, Fig. S6 *A*).

At t = ∼1.4 ns, the peptide displaced the backbone atoms of the C44, U45, and G46 nucleotides and was located in a partially dissociated state, whereas the R8, R14, and R15 amino acids were still in the vicinity of the RRE RNA with the possibility to interact with the C44, U45, and G46 nucleotides (Fig. S7 *A*). However, at t = ∼1.9 ns, we observed the formation of only one salt bridge that was formed between the NH2 atom of R15 amino acid and the O1P atom of U45 (*blue trace*, Fig. 3 *C*), which was preceded by the rupture of the hydrogen bond at t = ∼0.95 ns between the NH2 atom of R15 amino acid and the O4 atom of U45 while the peptide was still located in the binding pocket (*red trace*, Fig. 3 *C*). The peptide was free of any interactions with the RNA at a distance of 35 Å (t = 5.6 ns).

### Pathway 2

**Figure 4:**
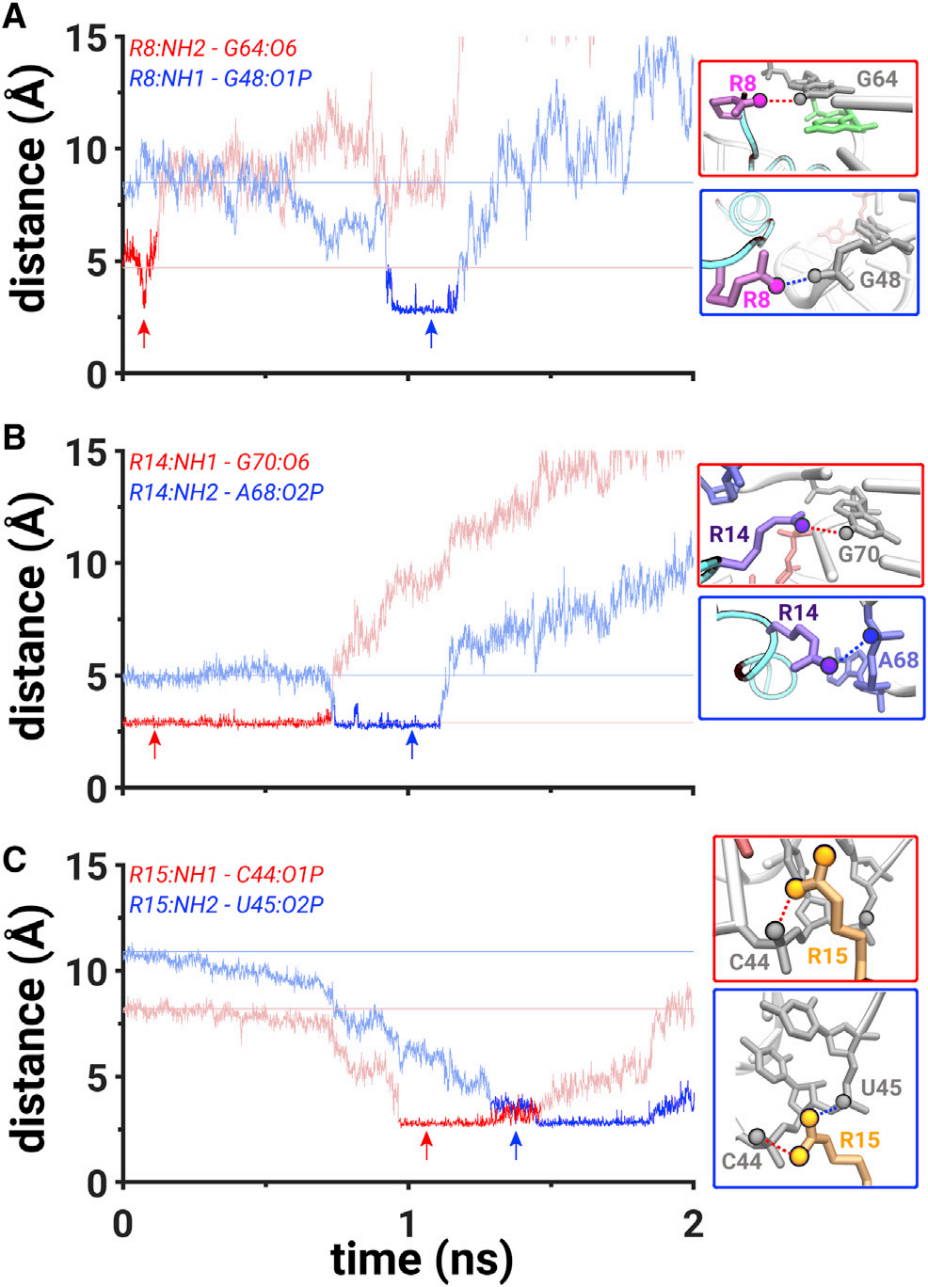
Mechanistic details of PW2. (*A*) The hydrogen bond distance between the NH2 atom of R8 and the O6 atom of G64 (*red trace*) and the salt bridge between the NH1 atom of R8 and the O1P atom of G48 (*blue trace*). (*B*) The hydrogen bond distance between the NH1 atom of R14 and the O6 atom of G70 (*red trace*) and the salt bridge between the NH2 atom of R14 and the O2P atom of A68 (*blue trace*). (*C*) The salt bridges between NH1 atom of R15 and the O1P atom of C44 (*red trace*) and between the NH2 atom of R15 and the O2P atom of U45 (*blue trace*). The scale on *y* axis is limited to 15 Å because the presented interactions form at distances below 3.5 Å. The data corresponding to all distance values on the *y* axis are shown in Fig. S9*B*; cf. Fig. 3 for all other details. To see this figure in color, go online.

In PW2, we observed different mechanistic details underlying the dissociation process in comparison with PW1, which likely contributed to a lower free-energy barrier to dissociation (Fig. 2 *B*). As the peptide began dissociating out of the binding pocket (Fig. S7 *B*), the van der Waals interactions between the R8 amino acid and the U66 nucleotide were broken at t = ∼0.1 ns (*purple trace*, Fig. S8 *B*). This event occurred simultaneously with the rupture of the hydrogen bond between the NH2 atom of R8 amino acid and the O6 atom of G64 at t = ∼0.1 ns (*red*, Fig. 4 *A*). The R8 amino acid did not form any stable close contact interactions until t = ∼0.9 ns, when the NH1 atom of R8 formed a salt bridge with the O1P atom of G48. At t = ∼0.73 ns, the hydrogen bond between the NH1 atom of R14 and the O6 atom of G70 (which was preformed in the initial binding pocket) broke, and the NH2 atom of R14 formed a salt bridge with the O2P atom of A68 at t = ∼0.75 ns (Fig. 4 *B*). Thus, two arginine amino acids, R8 and R14, formed salt-bridging interactions at t = ∼0.9 ns, creating a network of salt bridges from the G48 nucleotide to the A68 nucleotide (Fig. S11 *A*). This conformation also resulted in a metastable state that was highlighted in the free-energy profile at ∼5.4 Å (*cyan M*, Fig. S6 *B*).

In PW2, the NH1 atom of R15 formed a salt bridge with the O1P atom of C44 (*red trace*, Fig. 4 *C*) when the peptide was in the vicinity of the backbone atoms of the C44, U45, and G46 nucleotides at t = ∼1 ns (Fig. S7 *B*). Importantly, at t = ∼1.3 ns, the NH2 atom of R15 formed a salt bridge with the O2P atom of U45 (*blue trace*, Fig. 4 *C*). Thus, between t = ∼1.3 and t = ∼1.5 ns, the NH1 and NH2 atoms of R15 were fluctuating to simultaneously form two salt bridges with the O1P and O2P atoms of C44 and U45 nucleotides, respectively (Fig. 4 *C*). This motion was another factor that contributed to a decrease in the free-energy barrier in comparison with PW1. In addition to that, the rupture of the hydrogen bond between the NH2 atom of R8 amino acid and the O6 atom of G64 at t = ∼0.1 ns and the rupture of the van der Waals interactions between the R8 amino acid and the U66 at t = ∼0.1 ns also contributed to a decrease in the free-energy barrier in comparison with PW1. The peptide was free of any interactions with the RNA at a distance of 35 Å (t = 5.6 ns).

### Pathway 3

In PW3, the peptide required ∼3 ns to escape the binding pocket, whereas in PW1 and PW2, the peptide escaped the binding pocket in ∼2 ns (Fig. S7*, A–C*). This was in part due to the interactions of various amino acids with the A68 nucleotide in PW3 (Fig. S7 *C*) as well as due to the interactions between the R8 amino acid and the U66 nucleotide that we characterized using the van der Waals energy (*purple trace*, Fig. S8 *C*). These interactions resulted in a partial unfolding of the peptide coil between t = ∼1.8 and t = ∼3 ns (Fig. S7 *C*).

**Figure 5:**
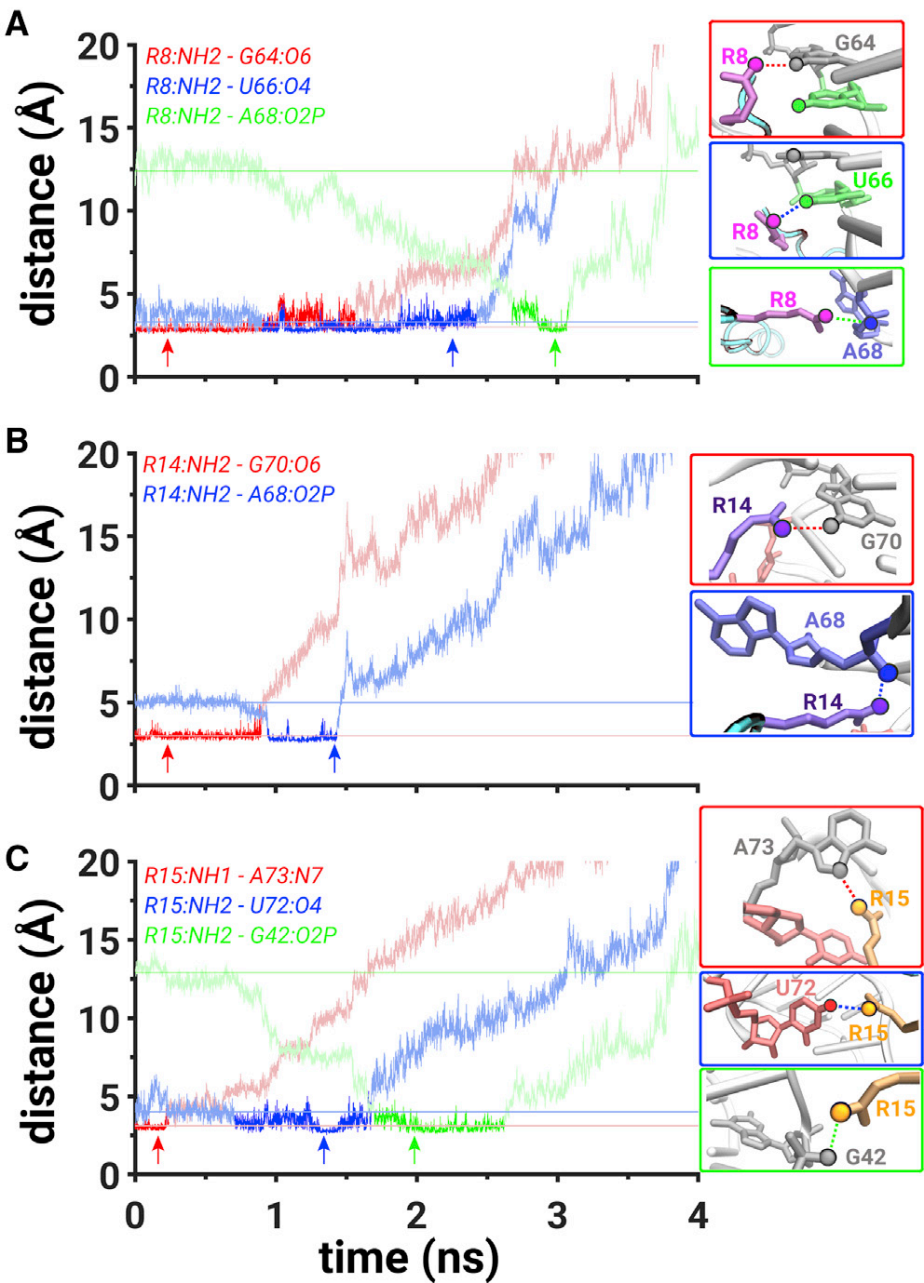
Mechanistic details of PW3. (*A*) The hydrogen bond distances between the NH2 atom of R8 and the O6 atom G64 (*red trace*) and between the NH2 atom R8 and the O4 atom of U66 (*blue trace*) and the salt bridge between the NH2 atom of R8 and the O2P atom of A68 (*green trace*). (*B*) The hydrogen bond distance between the NH2 atom of R14 and the O6 atom of G70 (*red trace*) and the salt bridge between the NH2 atom of R14 and the O2P atom of A68 (*blue trace*). (*C*) The hydrogen bond distances between the NH1 atom R15 and the N7 atom of A73 (*red trace*) and between the NH2 atom of R15 and the O4 atom of U72 (*blue trace*) and the salt bridge between the NH2 atom of R15 and the O2P atom of G42 (*green trace*). The scale on *y* axis is limited to 20 Å because the presented interactions form at distances below 3.5 Å. The data corresponding to all values of distances on the *y* axis are shown in Fig. S9*C*; cf. Fig. 3 for all other details. To see this figure in color, go online.

During the first 0.8 ns of the simulation, the peptide disrupted interactions between the R18 amino acid and the A68 nucleotide, as characterized by the van der Waals energy (*brown trace*, Fig. S8 *C*), and started dissociating. A hydrogen bond between the NH2 atom of R8 amino acid and the O6 atom of G64 weakened at t = ∼0.9 ns (*red trace*, Fig. 5 *A*) and the NH2 atom of R8 amino acid started forming a new hydrogen bond with the O4 atom of U66 at t = ∼1 ns (*blue trace*, Fig. 5 *A*). At t = ∼0.9 ns, the hydrogen bond between the NH2 atom of R14 amino acid and the O6 atom of G70 ruptured (*red trace*, Fig. 5 *B*) and the NH2 atom of R14 amino acid formed a salt bridge with the O2P atom of A68 (*blue trace*, Fig. 5 *B*).

At t = ∼0.25 ns, the NH1 atom of R15 amino acid stopped forming the hydrogen bond with the N7 atom of A73, and the NH2 atom of R15 formed a hydrogen bond with the O4 atom of U72. Thus, the combined interactions between the NH2 atom of R8 amino acid and the O4 atom of U66, between the NH2 atom of R14 amino acid and the O2P atom of A68, and between the NH2 atom of R15 amino acid and the O4 atom of U72 created a network of salt-bridging and hydrogen-bonding interactions at ∼1 ns and lasted for ∼0.5 ns (Fig. S11 *B*).

At t = ∼1.7 ns, the hydrogen bond between the NH2 atom of R15 amino acid and the O4 atom of U72 ruptured (*blue trace*, Fig. 5 *C*), and a salt bridge was formed between the NH2 atom of R15 and the O2P atom of the G42, which broke at t = ∼2.7 ns (*green trace*, Fig. 5 *C*). At t = ∼2.7 ns, a salt bridge was formed between the NH2 atom of R8 amino acid and the O2P atom of A68 that lasted for ∼0.2 ns (*green trace*, Fig. 5 *A*). Thus, salt-bridging interactions were forming during every step of the dissociation process in PW3. The peptide was free of any interactions with the RNA at a distance of 40 Å (t = 6.4 ns).

### Pathway 4

**Figure 6:**
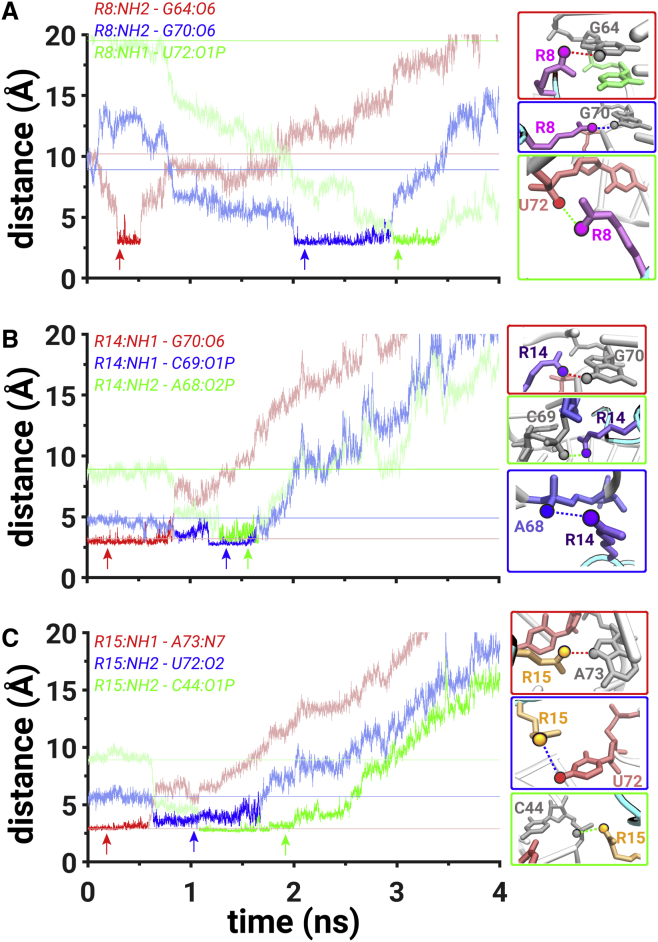
Mechanistic details of PW4. (*A*) The hydrogen bond distances between the NH2 atom of R8 and the O6 atom of G64 (*red trace*) and between the NH2 atom of R8 and the O6 atom of G70 (*blue trace*) and the salt bridge between the NH1 atom of R8 and the O1P atom of U72 (*green trace*). (*B*) The hydrogen bond distance between the NH1 atom of R14 and the O6 atom of G70 (*red trace*) and the salt bridges between the NH1 atom of R14 and the O1P atom of C69 (*blue trace*) and between the NH2 atom of R14 and the O2P atom of A68 (*green trace*). (*C*) The hydrogen bond distances between NH1 atom of R15 and the N7 atom of A73 (*red trace*) and between the NH2 atom of R15 and the O2 atom of U72 (*blue trace*) and the salt bridge between the NH2 atom of R15 and the O1P atom of C44 (*green trace*). The scale on *y* axis is limited to 20 Å because the presented interactions form at distances below 3.5 Å. The data corresponding to all distance values on the *y* axis are shown in Fig. S9*D*; cf. Fig. 3 for all other details. To see this figure in color, go online.

Finally, in PW4, which had the lowest free-energy barrier to dissociation (Fig. S6 *D*), the mechanism of dissociation was similar to PW3, but we observed several key differences. During the first 0.8 ns of the simulation, the interactions between the R8 amino acid and the U66 nucleotide and the R18 amino acid and the A68 nucleotide weakened, as characterized by the van der Waals interaction energy (Fig. S8 *D*). The NH2 atom of R8 amino acid formed a hydrogen bond with the O6 atom of G64 at t = ∼0.35 ns and broke it at t = ∼0.55 ns (*red trace*, Fig. 6 *A*). After that, the R8 amino acid did not form any stable interactions until t = ∼2 ns (Fig. S7 *D*). At t = ∼0.8 ns, the hydrogen bond between the NH1 atom of R14 amino acid and the O6 atom of G70 ruptured (*red trace*, Fig. 6 *B*), and a salt bridge was formed between the NH1 atom of R14 amino acid and the O1P atom of G69 (*blue trace*, Fig. 6 *B*). At t = ∼0.65 ns, a hydrogen bond was formed between the NH2 atom of R15 amino acid and the O2 atom of U72 (*blue trace*, Fig. 6 *C*), which was preceded by the rupture of the hydrogen bond (at t = ∼0.6 ns) between the NH1 atom of R15 amino acid and the N7 atom of A73 (*red trace*, Fig. 6 *A*). The salt bridge between the NH1 atoms of R14 amino acid with the O1P atom of C69 and the hydrogen bond between the NH2 atom of R15 amino acid with the O2 atom of U72 formed a network of salt-bridging and hydrogen-bonding interactions at ∼0.8 ns (Fig. S11 *C*), which corresponded to a metastable state at ∼5.1 Å (*blue M*, Fig. S6 *D*).

At t = ∼1.3 ns, the NH2 atom of R14 amino acid formed another salt bridge with the O2P atom of A68 (*green trace*, Fig. 6 *B*). The NH2 atom of R15 amino acid ruptured the hydrogen bond with the O2 atom of U72 and formed a salt bridge with the O1P atom of C44 at t = ∼1.1 ns (Fig. 6 *C*). At t = ∼2 ns, the NH2 atom of R8 amino acid formed a hydrogen bond with the O6 atom of G70 (*blue trace*, Fig. 6 *A*) and combined with the salt bridge between the NH2 atom of R15 amino acid and the O1P atom of C44; the second network of hydrogen-bonding and salt-bridging interactions was created in PW4 (Document S1. Supporting materials and methods, Tables S1–S2, and Figs. S1–S16, Document S2. Article plus supporting material *D*) and corresponded to a metastable state at ∼6.9 Å (*blue M*, Fig. S6 *D*). At t = ∼2.4 ns, a hydrogen bond between the NH2 atom of R8 amino acid and the O6 atom of G70 ruptured, and a salt bridge was formed between the NH1 atom of R8 and the O1P atom of U72 (*green trace*, Fig. 6 *A*). The peptide was free of any interactions with the RNA at a distance of 40 Å (*t* = 6.4 ns).

Overall, we observed the formation of unique interactions in each pathway, including the formation of salt-bridging and hydrogen-bonding interactions. Our observations suggest that there is a network of salt bridges and hydrogen bonds that was formed in each pathway, with the exception of PW1, which had the smallest number of hydrogen bonds and salt bridges formed in comparison with other pathways.

### Water dynamics in the binding pocket

Water is known to play a critical role in ligand recognition (113) because ligand binding-unbinding may be accompanied by loss or gain of water molecules in the binding pocket. Therefore, we investigated the solvation of the binding pocket of the RRE RNA during peptide dissociation along two pathways (PW1 and PW4). These pathways were identified to have the highest and the lowest free-energy barriers, respectively, for dissociation of the peptide. We sought to understand the role of water molecules and their contribution to differences in the free-energy barriers along these two pathways.

Before the dissociation process was initiated, we observed around 40 water molecules around the peptide in the binding pocket of the RRE RNA (Fig. S12). As the peptide dissociated along PW1, we initially observed a slow increase in the number of water molecules (N_W_) to 50 water molecules, signifying the influx of water in the binding pocket. Between t = ∼0.65 and t = ∼0.85 ns, the value of N_W_ did not significantly change, which was also characterized by the formation and further rupture of the hydrogen bonds between the NH2 atom of the R8 amino acid and the O6 atoms of the G64 and U66 nucleotides (Fig. 3 *A*, Fig. S12). At t = ∼0.9, we observed a more rapid increase in N_W_ that converged to ∼110 water molecules at t = ∼1.4 ns when the peptide dissociated from the binding pocket (*red trace*, Fig. S12). We also noticed a small decrease in N_W_ at t = ∼1.05 ns that was coupled with the interaction between the residues R8, R14, and R15 and the RNA backbone (Fig. S10).

However, we observed a different solvation pattern of the binding pocket in PW4 in comparison with PW1. Specifically, we initially observed desolvation of the binding site because the N_W_-value decreased to ∼30 water molecules until t = ∼0.5 ns (*blue trace*, Fig. S12). This decrease was coupled with the movement of the peptide out of the binding pocket that led to water molecules moving out of the pocket because of the rearrangements in the amino acids. Specifically, the R8 residue was dynamic and moved in the binding pocket until the NH2 atom of R8 formed a hydrogen bond with the O6 atom of G64 (*red trace*, Fig. 6 *A*). At t = ∼0.5 ns, the N_W_-values started to increase, indicating the resolvation of the binding site (*blue trace*, Fig. S12). The resolvation continued until t = ∼1.9 ns, when the main portion of the peptide escaped the binding pocket, whereas the unstructured coil segment of the peptide still remained in the binding pocket interacting with the nucleotides (Figs. S7 and S12). After the last amino acid escaped the binding pocket at t = ∼3.2 ns, the binding pocket was fully solvated. Importantly, the final N_W_-values for both pathways converged to similar number of water molecules in the binding pocket. Because we observed differences in the free-energy barriers of PW1 and PW4 during the earlier stage of dissociation of the peptide, the distinct water dynamics in PW4, where the binding pocket initially desolvated before resolvating, potentially contributed to a lower free-energy barrier for peptide dissociation.

## Discussion

In this work, we have studied the dissociation mechanism of the RSG-1.2 peptide from the RRE RNA along four distinct pathways using nonequilibrium SMD simulations. Although it has been previously proposed that the salt-bridging interactions could be important for the recognition of this peptide by the RRE RNA (83), there is no study on the binding-unbinding mechanism of this peptide in the literature. We observed the formation of unique salt-bridging and hydrogen-bonding interactions in each pathway that form in an ordered stepwise sequence in which the rupture of one interaction led to the creation of another interaction. We also estimated the free-energy profiles for each pathway using the Jarzynski’s equality and observed distinct free-energy barriers in each pathway.

We observed the highest free-energy barrier of peptide dissociation in PW1 (Fig. 2 *B*), which was coupled with the displacement of the backbone atoms of the C44, U45, and G46 nucleotides (Fig. S10). Moreover, we observed only one salt bridge formed during dissociation in PW1 (Fig. 3 *C*; Table S2). The formation of this salt bridge between the R15 amino acid of the peptide and the U45 nucleotide of the RRE RNA was coupled with a weak recognition of the peptide by the RNA. This pathway was also characterized by a rapid solvation of the binding pocket (*red trace*, Fig. S12).

Even though the overall process of dissociation in PW2 was somewhat similar to PW1, the free-energy barrier in PW2 was smaller than in PW1 (Fig. S6*, A and B*). One of the key differences between PW1 and PW2 was the interaction between the R8 amino acid and the U66 nucleotide of the RNA that ruptured at t = ∼0.1 ns in PW2, as characterized by the van der Waals interaction energy, whereas the rupture of the interaction between the R8 amino acid and the U66 nucleotide only occurred at t = ∼0.8 ns in PW1 (*purple traces*, Fig. S8*, A and B*). We also observed an additional salt bridge in PW2 that was formed between the NH2 atom of R15 amino acid and the O1P atom of C44 (*red trace*, Fig. 4 *C*). This interaction was formed ∼0.5 ns earlier in PW2 in comparison with a similar type of interaction between the NH2 atom of R15 amino acid and the O1P atom of U45 in PW1. The peptide passed in close proximity to the C44 and U45 nucleotides in both pathways, and a faster establishment of a salt-bridging interaction with an atom from one of these nucleotides is important for the recognition of the RNA backbone for the peptide if it dissociates along PW1 or PW2. The earlier rupture of the interaction between the R8 amino acid and the U66 nucleotide as well as a lack of displacement of the backbone atoms of the C44, U45, and G46 nucleotides led to a decreased free-energy barrier in PW2.

The pathways PW3 and PW4 had smaller free-energy barriers in comparison with PW1 and PW2 (Fig. S6). It should be noted that in PW3 and PW4, the peptide required a longer time to dissociate in comparison with PW1 and PW2, which was caused by additional interactions that were forming between the flipped-out A68 nucleotide and the peptide as it was dissociating (Fig. S7*, C and D*). These interactions were not formed in PW1 and PW2 because the peptide was dissociating in a direction away from the A68 nucleotide (Fig. S7*, A and B*). Moreover, the dissociation reaction coordinate in PW3 and PW4 was free of any obstacles, such as the atoms of the RNA backbone in the C44, U45, and G46 nucleotides that were present in PW1 and PW2. Thus, a decrease in the free-energy barriers in PW3 and PW4 was achieved by reducing any steric overlap or the displacement of the atoms in the major groove of the RNA. Therefore, these two pathways, PW3 and PW4, are preferred in comparison with PW1 and PW2 due to lower free-energy barriers for peptide dissociation (Fig. S6). We also observed desolvation of the binding pocket (*blue trace*, Fig. S12) during the initial stages of dissociation of the peptide, which likely contributed to a lower free-energy barrier in comparison with other pathways.

However, PW4 exhibited an even smaller free-energy barrier of dissociation by ∼7 kcal/mol in comparison with PW3, meaning that the pathway PW4 is further preferred over PW3. We also conducted umbrella sampling (114) simulations (see Supporting materials and methods, Methods) to recompute the free-energy profile and determine the free-energy barrier for dissociation of the peptide along PW4 (Fig. S13). These results showed that the free-energy barrier along PW4 was ∼23.72 ± 1.46 kcal/mol, which was comparable with the value (24.46 kcal/mol) that we obtained using SMD simulations. We further tested the evolution of the reaction coordinate along PW4 using the swarms-of-trajectories string method (115) to verify that PW4 was a reliable pathway (see Supporting materials and methods, Methods). We computed the average root mean squared deviation (RMSD) of the entire string across each iteration as a measure of convergence of the string (Fig. S14). The initial pathway generated using SMD converged after 13 iterations, whereas the main fluctuations in the string RMSD were primarily due to the motion of the peptide in the bulk water (Fig. S14). Therefore, these additional tests indicate that the reaction coordinate along PW4 is reasonable and accurate for understanding thermodynamics of dissociation of the peptide. We conducted umbrella sampling and swarms-of-trajectories string method calculations using the AMBER software package (116) combined with the AMBER force field for RNA (RNA.ROC) (90) and for the peptide (ff14sb) (91). Additionally, we note that the usage of rotational restraints improves convergence of the free-energy profile (Fig. S15 *A*); the usage of secondary structure restraints prevents the unfolding of the peptide (Fig. S15 *B*), whereas the choice of the ensemble used in SMD simulations does not significantly affect the free-energy profile (Fig. S15 *C*).

The decrease in free-energy barrier in PW4 is likely a result of the behavior of the R8 amino acid, which did not form any stable interactions between ∼0.5 and ∼2 ns in PW4 (Fig. 6 *A*) while it was forming stable hydrogen-bonding interactions in that time range in PW3 (*red* and *blue traces*, Fig. 5 *A*). This behavior of the R8 amino acid was also reflected in the van der Waals interaction energies (*purple traces*, Fig. S8*, C and D*), which showed that the R8 amino acid had stronger interactions with the U66 nucleotide in PW3 in comparison with PW4.

By analyzing the salt-bridging and hydrogen-bonding interactions in each pathway, we determined that R8, R14, and R15 were the most critical amino acids for the recognition of the peptide by the RRE RNA. Each of these amino acids were involved in a complex network of salt bridges and hydrogen bonds in PW2, PW3, and PW4 (Figs. 4, 5, and 6; Fig. S11). Moreover, these amino acids interacted with the RNA nucleotides in a stepwise pattern in which the rupture of existing interactions resulted in the formation of new interactions with other nucleotides during the dissociation process. PW3 and PW4 exhibited the formation of additional hydrogen bonds and salt bridges in comparison with PW1 and PW2, which resulted from the extended dissociation timescales. Furthermore, we recomputed the free-energy profile (Fig. S16) along PW4 by mutating each of the three key arginine residues (R8, R14, and R15) to alanine residues using the psfgen plugin in VMD (95). These results showed a decrease in the free-energy barrier on mutations of key arginine residues, indicating that the peptide is significantly destabilized without interactions of these residues with the RRE RNA, thereby supporting their critical role in binding of this peptide.

In particular, we believe that the R8 amino acid was the most critical amino acid in the least free-energy barrier pathway PW4. Firstly, as mentioned before, the R8 amino acid had decreased interactions with the nucleotides of the RRE RNA between ∼0.5 and ∼2 ns, which led to a decreased free-energy barrier in PW4. Secondly, after the peptide dissociated from the initial binding pocket, the R8 amino acid was the only amino acid that was forming a stable interaction with the RNA nucleotide after ∼3 ns. Specifically, the NH2 atom of the R8 amino acid formed a salt bridge with the O1P atom of the U72 nucleotide between ∼3 and ∼3.4 ns (*green trace*, Fig. 6 *A*). Thus, we hypothesize that for the reverse process of peptide binding along PW4, the R8 amino acid will be the first amino acid to form a stable interaction with the U72 nucleotide of the RRE RNA.

Additionally, it is critical to note that the RSG-1.2 protein was synthesized by mutagenesis from the Rev peptide (109) that binds the RRE RNA during the HIV-1 replication process. One important mutation in that study was the mutation of the arginine amino acid in the Rev protein at position 9 to a proline amino acid. It was hypothesized that this mutation resulted in a decrease in electrostatic contacts between the arginine amino acids in the N-terminus of the peptide and could be potentially coupled with the increased binding affinity to the RRE RNA (109). However, it was not clear how the RSG-1.2 peptide recognized the RRE RNA during the binding process and which amino acids contributed the most to this process. In our work, we observed that the R8 amino acid, which is located next to the Rev protein at position 9 amino acid in the polypeptide chain, formed stable hydrogen-bonding and salt-bridging interactions in each pathway. The R8 amino acid also was the last amino acid to interact with the RRE RNA during the dissociation and thus could be the first to interact with the RRE RNA during the binding process. Thus, the ability of the R8 amino acid to form these interactions was rooted in its flexibility that was coupled with the formation of various interactions with the RRE RNA nucleotides and resulted in the increased binding affinity and specificity with the RRE RNA in comparison with the Rev protein.

## Conclusions

The binding-unbinding of proteins or short peptides to RNA is an important biophysical process that is poorly understood. We used nonequilibrium cv-SMD simulations to study the dissociation mechanism of a helical peptide along four different pathways from the RRE RNA binding pocket to obtain key insights into the peptide binding-unbinding process and the recognition mechanism of this peptide. In particular, we investigated the mechanistic details of each pathway to identify interactions that are important for the recognition of proteins and peptides. We analyzed the resulting free-energy profiles and observed that the final free-energy differences were 96.47 ± 12.63 kcal/mol for PW1, 96.1 ± 10.95 kcal/mol for PW2, 91.83 ± 9.81 kcal/mol for PW3, and 92 ± 11.32 kcal/mol for PW4. Consistent with the similar initial (bound) and final (unbound) states of the peptide in each pathway, the resulting free-energy differences (*ΔG*) are consistent among different pathways. However, the free-energy profiles for each pathway exhibited different magnitudes of the free-energy barriers for dissociation of the peptide, leading to the observation that PW4 is the preferred pathway of dissociation. In addition, the peptide dissociation was coupled with the formation of metastable states that resulted from a network of salt bridges formed between the arginine amino acids and the phosphate groups of the RNA backbone as well as from the hydrogen bonding. Specifically, we identified that the R8, R14, and R15 amino acids were important for the peptide recognition by the RRE RNA. Our results also suggest the R8 amino acid to be the most critical amino acid out of the three arginine amino acids because of its increased flexibility and the ability to form a primary or terminal salt-bridging interaction with the U72 nucleotide during the binding-unbinding process in PW4. These observations are potentially important for the recognition mechanism between the RNA molecules and the proteins and peptides that have charged amino acids. The simulation scripts used to generate data in this work are available in Appendices D and F of the doctoral thesis by Lev Levintov (117).

## Author contributions

L.L. and H.V. designed the research. L.L. performed the research and analyzed the data. L.L. and H.V. wrote the article.

## Supporting material

Document S1. Supporting materials and methods, Tables S1–S2, and Figs. S1–S16

Document S2. Article plus supporting material
