# Supplementary material for: Role of salt-bridging interactions in recognition of viral RNA by arginine-rich peptides
Source: Biophys J. 2021 Oct 26;120(22):5060–73. doi: 10.1016/j.bpj.2021.10.007 (PMC8633718; doi:10.1016/j.bpj.2021.10.007)
Supplement: Document S1. Supporting materials and methods, Tables S1–S2, and Figs. S1–S16 [file mmc1.pdf]

Biophysical Journal, Volume *120*

Supplemental information

Role of salt-bridging interactions in recognition of viral RNA by arginine-rich peptides

Lev Levintov and Harish Vashisth

Table of Contents

Supplemental Methods .... pg. S2
Supplemental Results .... pg. S3
Table S1 .... pg. S4
Table S2 .... pg. S5
Figure S1 .... pg. S6
Figure S2 .... pg. S7
Figure S3 .... pg. S7
Figure S4 .... pg. S8
Figure S5 .... pg. S9
Figure S6 .... pg. S10
Figure S7 .... pg. S11
Figure S8 .... pg. S12
Figure S9 .... pg. S12
Figure S10 .... pg. S13
Figure S11 .... pg. S14
Figure S12 .... pg. S15
Figure S13 .... pg. S16
Figure S14 .... pg. S17
Figure S15 .... pg. S18
Figure S16 .... pg. S19

SUPPLEMENTAL METHODS

Umbrella sampling simulations

We also computed the free-energy profile of peptide dissociation along PW4 using umbrella sampling (US) simulations to compare with the free-energy profile computed using cv-SMD simulations. In US simulations, the reaction coordinate is divided into a series of continuous windows in which a harmonic "umbrella" potential is added in addition to the force-field to bias the system and sample the conformational space limited to a given window. As the initial states for each window, we used the snapshots from the SMD simulation that required the least amount of work for peptide dissociation along PW4.
As a reaction coordinate, we used a distance collective variable which connected the C5 atom of the G47 nucleotide and the center of mass of the peptide residues Gly11 through Ala22. This collective variable was aligned with the vector of the pulling direction in PW4. The reaction coordinate was divided into 1 Å intervals from the initial binding pocket (r = 0 Å) to the unbound state (r = 50 Å). The bias potentials of each US window were set to 12 kcal $mol^{-1}Å^{-2}$ and the width of each window was set to 2 Å. We generated two independent data-sets of 50 windows each, where each window was sampled for 10 ns.
We maintained the temperature and pressure at 310 K and 1 atm using the Langevin thermostat and the Berendsen barostat, respectively. We applied restraints with k = 1000 kcal $mol^{-1}Å^{-2}$ to the phosphorous (P) atoms in the RNA backbone to prevent the overall rotation and translation of the RNA molecule. We also applied restraints to the atoms forming hydrogen bonds in the peptide residues Gly11 through Ala22 to maintain the secondary structure of the peptide during the dissociation. Based on two independent datasets, the free-energy profile was constructed using the weighted histogram analysis method (WHAM) software by the Grossfield lab (Fig. S13).[a]

Swarms-of-trajectories string method

We further used the swarms-of-trajectories string method (STSM) to test the reliability of the lowest free-energy pathway (PW4). The STSM is a path-finding algorithm that can refine the previously identified transition pathway by defining a string with a particular number of nodes or images from the original pathway which connect the initial and the final states. These images are then reparameterized in a high-dimensional space of defined collective variables, whose position is updated after each iteration. At first, a restrained simulation around the center of each image is conducted to generate representative conformations before allowing a small change in this center to occur in a series of free unrestrained simulation for the next iteration. The change in the center of each image is estimated by averaging over the drifts of a number of short unbiased trajectories which start from the conformations generated using the constrained simulations. Thus, the iteration consists of a series of simulations with and without restraints.
As the reaction coordinate, we used a distance collective variable which connected the C5 atom of the G47 nucleotide and the center of mass of the peptide residues Gly11 through Ala22. This collective variable was aligned with the vector of the pulling direction in PW4. As the initial states for 12 images in the string, we used 12 snapshots from the SMD simulation that required the least amount of work to be performed in PW4. We divided the reaction coordinate in 2 Å intervals from r = 0 Å to r = 22 Å. For each image, we conducted 10 MD simulations, each 100 ps long, in all 1000 ps simulation time per image per iteration. In total, we conducted 20 iterations per image. We maintained the temperature and pressure at 310 K and 1 atm using the Langevin thermostat and the Berendsen barostat, respectively. We applied restraints with k = 1000 kcal $mol^{-1}Å^{-2}$ to the phosphorous (P) atoms in the RNA backbone to prevent the overall rotation and translation of the RNA molecule.

PMF of the mutated RSG-1.2 peptide

To test the effect of mutations on the stability of the RSG-1.2 peptide, we conducted additional cv-SMD simulations by mutating each of the three key arginine residues (R8, R14, and R15) to an alanine residue. We energy minimized the system via the steepest descent minimization for 1000 steps. We then briefly equilibrated the volume of the simulation domain via a 500 ps long MD simulation in the NPT ensemble with a 2 fs timestep. We used the coordinates from the end of this MD simulation for subsequent cv-SMD simulations (10 simulations in total) in the NPT

[a] http://membrane.urmc.rochester.edu/?page_id=126

ensemble. The parameters and settings for these cv-SMD simulations were identical to the cv-SMD simulations conducted along the reaction coordinate for PW4. The resulting free energy profile is presented in Fig. S16.

SUPPLEMENTAL RESULTS

Effect of restraints and ensemble on the free energy profiles

In our work, we applied rotational restraints to prevent the peptide from rotating while it dissociates, which improved the convergence of the free-energy profile. At first, we conducted 350 cv-SMD simulations along each pathway without the application of rotational restraints. In Fig. S15A, we show the free-energy profiles along PW4 based on 350 cv-SMD simulations without the rotational restraints (red trace) and based on 75 cv-SMD simulations with the rotational restraints (blue trace). When rotational restraints were not applied, the free-energy profile did not converge (Fig. S15A). Additionally, we observed that the free-energy profile of simulations without rotational restraints resulted in a higher free-energy difference in comparison to the free-energy profile of simulations with rotational restraints. Thus, when rotational restraints were applied, the free-energy profile converged faster than without rotational restraints.

We also applied secondary structure restraints to prevent the peptide from unfolding during the cv-SMD simulations. If the secondary structure restraints were not applied, the peptide would unfold due to the pulling force experienced in cv-SMD simulations. Thus, the peptide unfolding would result from the unbinding forces and not from the natural forces of interaction with the RRE RNA or water molecules. We show this unphysical behavior in snapshots from a cv-SMD simulations along PW1 (Fig. S15B).

We further tested the effect of choice of ensemble on the free energy profile along PW4. Specifically, we conducted 30 cv-SMD simulations along PW4 in the NVT ensemble with the same set of parameters and settings as in the cv-SMD simulations with the NPT ensemble except for the ensemble settings. We present the resulting free-energy profile based on the NVT and NPT ensembles in Fig. S15C. We observed that the final free-energy difference between r = 0 Å and r = 50 Å in the NVT ensemble was 95.44 ± 13.04 kcal/mol which was comparable to the final free-energy difference in the NPT ensemble of 92 ± 11.32 kcal/mol (Fig. S15C). Thus, the final free-energy differences were similar and the effect of ensemble choice was minimal.

Table S1. Details on all simulation systems. The simulation details on all four pathways (PWs) are presented, including the dimensions of the simulation domain of each system (column labeled *system dimensions*), number of atoms (column labeled *system size*), pulling distance, simulation time of a single run, and the number of runs. The system size is slightly distinct in each pathway due to the initial reorientation of the RNA-peptide complex and resolvation.

PW	system dimensions	system size (atoms)	pulling distance (Å)	time/run (ns)	# runs
1	68 Å × 84 Å × 126 Å	63919	80	13	75
2	65 Å × 79 Å × 133 Å	61171	80	13	75
3	64 Å × 84 Å × 129 Å	61618	80	13	75
4	70 Å × 83 Å × 130 Å	66511	80	13	75

Table S2. Details on salt bridging interactions. The details on the atom of the amino acid (*Peptide*) and the atom of the nucleotide (*RNA*) that participate in salt bridging interactions are presented for each pathway (PW).

PW	Peptide	RNA
1	NH2/R15	O1P/U45
2	NH1/R8	O1P/G48
	NH2/R14	O2P/A68
	NH2/R15	O2P/U45
	NH1/R15	O1P/C44
3	NH2/R8	O2P/A68
	NH2/R14	O2P/A68
	NH2/R15	O2P/G42
4	NH1/R8	O1P/U72
	NH2/R14	O2P/A68
	NH1/R14	O1P/C69
	NH2/R15	O1P/C44

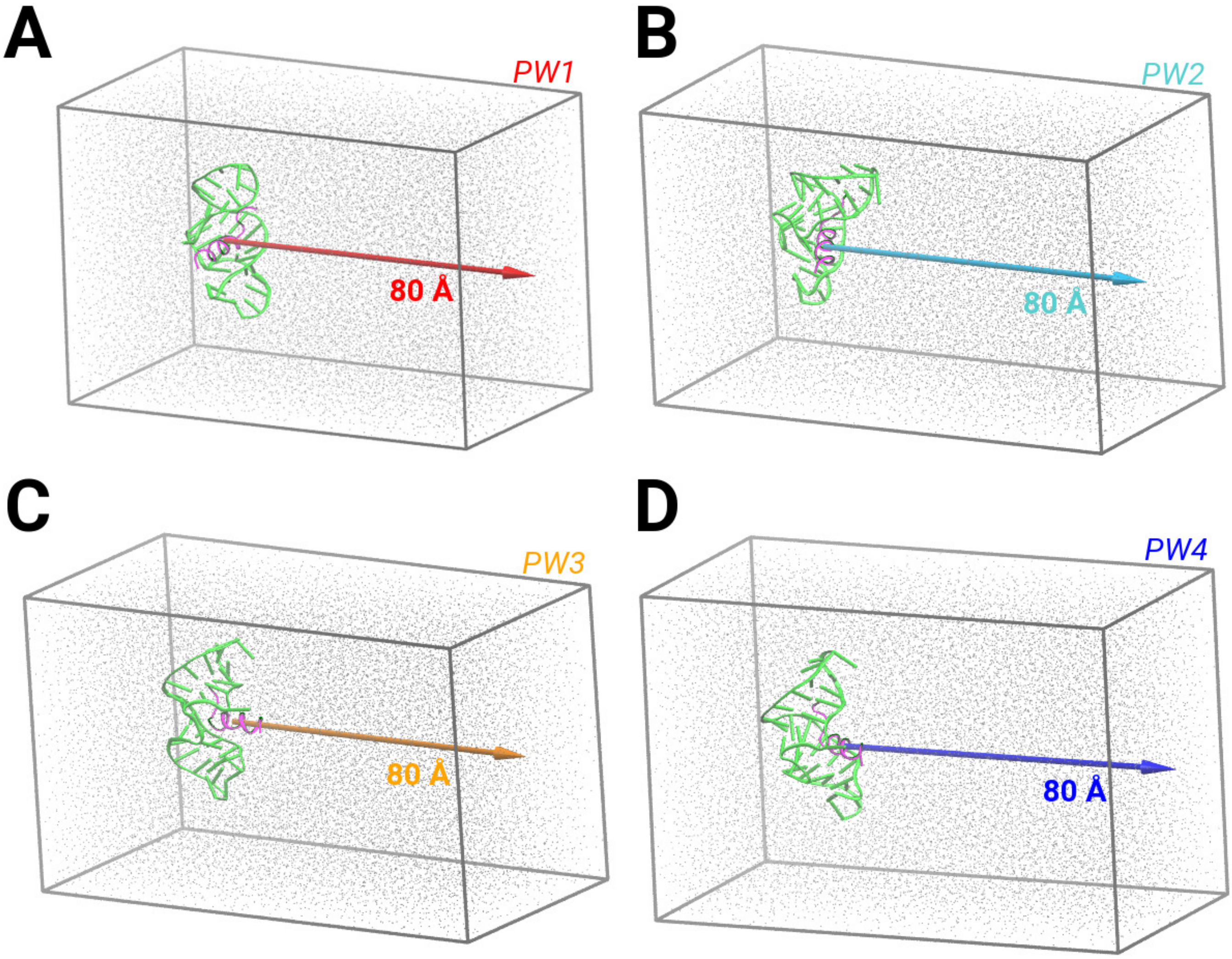

Figure S1. System setup: Shown are the side-views of the simulation domains along (A) pathway 1 (PW1), (B) pathway 2 (PW2), (C) pathway 3 (PW3), and (D) pathway 4 (PW4). In each snapshot, RNA is represented as a green cartoon; peptide as a purple cartoon; water molecules as gray points; and the bounding box in gray. The arrow in each panel indicates the reaction coordinate for each pathway that was used to conduct non-equilibrium cv-SMD simulations.

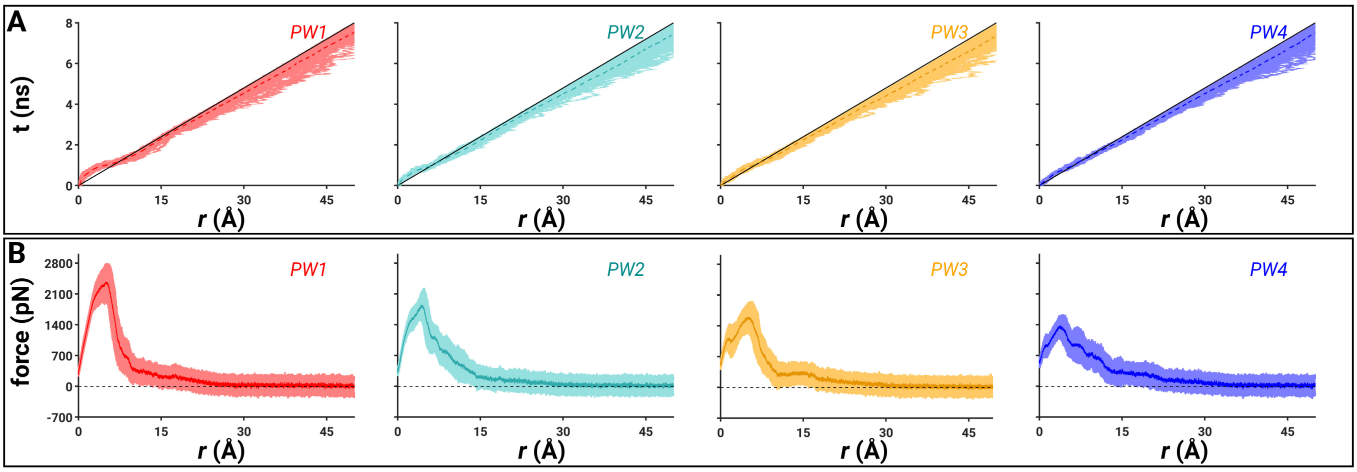

Figure S2. The reaction coordinates (*r*) and the unbinding force profiles (A) The center-of-mass (COM) trajectories of the peptide are shown in unique colors for PW1 (red), PW2 (cyan), PW3 (orange), and PW4(blue). The black solid lines represent the actual ***r***; the dark dotted lines represent the average trace across 75 trajectories for the corresponding PW; and the lighter shaded lines represent all SMD trajectories for the corresponding PW. (B) The unbinding force profiles are shown in unique colors for PW1 (red), PW2 (cyan), PW3 (orange), and PW4 (blue) with the average force traces (darker solid lines) and standard deviations (lighter shades).

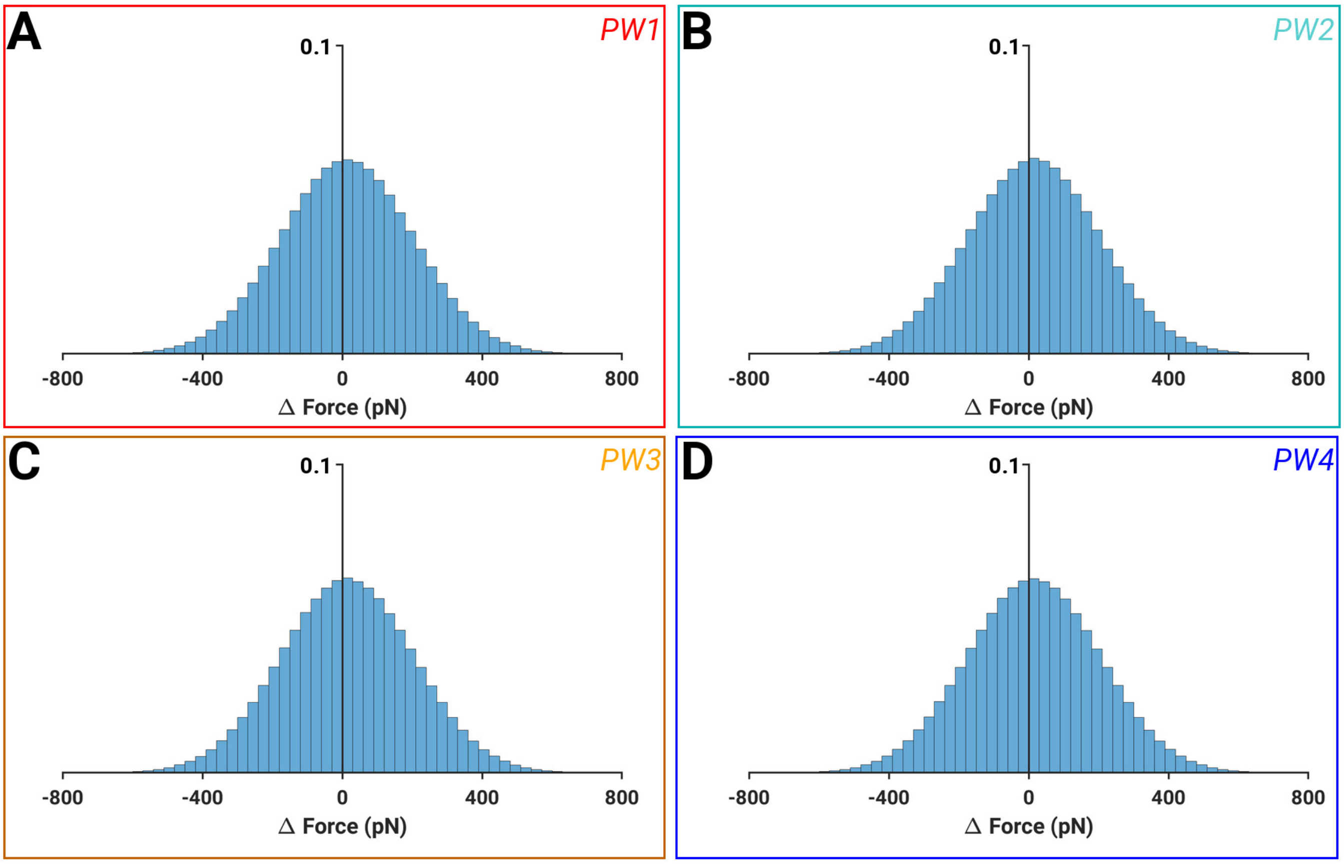

Figure S3. Force convergence data: Shown are the distributions of ΔForce values, defined as a difference between zero and the actual force value after the average force profile converged to zero for (A) PW1, (B) PW2, (C) PW3, and (D) PW4. The ΔForce values were measured after a distance of 40 Å along the reaction coordinate. See also Fig. S2B.

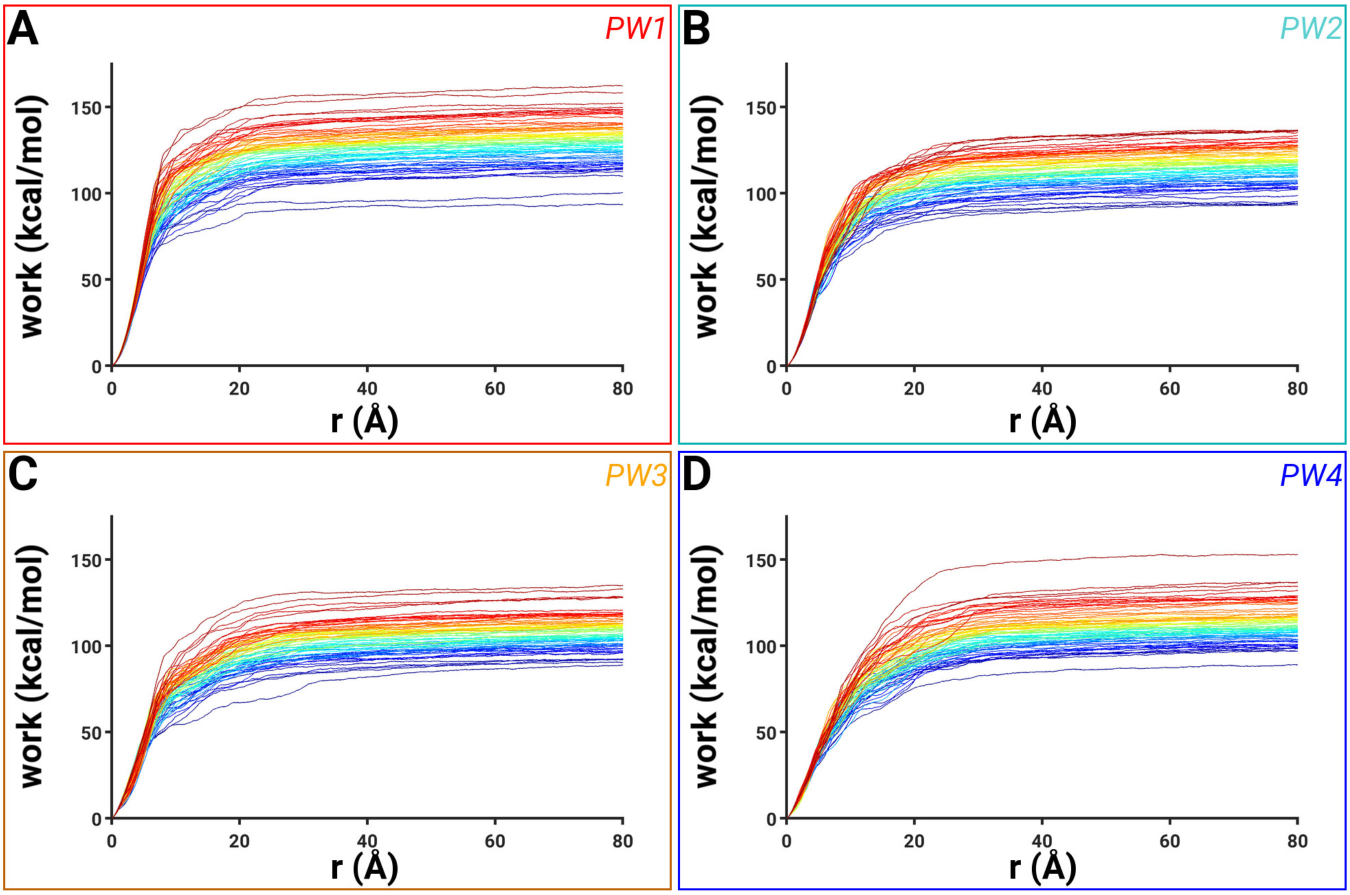

Figure S4. Non-equilibrium work profiles: The non-equilibrium work values obtained from 75 independent cv-SMD simulations for (A) PW1, (B) PW2, (C) PW3, and (D) PW4. The lower work values are indicated in blue traces and the higher work values in red traces.

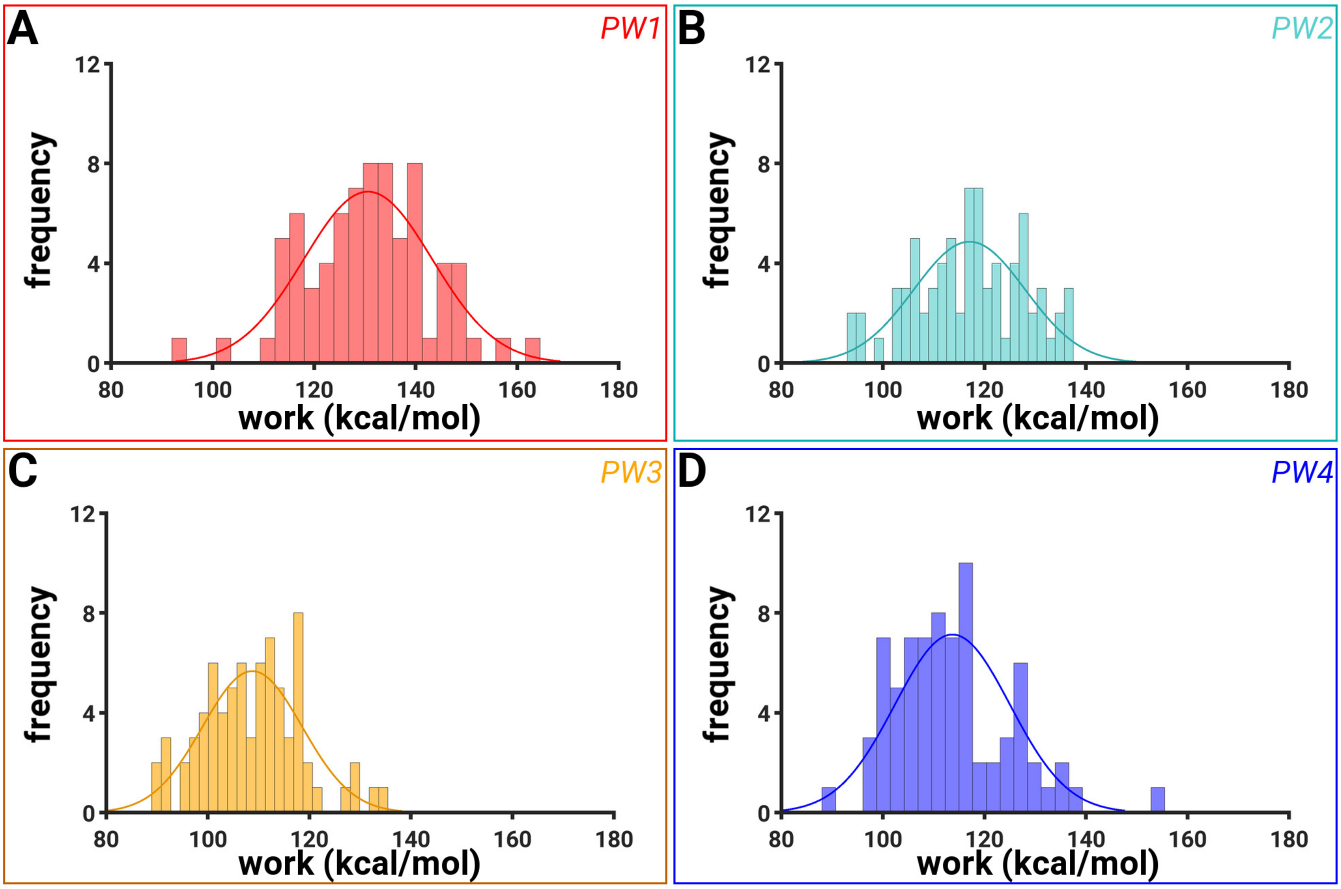

Figure S5. Distributions of the final work values: The histograms of all final work values during cv-SMD simulations are shown with a best-fit distribution line for (A) PW1, (B) PW2, (C) PW3, and (D) PW4.

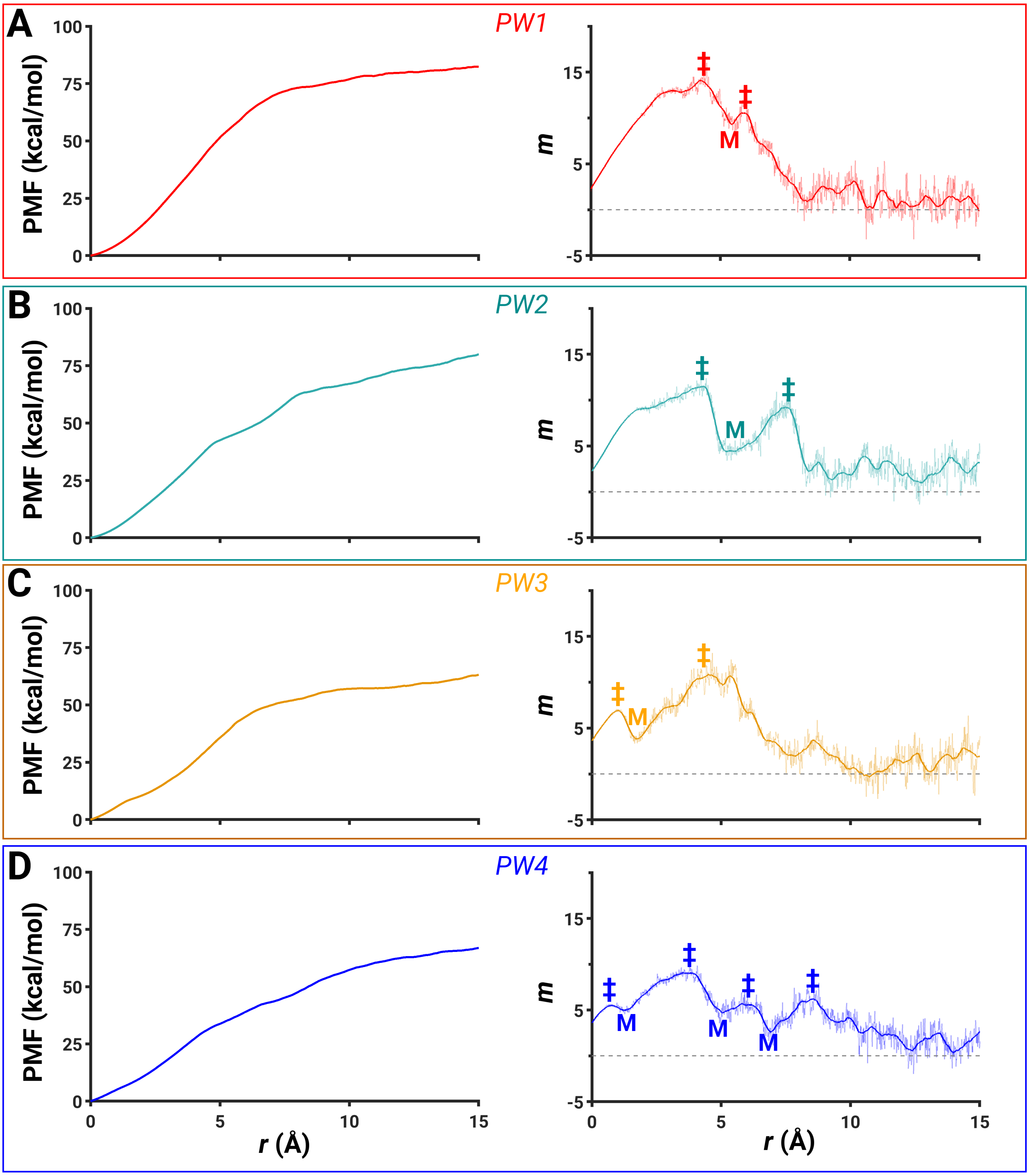

Figure S6. The free-energy and the corresponding first-order derivative profiles: A zoomed view of each free-energy profile (*left*) and the corresponding first-order derivative profile (*m*; *right*) computed every 100 points for r values between 0 Å and 15 Å are shown for (A) PW1, (B) PW2, (C) PW3, and (D) PW4. The fluctuations of the first-order derivatives are shown in light shaded colors. The free-energy barriers (indicated by ‡) and metastable states (indicated by M) are also shown and labeled.

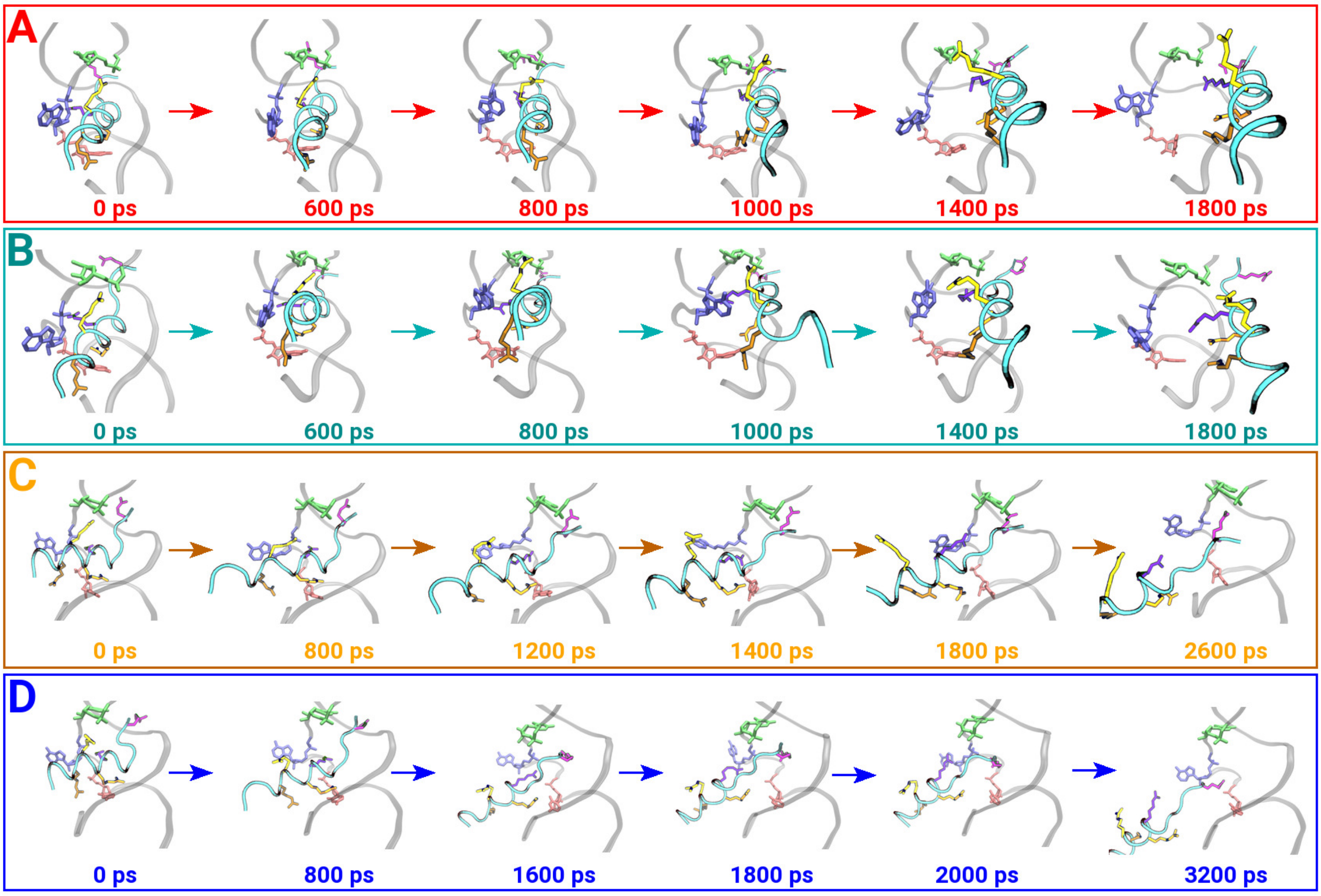

Figure S7. Dissociation pathways: Shown are the snapshots of the peptide (cyan tube with key amino acids highlighted) dissociating from the RRE RNA (gray cartoon) from the least work cv-SMD simulation for (A) PW1 (red), (B) PW2 (cyan), (C) PW3 (orange), and (D) PW4 (blue).

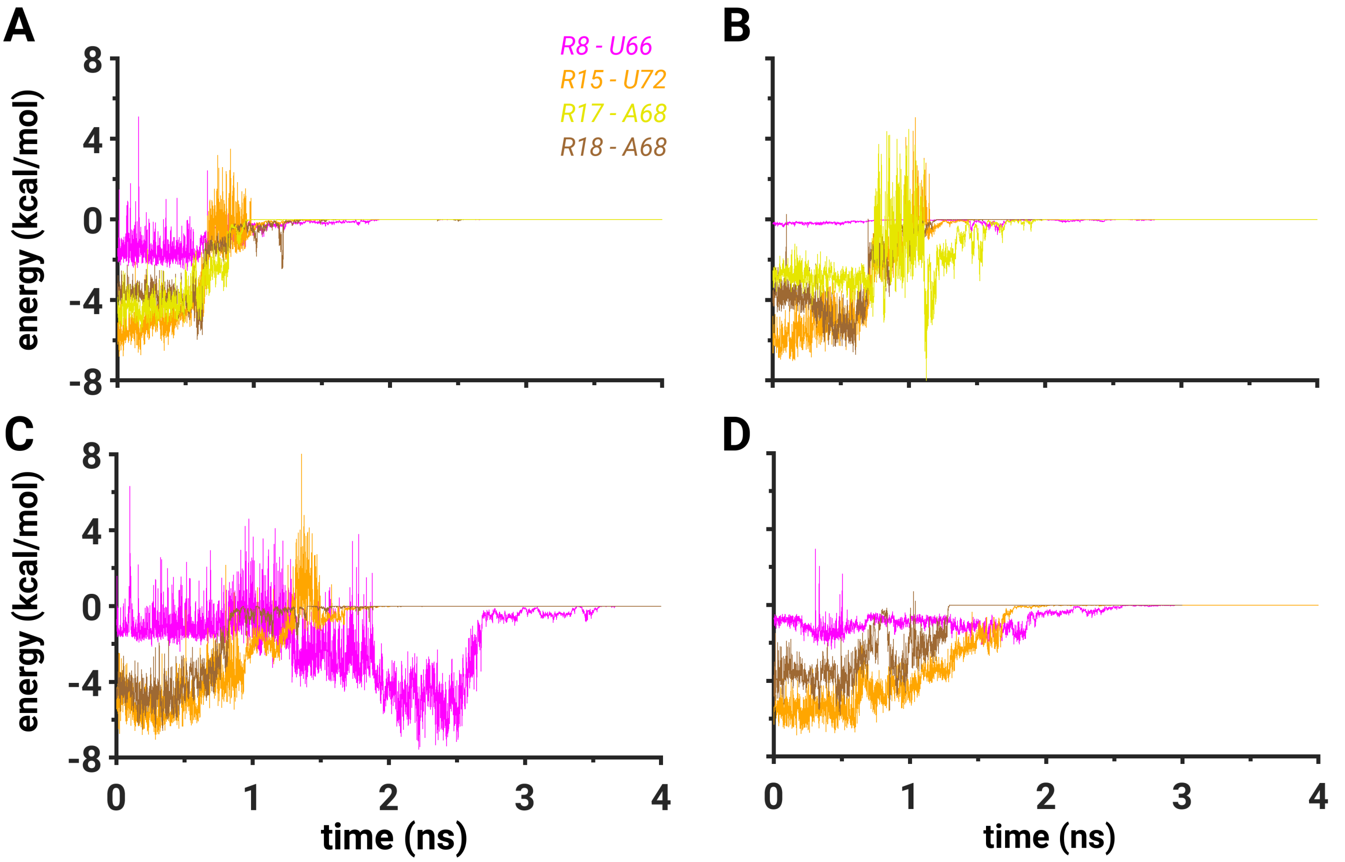

Figure S8. Van der Waals interaction energies: Shown are the time traces of the van der Waals interaction energy computed between the following amino acid - nucleotide pairs: the Arg8 (R8) amino acid and the U66 nucleotide (purple); the Arg15 (R15) amino acid and the U72 nucleotide (orange); the Arg17 (R17) amino acid and the A68 nucleotide (yellow); the Arg18 (R18) amino acid and the A68 nucleotide (brown). The energies were computed from the simulation with the least work in (A) PW1, (B) PW2, (C) PW3, and (D) PW4. Data after 4 ns are truncated due to the convergence to zero of each van der Waals energy trace.

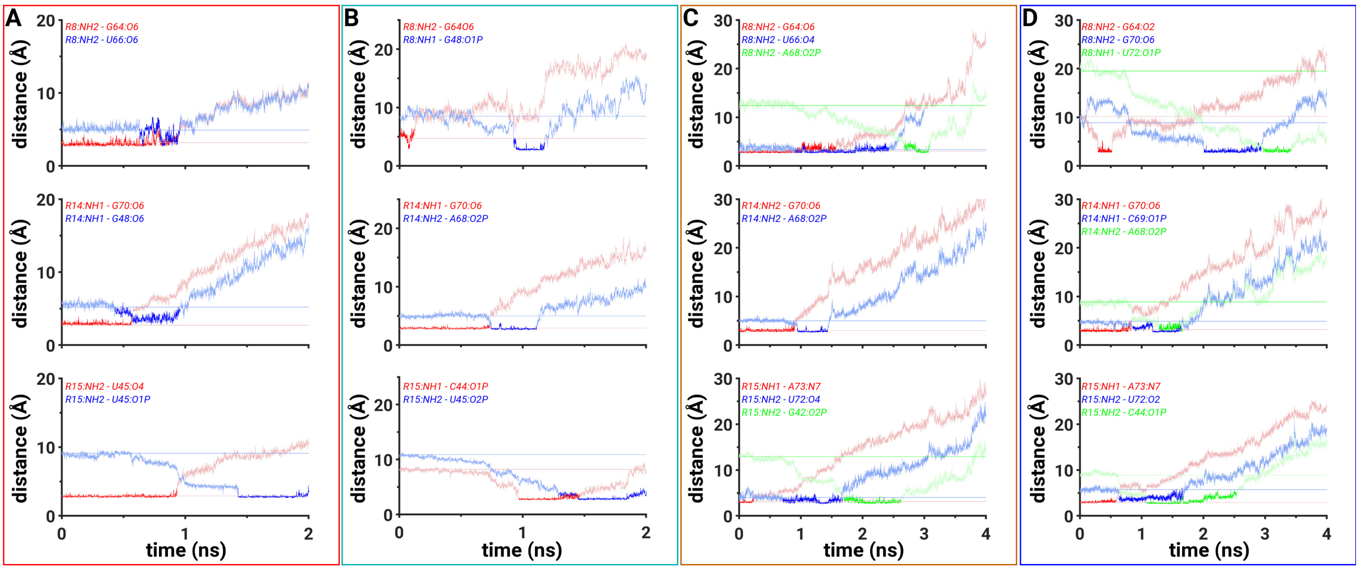

Figure S9. Key interatomic distance traces along each pathway: Shown are the traces of distances for each pathway (panel A, PW1; panel B, PW2; panel C, PW3; and panel D, PW4) corresponding to which data are presented in Figs. 3-6, where the y-axis scale is limited to 15 Å (Figs. 3 and 4) or 20 Å (Figs. 5 and 6).

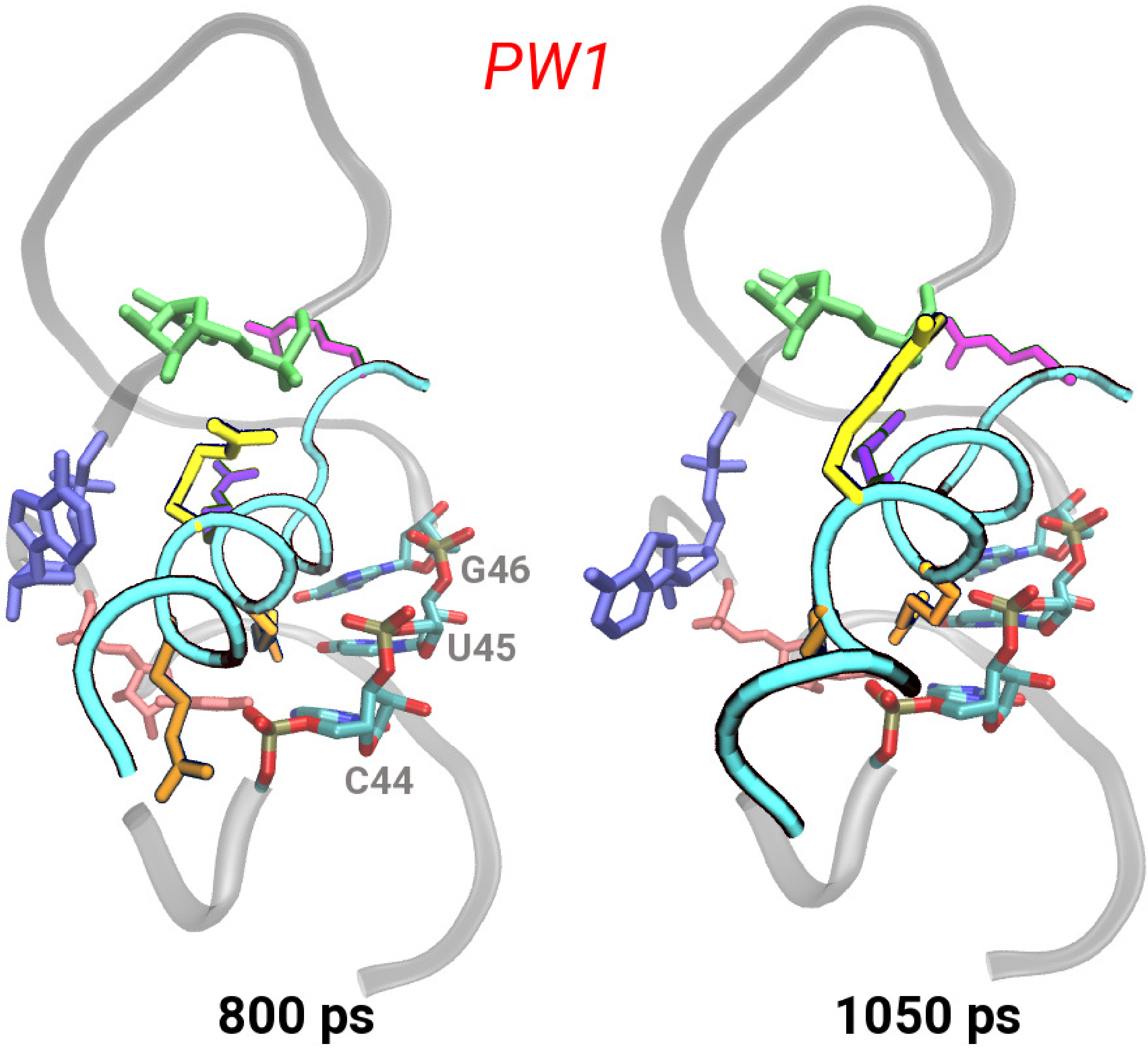

Figure S10. Snapshots from PW1: Shown are the snapshots of the peptide (cyan tube with key amino acids highlighted) dissociating from the RRE RNA (gray cartoon) from the lowest work simulation of PW1. Three nucleotides (U44, U45, and G46) which interact with the peptide through the atoms in the backbone are each shown in a stick representation. The color scheme is the same as in Fig. 1B.

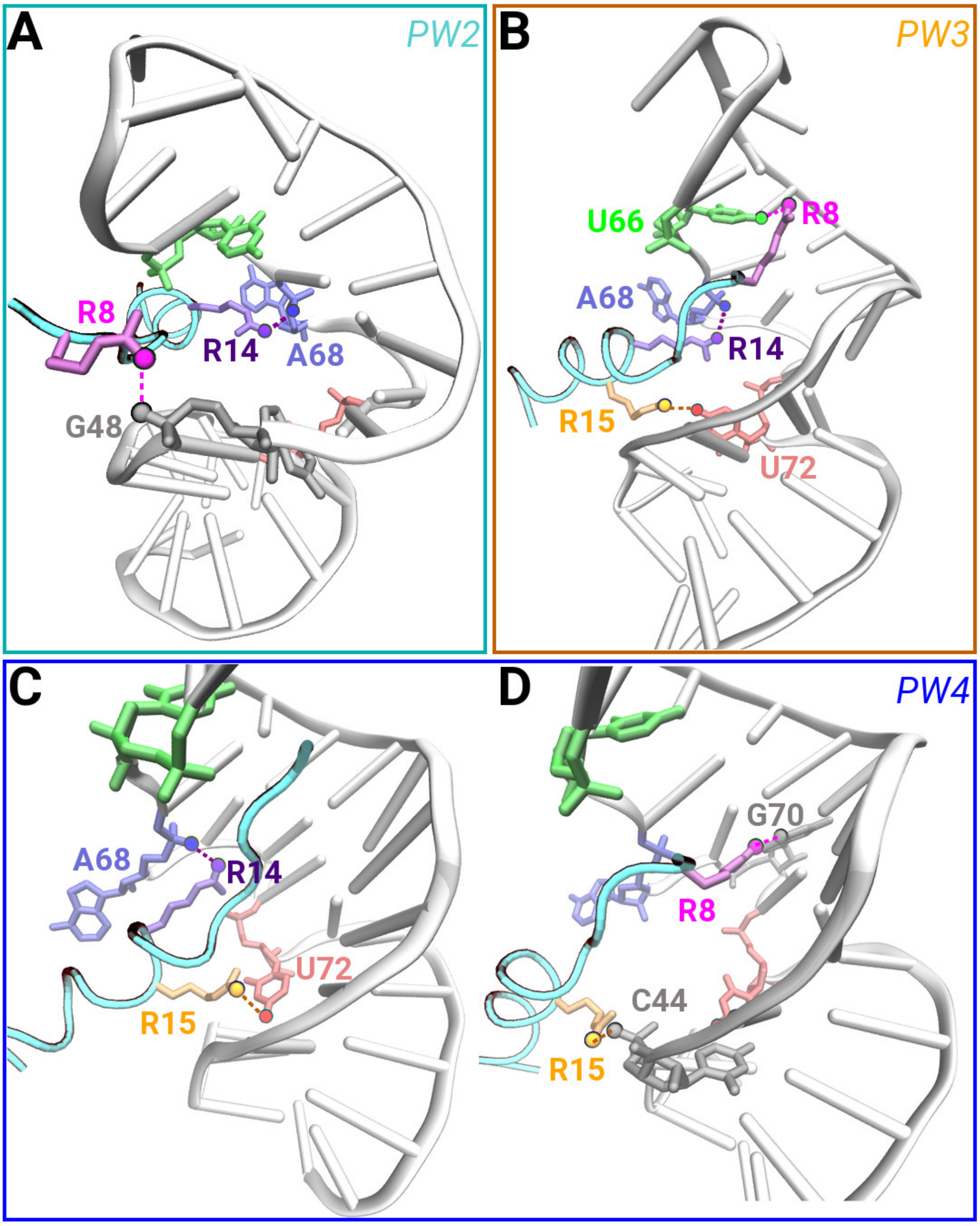

Figure S11. Networks of salt bridging and hydrogen bonding interactions: Shown are the snapshots of the peptide (cyan tube with key amino acids highlighted) dissociating from the RRE RNA (white cartoon) and forming a network of salt bridging and hydrogen bonding interactions from the least work cv-SMD simulations in (A) PW2, (B) PW3, and (C-D) PW4. Each amino acid, each nucleotide or an atom that participate in hydrogen bonding or salt bridging interactions (marked by a dashed line), are uniquely colored. The color scheme is the same as in Fig. 1B.

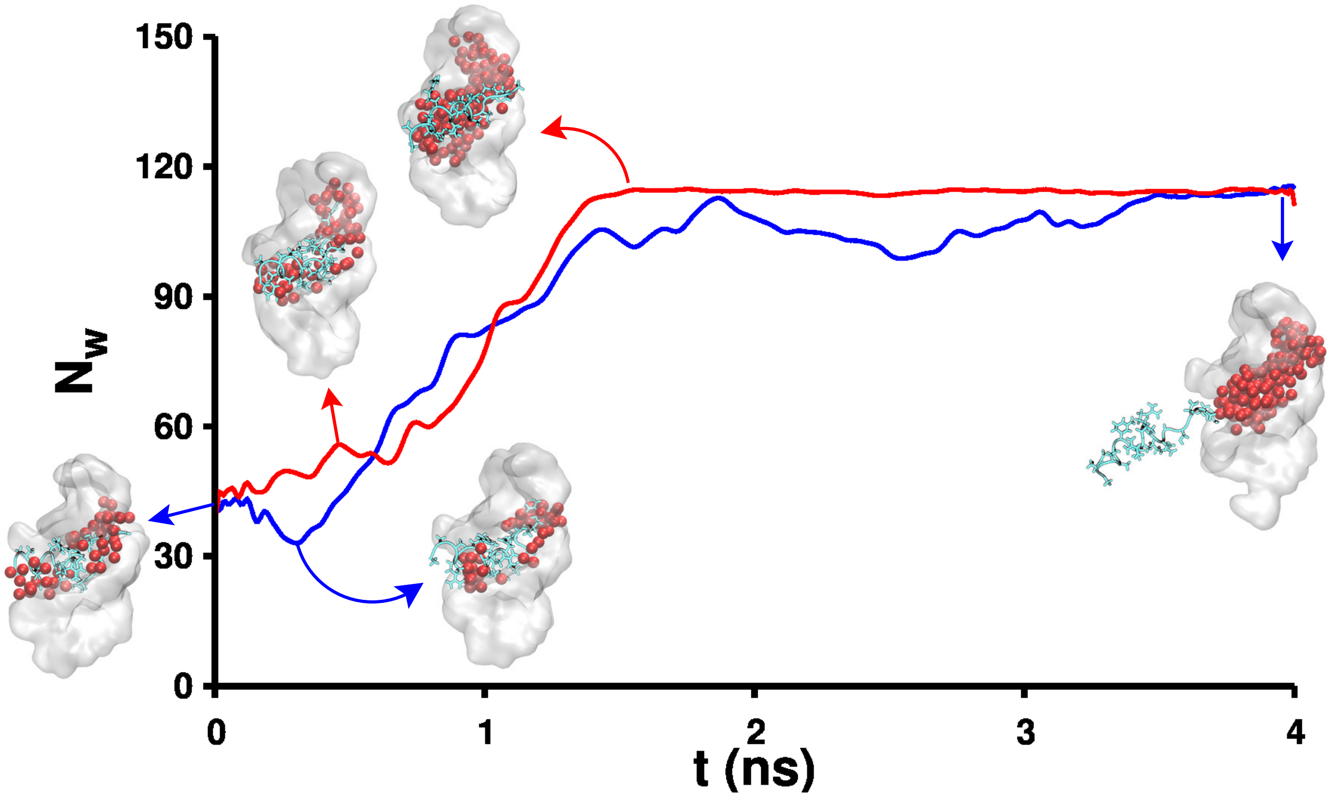

Figure S12. Solvation mechanism of the binding pocket: Shown are the time traces of the number of water molecules (N_w) solvating the binding pocket of the RRE RNA along PW1 (red) and PW4 (blue). The side-view snapshots are also shown of the solvation of the binding pocket at various time-points along each pathway. The RRE RNA is represented as a white surface, the peptide as a cyan cartoon, and the oxygen atoms of the water molecules as red spheres.

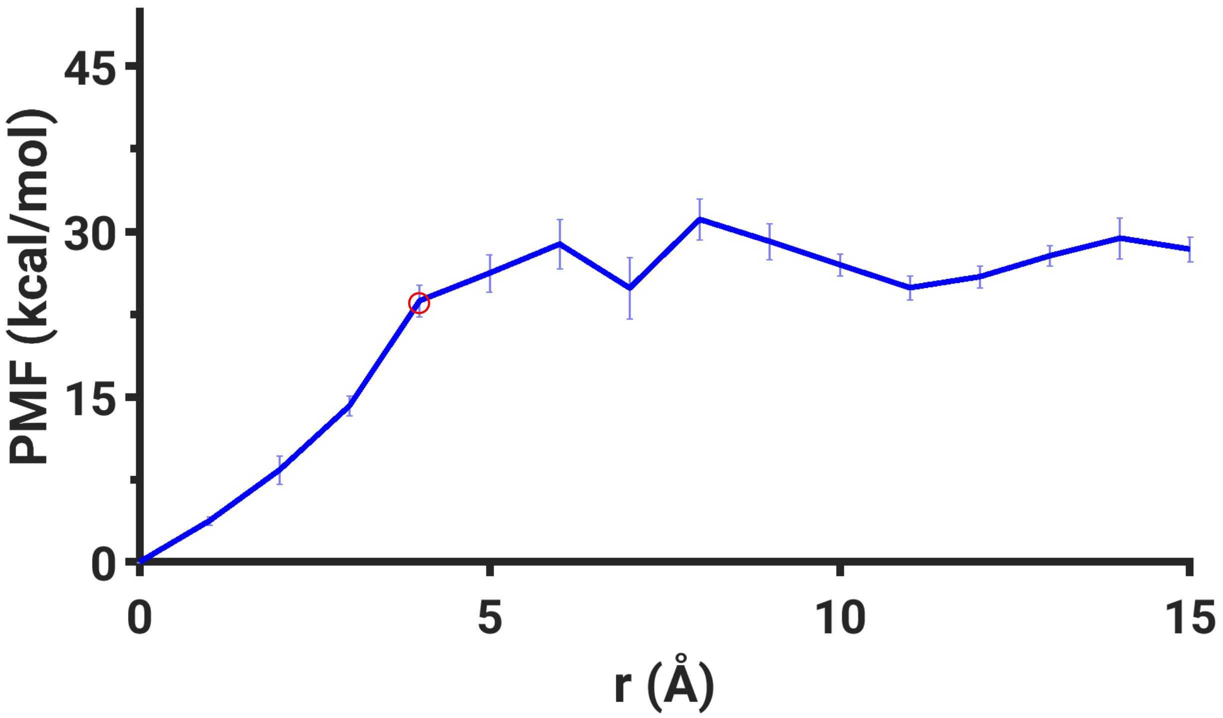

Figure S13. Umbrella sampling calculations for PW4. The free-energy profile along PW4 based on umbrella sampling simulations is shown along with the free-energy barrier (red circle) and the error bars (blue). The barrier calculated from the umbrella sampling calculations was 23.72±1.46 kcal/mol at ∼4 Å which is comparable to the free energy barrier of 24.46±2.08 kcal/mol at ∼3.8 Å that we have identified using cv-SMD simulations for PW4.

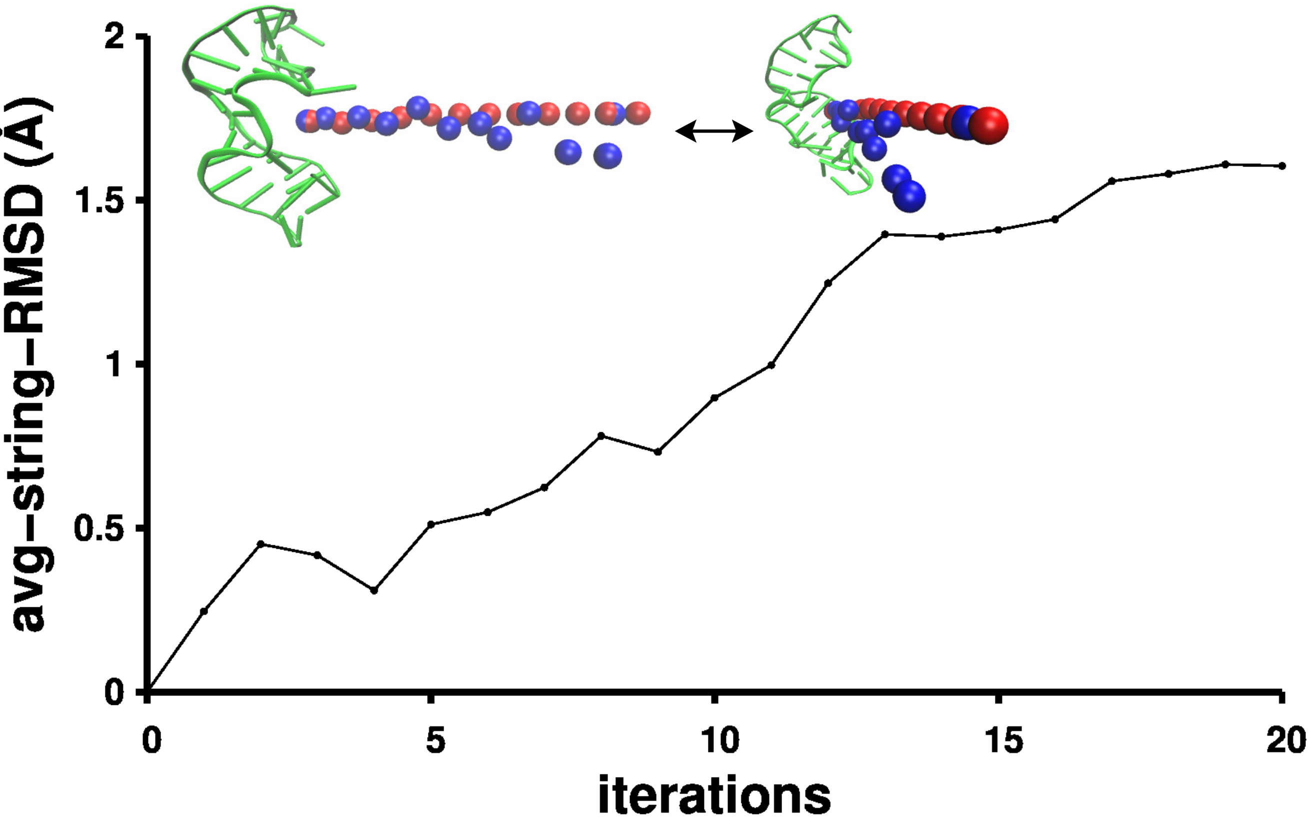

Figure S14. Convergence of the string. The average RMSD of the entire string for each iteration is shown for the application of the strong method to PW4. (*inset*) Shown are two side-view snapshots of the RNA (green cartoon) with the initial positions of the images on the string (red spheres) and the positions of the images at the final iteration (blue spheres). The cv-SMD pathway and the converged pathway from the string method only differ marginally in the bulk solvent.

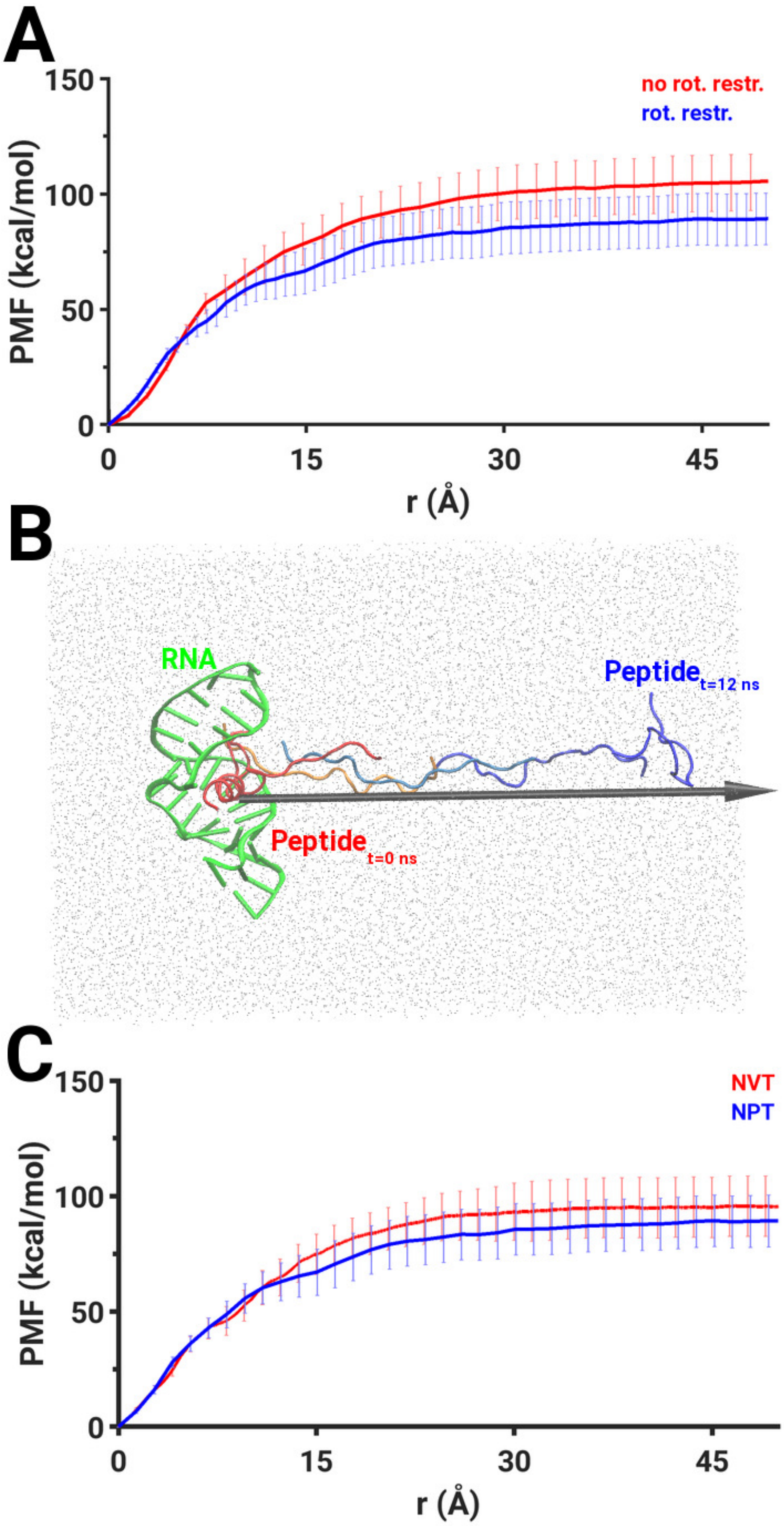

Figure S15. Effect of restraints and ensemble on PMF: (A) The free-energy profiles along PW4 are shown for a system without rotational restraints (red) and with rotational restraints (blue). (B) Shown are the side-view snapshots of dissociation of the peptide along PW1 in the absence of secondary structure restraints. The RNA is represented as a green cartoon and the peptide is shown in a cartoon representation at various time-points during the simulation (red, beginning; blue, end); water molecules are shown as gray points. The black arrow indicates the cv-SMD reaction coordinate. (C) The free-energy profiles along PW4 based on simulations conducted in the NVT (red) and NPT (blue) ensembles are shown.

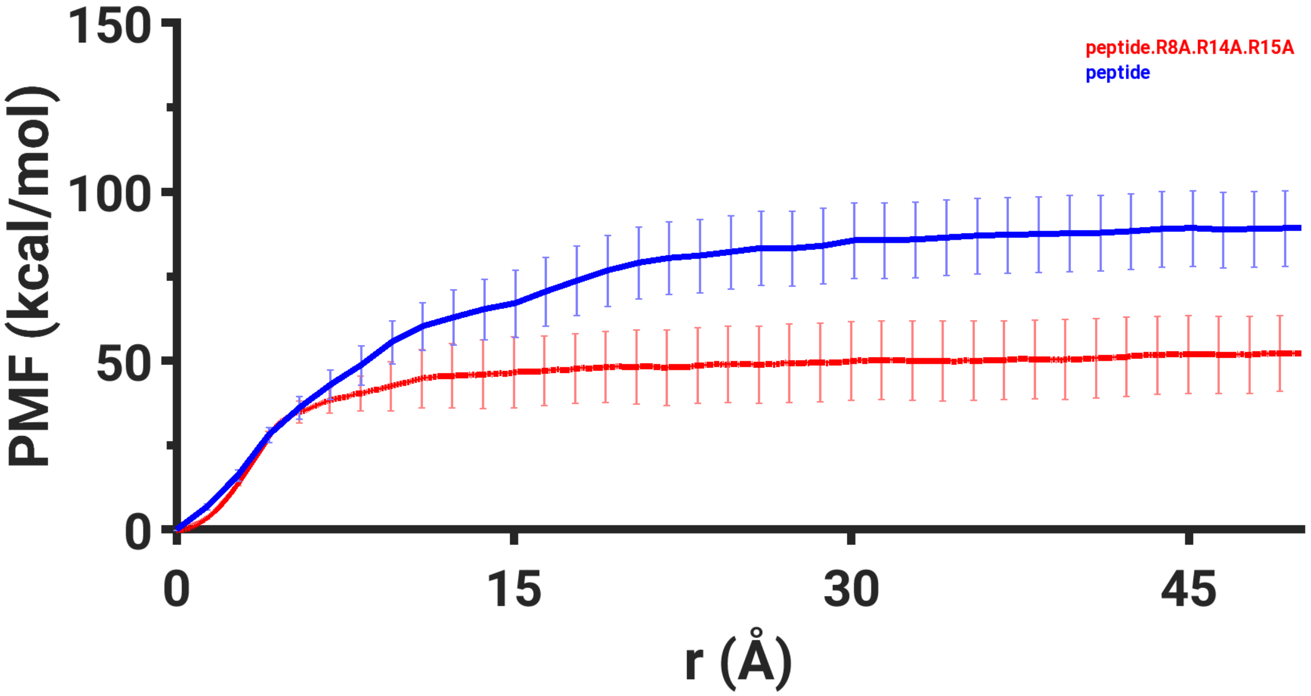

Figure S16. The free-energy profile of the mutated peptide: A comparison of the free-energy profiles is shown along PW4 for the RSG-1.2 peptide (blue trace) and its mutant peptide (red trace) with triple alanine mutations in three key arginine residue (R8, R14, and R15).
