# Supplementary material for: Role of salt-bridging interactions in recognition of viral RNA by arginine-rich peptides
Source: Biophys J. 2021 Oct 26;120(22):5060–73. doi: 10.1016/j.bpj.2021.10.007 (PMC8633718; doi:10.1016/j.bpj.2021.10.007)
Supplement: Document S2. Article plus supporting material [file mmc2.pdf]

Biophysical Journal
Article

Biophysical Society

Role of salt-bridging interactions in recognition of viral RNA by arginine-rich peptides

Lev Levintov[1] and Harish Vashisth[1,*]
[1]Department of Chemical Engineering, University of New Hampshire, Durham, New Hampshire

ABSTRACT Interactions between RNA molecules and proteins are critical to many cellular processes and are implicated in various diseases. The RNA-peptide complexes are good model systems to probe the recognition mechanism of RNA by proteins. In this work, we report studies on the binding-unbinding process of a helical peptide from a viral RNA element using nonequilibrium molecular dynamics simulations. We explored the existence of various dissociation pathways with distinct free-energy profiles that reveal metastable states and distinct barriers to peptide dissociation. We also report the free-energy differences for each of the four pathways to be 96.47 ± 12.63, 96.1 ± 10.95, 91.83 ± 9.81, and 92 ± 11.32 kcal/mol. Based on the free-energy analysis, we further propose the preferred pathway and the mechanism of peptide dissociation. The preferred pathway is characterized by the formation of sequential hydrogen-bonding and salt-bridging interactions between several key arginine amino acids and the viral RNA nucleotides. Specifically, we identified one arginine amino acid (R8) of the peptide to play a significant role in the recognition mechanism of the peptide by the viral RNA molecule.

SIGNIFICANCE We reveal key interactions that are involved in the recognition of a viral RNA by a peptide. Specifically, we discovered that the recognition of the peptide depends on the formation of salt bridges and hydrogen bonds that are formed between the arginine residues and the RNA backbone. We also demonstrated that these interactions formed a network of salt bridges that were spanning the major groove of RNA. These results enhance our understanding of the importance of arginine amino acids, or other basic amino acids, in the design of peptides that target viral RNA molecules.

INTRODUCTION

Numerous functions of RNA molecules depend on their interactions with proteins (1), which play a crucial role in various phases of the cell life cycle, including gene regulation (2,3), transcription (4,5), and translation (6). Consequently, misregulation of RNA-protein interactions can lead to neurological disorders, cardiovascular problems, and oncogenic diseases (7–10). Moreover, the interactions between viral RNA molecules and cellular or viral proteins are involved in the replication and transcription processes of various viruses, for example, human immunodeficiency virus (HIV), hepatitis C virus, and severe acute respiratory syndrome coronavirus (SARS CoV/CoV2) (11–14). Therefore, resolving the mechanistic details of RNA-protein interactions is essential for understanding various biological and biophysical processes (2–14).

Proteins and short peptides often interact with RNA molecules by adopting an α-helical or a β-sheet structure that can fit into the binding pocket of an RNA molecule (15–21) or through the interactions with the RNA backbone (1,22,23). Specifically, the RNA-peptide complexes are considered good model systems to study RNA-protein interactions and to probe the recognition mechanisms (24,25). A general RNA binding protein domain is the arginine-rich motif (ARM), which is found in ribosomal proteins (26), ribonucleoproteins (1,27), and viral proteins (11,28). The ARMs are short peptides that have a high concentration of arginine residues and have high affinity and specificity of interaction with their targets by adopting various conformations including α-helical, β-hairpin, or extended conformations (29). The interactions between these ARMs and RNA molecules have been investigated using NMR spectroscopy (11,15,30–34), circular dichroism spectroscopy (35,36), x-ray crystallography (37,38), and combinations of experimental and computational methods (29,39–41). Several comprehensive investigations have been conducted on the nucleic-acid-protein interfaces using structural and shape analyses to establish common features across known

Submitted March 31, 2021, and accepted for publication October 6, 2021.
*Correspondence: harish.vashisth@unh.edu
Editor: Jie Yan.
https://doi.org/10.1016/j.bpj.2021.10.007
© 2021 Biophysical Society.
This is an open access article under the CC BY license (http://creativecommons.org/licenses/by/4.0/).

complexes (42–45). Overall, these studies showed that the RNA-protein interactions are governed by sequence (e.g., composition of amino acids and nucleotides) or by shape (e.g., recognition of specific shapes of proteins).

However, the role of dynamics in RNA-protein interactions is still not fully understood because of challenges in capturing all the required parameters for describing a complex biomolecular system (23,46,47). Computational methods such as molecular dynamics (MD) simulations that are rooted in biophysical modeling are promising tools to enhance our knowledge of the recognition mechanism between RNA molecules and proteins by characterizing molecular motions at the atomic level (48). Although several RNA-protein complexes, for example, RNA-U1A complex (49–53) and other RNA recognition motifs (40,54–61), various double-stranded RNA-protein complexes (39,62–68), ribosomal RNA-protein complexes (69–72), and transfer RNA-protein complexes (73–79), have been investigated using MD simulations and free-energy methods, only a few studies have been conducted to investigate the interactions in viral RNA-protein complexes (80–86). Specifically, the studies on the viral RNA-protein complexes highlighted the importance of electrostatic interactions and the interactions between water molecules and proteins. However, most of these studies (80–84) were reported over a decade ago, and the force fields for nucleic acids and proteins have significantly improved in recent years (87). Additionally, the timescales of conventional MD simulations performed in these studies were limited. Thus, we still lack a full understanding of the viral RNA-protein recognition mechanisms and of specific interactions that need to be created or disrupted during the binding-unbinding process.

To address these questions, we applied nonequilibrium constant velocity steered MD (cv-SMD) simulations to study the binding-unbinding process of a helical arginine-rich peptide (RSG-1.2) from a conserved HIV-1 Rev response element (RRE) RNA segment, which is located in the *env* coding region and plays an essential role in viral replication (Fig. 1 *A*) (88). The RSG-1.2 peptide is a mutated Rev peptide with higher binding affinity and specificity in comparison with the canonical Rev peptide, which binds RRE RNA (88) and is a good model system for studying RNA-protein interactions (Fig. 1 *B*) (25).

Specifically, we conducted cv-SMD simulations along four distinct pathways (defined as PWs; Fig. 1 *C*; Fig. S1; Table S1). In these simulations, we observed the formation of specific interactions and the sequence in which those interactions were forming or rupturing during the dissociation process of the peptide along each pathway, which have not been reported previously. Based on our results, we propose the preferred pathway as well as the mechanism of recognition of the peptide. Additionally, we identified the role of arginine residues in recognition of the peptide by the RRE RNA. Based on our results, we suggest that the atomic scale details on the dissociation process and the recognition

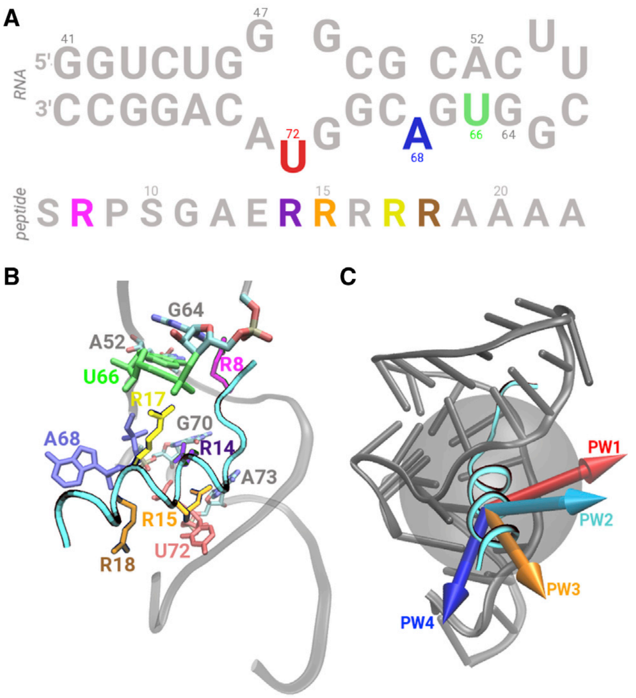

FIGURE 1 Structural details and system setup. (*A*) The sequences of the HIV-1 RRE RNA and the RSG-1.2 peptide are shown. The key nucleotides and amino acids are highlighted in unique colors. (*B*) A side-view of the binding pocket is shown in which the peptide is rendered as a cyan tube with the side chains of key residues highlighted in stick representations. Each key nucleotide in the RNA and each key amino acid in the peptide are highlighted in a unique color and labeled. (*C*) A side-view of the RRE RNA (*gray cartoon*) and the peptide (*cyan cartoon*) complex is shown. A transparent gray sphere represents the approximate volume of the peptide binding pocket. Each arrow corresponds to the peptide dissociation coordinate or direction for one of the four pathways (PWs): PW1 (*red*), PW2 (*cyan*), PW3 (*orange*), and PW4 (*blue*). To see this figure in color, go online.

mechanism of this peptide by the RRE RNA are potentially useful for designing new therapeutically relevant variants of this peptide.

MATERIALS AND METHODS

System setup and equilibration details

In this work, we have studied the (un)binding process of the RSG-1.2 helical peptide from the HIV-1 RRE RNA using steered MD (SMD) simulations along four different pathways (Fig. 1 *C*). We obtained the initial coordinates for our system from the first frame of the NMR structure deposited in the Protein Data Bank (PDB: 1G70) (33). We centered the RNA-peptide complex at the origin and rotated to align the dissociation direction of the peptide in each pathway along the same axis (Fig. S1). We then solvated each system in a periodic simulation domain of three-site transferrable intermolecular potential (TIP3P) water molecules (Fig. S1; Table S1). We neutralized the overall charge of the system with 27 Na^+ ions.

We energy minimized the system via the steepest descent minimization for 1000 steps that was followed by 500 cycles of conjugate-gradient minimization. To equilibrate the volume of the simulation domain, we conducted a 500-ps MD simulation in the NPT ensemble with a 2-fs timestep. We maintained the temperature and pressure at 310 K and 1 atm

using the Langevin thermostat and the Nosé-Hoover barostat in all MD and SMD simulations. We used periodic boundary conditions in all simulations and computed the electrostatic interactions using the particle mesh Ewald method. For the van der Waals interactions, we used a cutoff of 10 Å with switching initiated at 8 Å. We applied weak restraints to the phosphorous atoms in the RNA backbone to prevent the overall rotation and translation of the RNA molecule. We carried out all simulations using the NAnoscale MD (NAMD) (89) software package combined with the AMBER force field for RNA (RNA.ROC, the RNA force-field developed by the Rochester Group) (90) and for the peptide (ff14sb) (91). We used the TIP3P water model (92) for the solvent and the Li-Merz parameters for the ions (93). We analyzed all trajectories using the Visual Molecular Dynamics (VMD) and CPPTRAJ software (94,95).

Constant velocity cv-SMD simulations

To study the dissociation of the peptide along each of the four pathways, we performed cv-SMD simulations, referred hereafter also as SMD simulations. In cv-SMD simulations, a dummy atom is harmonically coupled to a group of atoms via a virtual spring that is pulled at a constant velocity along a specified direction (reaction coordinate), and the unbinding force is then measured. SMD simulations have been successfully applied to study unfolding of RNA-DNA (96,97) and unbinding mechanisms of protein-ligand (98,99) and RNA-ligand complexes (100–102) and to study other biophysical systems (103,104). We simulated only the unbinding process of the peptide because of the lack of knowledge of the initial configuration of the unbound RRE-RNA-peptide complex.

To select the four dissociation pathways, we considered a sphere that approximated the volume of the binding pocket (*gray sphere* in Fig. 1 *C*). We then selected points on the surface of the sphere that were radially separated by ~13 Å to prevent overlap with the RNA molecule. The arrows that are shown in Fig. 1 *C* indicate vectors passing through each of the defined points and represent unique reaction coordinates of dissociation along each of the four pathways. We used the coordinates from the end of the initial MD simulations for subsequent SMD simulations in the NPT ensemble. Specifically, for each of the four pathways, we conducted 75 SMD simulations, each of which was 13 ns long, thereby resulting in a total simulation time of 3900 ns. Our choice of 75 SMD simulations per pathway was based on the convergence of the free-energy profile along each pathway, indicating similar free-energy difference (ΔG) between the initial (bound) and the final (dissociated) states. We saved configurations every ps and the SMD output every 20 ps.

Consistent with the stiff-spring approximation (105), we applied a harmonic external force using a spring constant of $k = 12$ kcal/mol Å^2 that was attached to the center of mass of the peptide residues Gly11 through Ala22, which constitute the helical part of the peptide. We chose this part of the peptide to avoid deviations from the reaction coordinate that could be introduced by the movement of the unfolded segment (residues Ser7 through Ser10). After testing various values, we chose a pulling velocity of 0.00625 Å/ps, which is relatively slower than is commonly used in SMD simulations (0.015–0.02 Å/ps) (106–108). We also applied a harmonic restraint to prevent the rotation of the peptide to improve convergence of the free-energy profiles (see Supporting materials and methods, Results). As the reference orientation angle, we used the initial coordinates of the peptide, and we used a force constant of 3 kcal/mol deg^2 for the harmonic potential. We also applied restraints to the atoms forming hydrogen bonds in the peptide residues Gly11 through Ala22 to maintain the secondary structure of the peptide during the dissociation, which prevented the peptide unfolding that could occur in the absence of restraints (see Supporting materials and methods, Results). Although the binding mechanism of the peptide is not fully understood, it was suggested that the peptide is likely partially unfolded in the absence of RRE RNA (33,109). However, in our work, we have focused explicitly on the unbinding process of the peptide while maintaining its secondary structure because resolving both folding and binding processes simultaneously is a challenging task. We also verified that there was no bias introduced by the choice of the ensemble in SMD simulations (see Supporting materials and methods, Results).

Potential of mean force calculation

We computed the potential of mean force (PMF) as a function of the distance along the reaction coordinate (r), which increases at a constant velocity v such that $\lambda_t = \lambda_0 + vt$, where $\lambda_0 = 0$ initially. The r vector is commensurate with the pulling direction. The external work performed in a nonequilibrium SMD trajectory is then given by the following equation:

$$W_{0\rightarrow t} = -kv\int_0^t (r - (\lambda_0 + vt))dt.$$

According to the protocol developed by Jensen et al. (110), we used the exponential averaging of the Jarzynski's equality (111) to estimate the PMF along the reaction coordinate from work distributions obtained using SMD simulations (105,112). We have previously demonstrated the utility of this approach for studying RNA-ligand interactions (102). The exponential averaging expression is as follows:

$$\Delta G = -\beta^{-1} ln\overline{\exp(-\beta W)},$$

where $\beta = 1/k_B T$ with k_B Boltzmann's constant and T the temperature, W is the nonequilibrium work performed, and ΔG is the equilibrium free-energy difference.

Interaction energies and salt bridges

We also computed the nonbonded interaction energies between a specific amino acid of the peptide and a specific nucleotide of the RRE RNA. In particular, we calculated the van der Waals energy between all atoms in the following pairs of amino acids and nucleotides: Arg8 or R8 and U66; Arg15 or R15 and U72; Arg17 or R17 and A68; and Arg18 or R18 and A68.

We also analyzed a network of hydrogen-bonding and salt-bridging interactions formed between a specific arginine amino acid and a specific RNA nucleotide. Hydrogen bonds were defined between a hydrogen atom of the arginine amino acid and a heavy atom (oxygen or nitrogen atom) of the RNA nucleotide. Salt bridges were defined between a nitrogen atom of the arginine amino acid and the oxygen atom of the phosphate group in the RNA backbone. The definition and the cutoff value of 3.5 Å for hydrogen-bonding and salt-bridging interactions were adopted from a previous study (83). Specifically, we computed the salt bridge distances between the atoms presented in Table S2.

Solvation of the binding pocket

We also characterized the solvation of the binding pocket during dissociation of the peptide from the RRE RNA. We defined the binding pocket to be comprised of 15 nucleotides (U43, C44, U45, G46, G47, G48, C49, G50, C51, G67, A68, C69, G70, G71, and U72) and computed the number of water molecules that are confined within the volume of the binding pocket formed by these nucleotides.

RESULTS

Thermodynamics of peptide dissociation

Using nonequilibrium cv-SMD simulations, we studied the dissociation of the RSG-1.2 peptide from the RRE RNA along four distinct pathways (Fig. 1 *C*). During these

SMD simulations, the peptide consistently followed the reaction coordinate (Fig. S2 *A*). We also calculated the unbinding force profiles to ascertain that the average force converged to zero, corresponding to a fully dissociated state of the peptide and with no residual interactions with the RNA. In Fig. S2 *B*, we show the average force profiles with error bars for each pathway, which highlight that the average force for the dissociation of the peptide converged to zero after ∼35–40 Å, depending on the pathway. The convergence to zero is further ascertained by computing the distributions of force values after 40 Å for each pathway that reveal a mean of zero (Fig. S3). Then, we computed the nonequilibrium work required for the dissociation of the peptide from each of the 75 simulations for all four pathways (Figs. S4 and S5). The resulting work distributions were used to estimate the free-energy or PMF profile along the reaction coordinate for each pathway (Fig. 2 *B*) using the Jarzynski's equality (111), which relates the nonequilibrium work to the equilibrium free-energy difference (ΔG). Because nonequilibrium trajectories with the least work have the highest contribution to the equilibrium free-energy difference estimated using the Jarzynski's equality, we provide mechanistic details from these trajectories.

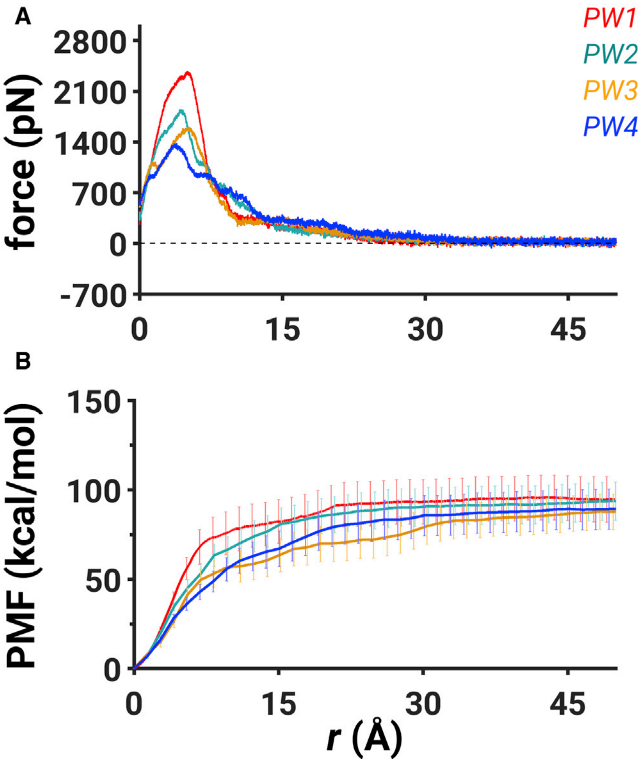

FIGURE 2 The unbinding force and the free-energy profiles. (*A*) The traces of the averaged unbinding force along each pathway are shown: PW1 (*red*), PW2 (*cyan*), PW3 (*orange*), and PW4 (*blue*). (*B*) The free-energy profile with standard deviations shown as error bars along each pathway is shown. See also Figs. S2 and S6. To see this figure in color, go online.

Unbinding force profiles

The intermediate steps of the peptide dissociation in each pathway are quantitatively described using the unbinding force profiles (Fig. 2 *A*). At the beginning of each SMD simulation ($r = 0$ Å), the peptide was located in the bound state, interacting with the RNA nucleotides in the binding pocket (Fig. 1 *B*). In particular, the R8 amino acid was initially interacting with the U66, G64, and A52 nucleotides; the R14 amino acid was interacting with the G70 nucleotide; R15 was interacting with the A73 and U72 nucleotides; and the R17 amino acid was initially interacting with the A68 nucleotide (Fig. 1 *B*). A gradual increase in the external force values for each pathway (Fig. 2 *A*) indicates that the peptide began to dissociate from the binding pocket by overcoming the interactions with the binding pocket nucleotides. The peak force values correspond to the stage when the peptide has moved out of the binding pocket by rupturing key interactions with the RNA. The external force values then decreased as the peptide was at a distance of ∼35–40 Å when the force values on average converged to zero, signifying that the peptide reached the dissociated state (Fig. 2 *A*).

We further analyzed the unbinding force profiles that exhibited different magnitudes of the maximal force of dissociation in each pathway. Specifically, we observed that PW1 had the highest value of the maximal force (∼2377 pN) of dissociation occurring at ∼5 Å (*PW1* in Fig. 2 *A*). The force profile in PW2 exhibited the second highest value of the maximal force (∼1850 pN) of dissociation at ∼4.5 Å (*PW2* in Fig. 2 *A*). Additionally, we detected a smaller peak of the unbinding force (∼1090 pN) at ∼7.2 Å in PW2. We observed that PW3 exhibited the third highest value of the maximal force (∼1600 pN) of dissociation at ∼5.2 Å (*PW3* in Fig. 2 *A*). Moreover, we detected a smaller force peak value at 1.3 Å in PW3 corresponding to ∼1120 pN. Finally, we observed the lowest value of the maximal force (∼1360 pN) of dissociation in PW4, which occurred at ∼3.8 Å (*PW4* in Fig. 2 *A*). We also located smaller peaks in force at ∼1.2 Å and at ∼6.6 Å, which were both equal to ∼950 pN. We detected a variability in the location of the maximal force value in the individual trajectories. The maximal force values were located between 4.6 and 5.4 Å in PW1, between 4.3 and 4.8 Å in PW2, between 4.9 and 5.4 Å in PW3, and between 3.6 and 4.5 Å in PW4 (Fig. S2 B). We observed that the unbinding force profiles converged to zero at 35 Å for PW1 and PW2 and at 40 Å for PW3 and PW4 (Fig. 2 *A*; Fig. S2 *B*).

Free-energy profiles

We report the free-energy profiles for each pathway (Fig. 2 *B*) that provide additional information on the thermodynamics of peptide dissociation, including the free-energy barriers and the metastable states. All reported free-energy

values are measured with respect to the initial state. We also show a zoomed view of each free-energy profile for *r*-values between 0 and 15 Å along with the first-order derivative (*m*) of the free-energy profile computed for the same range of *r*-values in each pathway (Fig. S6). The first-order derivative provides information on the rate of change of the free-energy profile and assists in identifying metastable states (*labeled M*, Fig. S6) as well as the free-energy barriers (*labeled double dagger*, Fig. S6). The first-order derivative converges to zero, corresponding to the region of the free-energy or the PMF profile when there is no significant change in the PMF.

We observed that the highest free-energy barrier of dissociation was in PW1, which was equal to 41 $\pm$ 3.67 kcal/mol at ~4.2 Å with an additional free-energy barrier of 61.67 $\pm$ 7.41 kcal/mol at 6 Å (*red double dagger*, Fig. S6 *A*). We observed the second highest free-energy barrier in PW2 corresponding to 37.51 $\pm$ 2.62 kcal/mol at ~4.4 Å with an additional free-energy barrier of 58.08 $\pm$ 5.96 kcal/mol at ~7.5 Å (*cyan double dagger*, Fig. S6 *B*). In PW3, we observed several free-energy barriers at ~1 Å and at ~4.6 Å, corresponding to the free-energy values of 4.49 $\pm$ 0.18 kcal/mol and 31.47 $\pm$ 3.77 kcal/mol, respectively (*orange double dagger*, Fig. S6 *C*). Finally, in PW4, we observed four free-energy barriers at ~0.8, ~3.8, ~5.9, and ~8.5 Å, corresponding to the free-energy values of 3.66 $\pm$ 0.34, 24.46 $\pm$ 2.08, 38.46 $\pm$ 3.59, and 50.48 $\pm$ 5.99 kcal/mol (*blue double dagger*, Fig. S6 *D*).

We also observed the formation of metastable states along different pathways (*labeled M* in Fig. S6). We located the metastable states at ~5.4 Å in PW1 (*red M*, Fig. S6 *A*), ~5.4 Å in PW2 (*cyan M*, Fig. S6 *B*), 1.8 Å in PW3 (*orange M*, Fig. S6 *C*), and 1.3, 5.1, and 6.9 Å in PW4 (*blue M*, Fig. S6 *D*). The mechanistic details of each metastable state are provided in the following section. Finally, we observed that the free-energy differences between the initial states (r = 0 Å) and the dissociated states (r = 50 Å) were 96.47 $\pm$ 12.63 kcal/mol for PW1, 96.1 $\pm$ 10.95 kcal/mol for PW2, 91.83 $\pm$ 9.81 kcal/mol for PW3, and 92 $\pm$ 11.32 kcal/mol for PW4. Thus, the resulting free-energy differences (ΔG) have similar values, falling within the range of error bars for each pathway. Overall, we observed that PW4 has the smallest free-energy barrier for dissociation of the peptide while having additional metastable states in comparison with other pathways.

Mechanistic details of peptide dissociation pathways

In the initial conformation, the peptide is bound in the major groove of the RRE RNA between the A75-U45 and U66-A52 basepairs while largely maintaining an α-helical conformation with five residues constituting a coiled segment at the N-terminus (Fig. 1 *B*) (33). The A68 and U72 nucleotides were in the flipped-out configurations, recognizing the peptide through stacking interactions with the R15 and R18 amino acids, respectively (Fig. 1 *B*). The Hoogsteen edge of the G70 and A73 nucleotides formed hydrogen-bonding interactions with the R14 and R15 amino acids, respectively. The R8 amino acid from the coiled segment of the peptide interacts with the U66 nucleotide, whereas the R17 and R18 amino acids also form contacts with the RNA backbone.

Pathway 1

During the early part of the lowest-work SMD simulation in PW1, the peptide began dissociating out of the binding pocket (Fig. S7 *A*), which was also characterized by weakening of interactions between several key amino acids and nucleotides (Fig. S8 *A*). In particular, we observed that the van der Waals interaction energy between the R8 amino acid and the U66 nucleotide, the R15 amino acid and the U72 nucleotide, and the R17 amino acid and the A68 nucleotide approached zero (Fig. S8 *A*), indicating negligible interactions between the residues. Specifically, at t = ~0.6 ns, the hydrogen bond between the NH2 atom of R8 amino acid and the O6 atom of G64 weakened (*red trace*, Fig. 3 *A*), and a new hydrogen bond was formed between the NH2 atom of R8 amino acid and the O6 atom of U66 (*blue trace*, Fig. 3 *A*). Additionally, at t = ~0.6 ns, the hydrogen bond between the NH1 atom of R14 amino acid and the O6 atom of G70 broke (*red trace*, Fig. 3 *B*), which led to the formation of a hydrogen bond between the NH1 atom of R14 amino acid and the O6 atom of G48 (*blue trace*, Fig. 3 *B*). This sequence of events was a result of the peptide leaving the initial binding pocket, which was coupled with the formation of new hydrogen-bonding interactions between the R8 and R14 amino acids and the U66 and G48 nucleotides, respectively (Fig. 3, *A and B*).

At t = ~1 ns, the peptide was located in the proximity of the backbone atoms of the C44, U45, and G46 nucleotides that constitute the major groove of the RNA (Fig. S10) and the van der Waals interactions between the R8, R15, and R17 amino acids, and the U66, U72, and A68 nucleotides diminished (Fig. S8 *A*). This was also characterized by the rupture of the hydrogen bonds that were previously formed at t = ~0.6 ns between the NH2 atom of R8 amino acid and the O6 atom of U66 and between the NH1 atom of R14 amino acid and the O6 atom of G48 (*blue trace*, Fig. 3, *A and B*). The state when the peptide was located in the proximity of the backbone atoms of the C44, U45, and G46 nucleotides corresponds to a weak metastable state in the free-energy profile (*red M*, Fig. S6 *A*).

At t = ~1.4 ns, the peptide displaced the backbone atoms of the C44, U45, and G46 nucleotides and was located in a partially dissociated state, whereas the R8, R14, and R15 amino acids were still in the vicinity of the RRE RNA

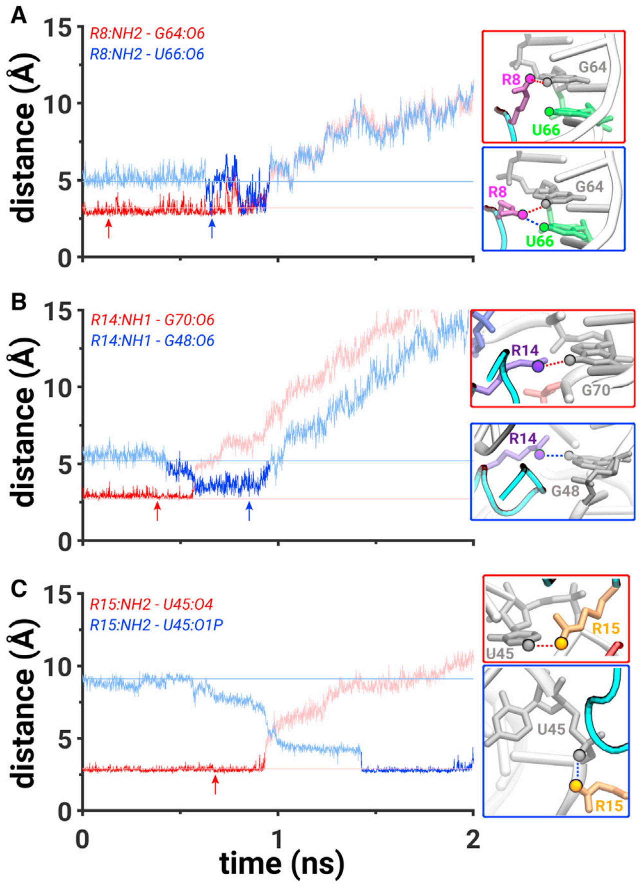

FIGURE 3 Mechanistic details of PW1. (*A*) The hydrogen bond distances between the NH2 atom of R8 and the O6 atom of G64 (*red trace*) and between the NH2 atom of R8 and the O6 atom of U66 (*blue trace*). (*B*) The hydrogen bond distances between the NH1 atom of R14 and the O6 atom of G70 (*red trace*) and between the NH1 atom of R14 and the O6 atom of G48 (*blue trace*). (*C*) The hydrogen bond distance between the NH2 atom of R15 and the O4 atom of U45 (*red trace*) and the salt bridge between the NH2 atom of R15 and the O1P atom of U45 (*blue trace*). All metrics are computed from the simulation with the lowest-work value. Darker colors signify the regions of interest. Lightly shaded horizontal lines indicate the initial values of the corresponding distances. Each panel is accompanied with snapshots highlighting the corresponding interactions extracted from a time point marked by an arrow. Each amino acid, nucleotide, and atom that participates in hydrogen-bonding or salt-bridging interactions is uniquely colored. The scale on *y* axis is limited to 15 Å because the presented interactions form at distances below 3.5 Å. The data corresponding to all distance values on the *y* axis are shown in Fig. S9 *A*. To see this figure in color, go online.

with the possibility to interact with the C44, U45, and G46 nucleotides (Fig. S7 *A*). However, at t = ∼1.9 ns, we observed the formation of only one salt bridge that was formed between the NH2 atom of R15 amino acid and the O1P atom of U45 (*blue trace*, Fig. 3 *C*), which was preceded by the rupture of the hydrogen bond at t = ∼0.95 ns between the NH2 atom of R15 amino acid and the O4 atom of U45 while the peptide was still located in the binding pocket (*red trace*, Fig. 3 *C*). The peptide was free of any interactions with the RNA at a distance of 35 Å (t = 5.6 ns).

Pathway 2

In PW2, we observed different mechanistic details underlying the dissociation process in comparison with PW1, which likely contributed to a lower free-energy barrier to dissociation (Fig. 2 *B*). As the peptide began dissociating out of the binding pocket (Fig. S7 *B*), the van der Waals interactions between the R8 amino acid and the U66 nucleotide were broken at t = ∼0.1 ns (*purple trace*, Fig. S8 *B*). This event occurred simultaneously with the rupture of the hydrogen bond between the NH2 atom of R8 amino acid and the O6 atom of G64 at t = ∼0.1 ns (*red*, Fig. 4 *A*). The R8 amino acid did not form any stable close contact interactions until t = ∼0.9 ns, when the NH1 atom of R8 formed a salt bridge with the O1P atom of G48. At t = ∼0.73 ns, the hydrogen bond between the NH1 atom of R14 and the O6 atom of G70 (which was preformed in the initial binding pocket) broke, and the NH2 atom of R14 formed a salt bridge with the O2P atom of A68 at t = ∼0.75 ns (Fig. 4 *B*). Thus, two arginine amino acids, R8 and R14, formed salt-bridging interactions at t = ∼0.9 ns, creating a network of salt bridges from the G48 nucleotide to the A68 nucleotide (Fig. S11 *A*). This conformation also resulted in a metastable state that was highlighted in the free-energy profile at ∼5.4 Å (*cyan M*, Fig. S6 *B*).

In PW2, the NH1 atom of R15 formed a salt bridge with the O1P atom of C44 (*red trace*, Fig. 4 *C*) when the peptide was in the vicinity of the backbone atoms of the C44, U45, and G46 nucleotides at t = ∼1 ns (Fig. S7 *B*). Importantly, at t = ∼1.3 ns, the NH2 atom of R15 formed a salt bridge with the O2P atom of U45 (*blue trace*, Fig. 4 *C*). Thus, between t = ∼1.3 and t = ∼1.5 ns, the NH1 and NH2 atoms of R15 were fluctuating to simultaneously form two salt bridges with the O1P and O2P atoms of C44 and U45 nucleotides, respectively (Fig. 4 *C*). This motion was another factor that contributed to a decrease in the free-energy barrier in comparison with PW1. In addition to that, the rupture of the hydrogen bond between the NH2 atom of R8 amino acid and the O6 atom of G64 at t = ∼0.1 ns and the rupture of the van der Waals interactions between the R8 amino acid and the U66 at t = ∼0.1 ns also contributed to a decrease in the free-energy barrier in comparison with PW1. The peptide was free of any interactions with the RNA at a distance of 35 Å (t = 5.6 ns).

Pathway 3

In PW3, the peptide required ∼3 ns to escape the binding pocket, whereas in PW1 and PW2, the peptide escaped the binding pocket in ∼2 ns (Fig. S7, *A*–*C*). This was in part due to the interactions of various amino acids with the A68 nucleotide in PW3 (Fig. S7 *C*) as well as due to the interactions between the R8 amino acid and the U66 nucleotide that we characterized using the van der Waals energy (*purple trace*, Fig. S8 *C*). These interactions resulted in

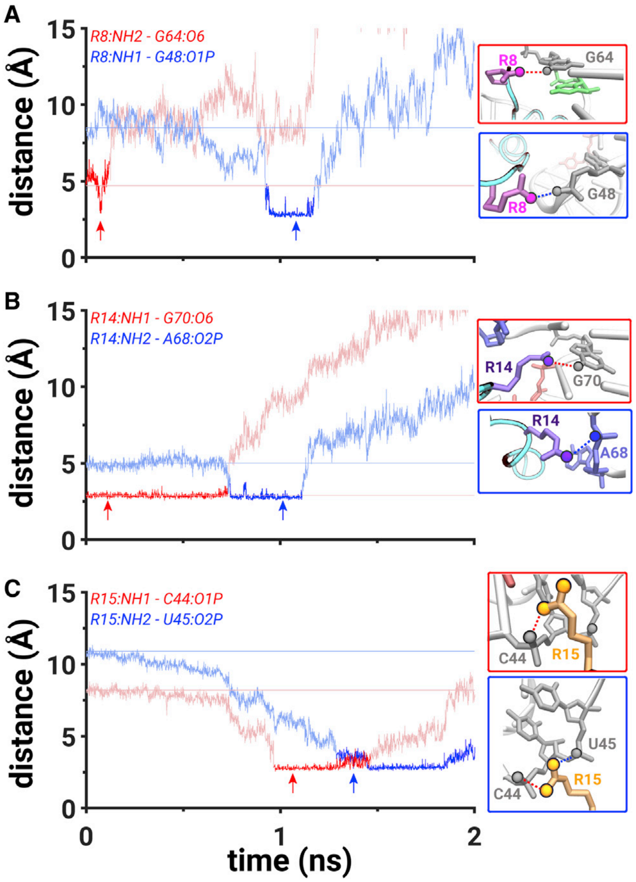

FIGURE 4 Mechanistic details of PW2. (*A*) The hydrogen bond distance between the NH2 atom of R8 and the O6 atom of G64 (*red trace*) and the salt bridge between the NH1 atom of R8 and the O1P atom of G48 (*blue trace*). (*B*) The hydrogen bond distance between the NH1 atom of R14 and the O6 atom of G70 (*red trace*) and the salt bridge between the NH2 atom of R14 and the O2P atom of A68 (*blue trace*). (*C*) The salt bridges between NH1 atom of R15 and the O1P atom of C44 (*red trace*) and between the NH2 atom of R15 and the O2P atom of U45 (*blue trace*). The scale on *y* axis is limited to 15 Å because the presented interactions form at distances below 3.5 Å. The data corresponding to all distance values on the *y* axis are shown in Fig. S9 *B*; cf. Fig. 3 for all other details. To see this figure in color, go online.

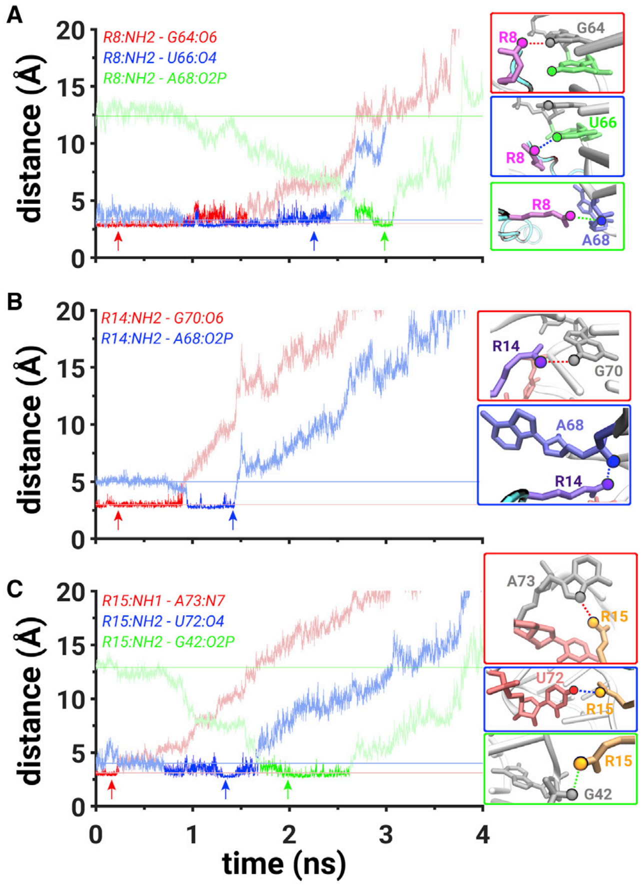

FIGURE 5 Mechanistic details of PW3. (*A*) The hydrogen bond distances between the NH2 atom of R8 and the O6 atom G64 (*red trace*) and between the NH2 atom R8 and the O4 atom of U66 (*blue trace*) and the salt bridge between the NH2 atom of R8 and the O2P atom of A68 (*green trace*). (*B*) The hydrogen bond distance between the NH2 atom of R14 and the O6 atom of G70 (*red trace*) and the salt bridge between the NH2 atom of R14 and the O2P atom of A68 (*blue trace*). (*C*) The hydrogen bond distances between the NH1 atom R15 and the N7 atom of A73 (*red trace*) and between the NH2 atom of R15 and the O4 atom of U72 (*blue trace*) and the salt bridge between the NH2 atom of R15 and the O2P atom of G42 (*green trace*). The scale on *y* axis is limited to 20 Å because the presented interactions form at distances below 3.5 Å. The data corresponding to all values of distances on the *y* axis are shown in Fig. S9 *C*; cf. Fig. 3 for all other details. To see this figure in color, go online.

a partial unfolding of the peptide coil between t = ~1.8 and t = ~3 ns (Fig. S7 *C*).

During the first 0.8 ns of the simulation, the peptide disrupted interactions between the R18 amino acid and the A68 nucleotide, as characterized by the van der Waals energy (*brown trace*, Fig. S8 *C*), and started dissociating. A hydrogen bond between the NH2 atom of R8 amino acid and the O6 atom of G64 weakened at t = ~0.9 ns (*red trace*, Fig. 5 *A*) and the NH2 atom of R8 amino acid started forming a new hydrogen bond with the O4 atom of U66 at t = ~1 ns (*blue trace*, Fig. 5 *A*). At t = ~0.9 ns, the hydrogen bond between the NH2 atom of R14 amino acid and the O6 atom of G70 ruptured (*red trace*, Fig. 5 *B*) and the NH2 atom of R14 amino acid formed a salt bridge with the O2P atom of A68 (*blue trace*, Fig. 5 *B*).

At t = ~0.25 ns, the NH1 atom of R15 amino acid stopped forming the hydrogen bond with the N7 atom of A73, and the NH2 atom of R15 formed a hydrogen bond with the O4 atom of U72. Thus, the combined interactions between the NH2 atom of R8 amino acid and the O4 atom of U66, between the NH2 atom of R14 amino acid and the O2P atom of A68, and between the NH2 atom of R15 amino acid and the O4 atom of U72 created a network of salt-bridging and hydrogen-bonding interactions at ~1 ns and lasted for ~0.5 ns (Fig. S11 *B*).

At t = ~1.7 ns, the hydrogen bond between the NH2 atom of R15 amino acid and the O4 atom of U72 ruptured (*blue trace*, Fig. 5 *C*), and a salt bridge was formed between the

NH2 atom of R15 and the O2P atom of the G42, which broke at t = ~2.7 ns (*green trace*, Fig. 5 *C*). At t = ~2.7 ns, a salt bridge was formed between the NH2 atom of R8 amino acid and the O2P atom of A68 that lasted for ~0.2 ns (*green trace*, Fig. 5 *A*). Thus, salt-bridging interactions were forming during every step of the dissociation process in PW3. The peptide was free of any interactions with the RNA at a distance of 40 Å (t = 6.4 ns).

Pathway 4

Finally, in PW4, which had the lowest free-energy barrier to dissociation (Fig. S6 *D*), the mechanism of dissociation was similar to PW3, but we observed several key differences. During the first 0.8 ns of the simulation, the interactions between the R8 amino acid and the U66 nucleotide and the R18 amino acid and the A68 nucleotide weakened, as characterized by the van der Waals interaction energy (Fig. S8 *D*). The NH2 atom of R8 amino acid formed a hydrogen bond with the O6 atom of G64 at t = ~0.35 ns and broke it at t = ~0.55 ns (*red trace*, Fig. 6 *A*). After that, the R8 amino acid did not form any stable interactions until t = ~2 ns (Fig. S7 *D*). At t = ~0.8 ns, the hydrogen bond between the NH1 atom of R14 amino acid and the O6 atom of G70 ruptured (*red trace*, Fig. 6 *B*), and a salt bridge was formed between the NH1 atom of R14 amino acid and the O1P atom of G69 (*blue trace*, Fig. 6 *B*). At t = ~0.65 ns, a hydrogen bond was formed between the NH2 atom of R15 amino acid and the O2 atom of U72 (*blue trace*, Fig. 6 *C*), which was preceded by the rupture of the hydrogen bond (at t = ~0.6 ns) between the NH1 atom of R15 amino acid and the N7 atom of A73 (*red trace*, Fig. 6 *A*). The salt bridge between the NH1 atoms of R14 amino acid with the O1P atom of C69 and the hydrogen bond between the NH2 atom of R15 amino acid with the O2 atom of U72 formed a network of salt-bridging and hydrogen-bonding interactions at ~0.8 ns (Fig. S11 *C*), which corresponded to a metastable state at ~5.1 Å (*blue M*, Fig. S6 *D*).

At t = ~1.3 ns, the NH2 atom of R14 amino acid formed another salt bridge with the O2P atom of A68 (*green trace*, Fig. 6 *B*). The NH2 atom of R15 amino acid ruptured the hydrogen bond with the O2 atom of U72 and formed a salt bridge with the O1P atom of C44 at t = ~1.1 ns (Fig. 6 *C*). At t = ~2 ns, the NH2 atom of R8 amino acid formed a hydrogen bond with the O6 atom of G70 (*blue trace*, Fig. 6 *A*) and combined with the salt bridge between the NH2 atom of R15 amino acid and the O1P atom of C44; the second network of hydrogen-bonding and salt-bridging interactions was created in PW4 (Fig. S11 *D*) and corresponded to a metastable state at ~6.9 Å (*blue M*, Fig. S6 *D*). At t = ~2.4 ns, a hydrogen bond between the NH2 atom of R8 amino acid and the O6 atom of G70 ruptured, and a salt bridge was formed between the NH1 atom of

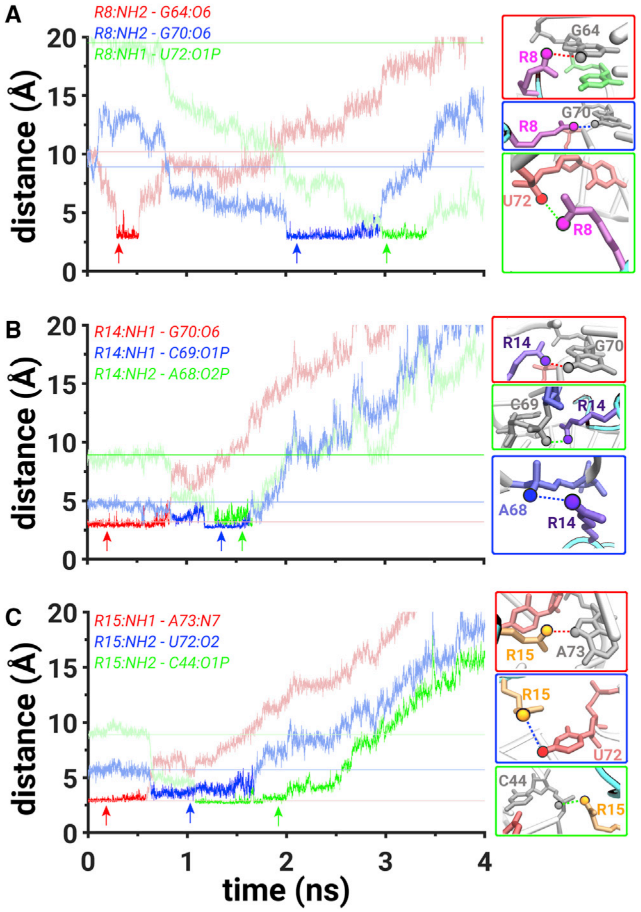

FIGURE 6 Mechanistic details of PW4. (*A*) The hydrogen bond distances between the NH2 atom of R8 and the O6 atom of G64 (*red trace*) and between the NH2 atom of R8 and the O6 atom of G70 (*blue trace*) and the salt bridge between the NH1 atom of R8 and the O1P atom of U72 (*green trace*). (*B*) The hydrogen bond distance between the NH1 atom of R14 and the O6 atom of G70 (*red trace*) and the salt bridges between the NH1 atom of R14 and the O1P atom of C69 (*blue trace*) and between the NH2 atom of R14 and the O2P atom of A68 (*green trace*). (*C*) The hydrogen bond distances between NH1 atom of R15 and the N7 atom of A73 (*red trace*) and between the NH2 atom of R15 and the O2 atom of U72 (*blue trace*) and the salt bridge between the NH2 atom of R15 and the O1P atom of C44 (*green trace*). The scale on *y* axis is limited to 20 Å because the presented interactions form at distances below 3.5 Å. The data corresponding to all distance values on the *y* axis are shown in Fig. S9 *D*; cf. Fig. 3 for all other details. To see this figure in color, go online.

R8 and the O1P atom of U72 (*green trace*, Fig. 6 *A*). The peptide was free of any interactions with the RNA at a distance of 40 Å (*t* = 6.4 ns).

Overall, we observed the formation of unique interactions in each pathway, including the formation of salt-bridging and hydrogen-bonding interactions. Our observations suggest that there is a network of salt bridges and hydrogen bonds that was formed in each pathway, with the exception of PW1, which had the smallest number of hydrogen bonds and salt bridges formed in comparison with other pathways.

Water dynamics in the binding pocket

Water is known to play a critical role in ligand recognition (113) because ligand binding-unbinding may be accompanied by loss or gain of water molecules in the binding pocket. Therefore, we investigated the solvation of the binding pocket of the RRE RNA during peptide dissociation along two pathways (PW1 and PW4). These pathways were identified to have the highest and the lowest free-energy barriers, respectively, for dissociation of the peptide. We sought to understand the role of water molecules and their contribution to differences in the free-energy barriers along these two pathways.

Before the dissociation process was initiated, we observed around 40 water molecules around the peptide in the binding pocket of the RRE RNA (Fig. S12). As the peptide dissociated along PW1, we initially observed a slow increase in the number of water molecules (N_W) to 50 water molecules, signifying the influx of water in the binding pocket. Between t = ~0.65 and t = ~0.85 ns, the value of N_W did not significantly change, which was also characterized by the formation and further rupture of the hydrogen bonds between the NH2 atom of the R8 amino acid and the O6 atoms of the G64 and U66 nucleotides (Fig. 3 *A*, Fig. S12). At t = ~0.9, we observed a more rapid increase in N_W that converged to ~110 water molecules at t = ~1.4 ns when the peptide dissociated from the binding pocket (*red trace*, Fig. S12). We also noticed a small decrease in N_W at t = ~1.05 ns that was coupled with the interaction between the residues R8, R14, and R15 and the RNA backbone (Fig. S10).

However, we observed a different solvation pattern of the binding pocket in PW4 in comparison with PW1. Specifically, we initially observed desolvation of the binding site because the N_W-value decreased to ~30 water molecules until t = ~0.5 ns (*blue trace*, Fig. S12). This decrease was coupled with the movement of the peptide out of the binding pocket that led to water molecules moving out of the pocket because of the rearrangements in the amino acids. Specifically, the R8 residue was dynamic and moved in the binding pocket until the NH2 atom of R8 formed a hydrogen bond with the O6 atom of G64 (*red trace*, Fig. 6 *A*). At t = ~0.5 ns, the N_W-values started to increase, indicating the resolvation of the binding site (*blue trace*, Fig. S12). The resolvation continued until t = ~1.9 ns, when the main portion of the peptide escaped the binding pocket, whereas the unstructured coil segment of the peptide still remained in the binding pocket interacting with the nucleotides (Figs. S7 and S12). After the last amino acid escaped the binding pocket at t = ~3.2 ns, the binding pocket was fully solvated. Importantly, the final N_W-values for both pathways converged to similar number of water molecules in the binding pocket. Because we observed differences in the free-energy barriers of PW1 and PW4 during the earlier stage of dissociation of the peptide, the distinct water dynamics in PW4, where the binding pocket initially desolvated before resolvating, potentially contributed to a lower free-energy barrier for peptide dissociation.

DISCUSSION

In this work, we have studied the dissociation mechanism of the RSG-1.2 peptide from the RRE RNA along four distinct pathways using nonequilibrium SMD simulations. Although it has been previously proposed that the salt-bridging interactions could be important for the recognition of this peptide by the RRE RNA (83), there is no study on the binding-unbinding mechanism of this peptide in the literature. We observed the formation of unique salt-bridging and hydrogen-bonding interactions in each pathway that form in an ordered stepwise sequence in which the rupture of one interaction led to the creation of another interaction. We also estimated the free-energy profiles for each pathway using the Jarzynski's equality and observed distinct free-energy barriers in each pathway.

We observed the highest free-energy barrier of peptide dissociation in PW1 (Fig. 2 *B*), which was coupled with the displacement of the backbone atoms of the C44, U45, and G46 nucleotides (Fig. S10). Moreover, we observed only one salt bridge formed during dissociation in PW1 (Fig. 3 *C*; Table S2). The formation of this salt bridge between the R15 amino acid of the peptide and the U45 nucleotide of the RRE RNA was coupled with a weak recognition of the peptide by the RNA. This pathway was also characterized by a rapid solvation of the binding pocket (*red trace*, Fig. S12).

Even though the overall process of dissociation in PW2 was somewhat similar to PW1, the free-energy barrier in PW2 was smaller than in PW1 (Fig. S6*, A and B*). One of the key differences between PW1 and PW2 was the interaction between the R8 amino acid and the U66 nucleotide of the RNA that ruptured at t = ~0.1 ns in PW2, as characterized by the van der Waals interaction energy, whereas the rupture of the interaction between the R8 amino acid and the U66 nucleotide only occurred at t = ~0.8 ns in PW1 (*purple traces*, Fig. S8*, A and B*). We also observed an additional salt bridge in PW2 that was formed between the NH2 atom of R15 amino acid and the O1P atom of C44 (*red trace*, Fig. 4 *C*). This interaction was formed ~0.5 ns earlier in PW2 in comparison with a similar type of interaction between the NH2 atom of R15 amino acid and the O1P atom of U45 in PW1. The peptide passed in close proximity to the C44 and U45 nucleotides in both pathways, and a faster establishment of a salt-bridging interaction with an atom from one of these nucleotides is important for the recognition of the RNA backbone for the peptide if it dissociates along PW1 or PW2. The earlier rupture of the interaction between the R8 amino acid and the U66 nucleotide as well as a lack of displacement of the backbone atoms of

the C44, U45, and G46 nucleotides led to a decreased free-energy barrier in PW2.

The pathways PW3 and PW4 had smaller free-energy barriers in comparison with PW1 and PW2 (Fig. S6). It should be noted that in PW3 and PW4, the peptide required a longer time to dissociate in comparison with PW1 and PW2, which was caused by additional interactions that were forming between the flipped-out A68 nucleotide and the peptide as it was dissociating (Fig. S7, *C and D*). These interactions were not formed in PW1 and PW2 because the peptide was dissociating in a direction away from the A68 nucleotide (Fig. S7, *A and B*). Moreover, the dissociation reaction coordinate in PW3 and PW4 was free of any obstacles, such as the atoms of the RNA backbone in the C44, U45, and G46 nucleotides that were present in PW1 and PW2. Thus, a decrease in the free-energy barriers in PW3 and PW4 was achieved by reducing any steric overlap or the displacement of the atoms in the major groove of the RNA. Therefore, these two pathways, PW3 and PW4, are preferred in comparison with PW1 and PW2 due to lower free-energy barriers for peptide dissociation (Fig. S6). We also observed desolvation of the binding pocket (*blue trace*, Fig. S12) during the initial stages of dissociation of the peptide, which likely contributed to a lower free-energy barrier in comparison with other pathways.

However, PW4 exhibited an even smaller free-energy barrier of dissociation by ~7 kcal/mol in comparison with PW3, meaning that the pathway PW4 is further preferred over PW3. We also conducted umbrella sampling (114) simulations (see Supporting materials and methods, Methods) to recompute the free-energy profile and determine the free-energy barrier for dissociation of the peptide along PW4 (Fig. S13). These results showed that the free-energy barrier along PW4 was ~23.72 $\pm$ 1.46 kcal/mol, which was comparable with the value (24.46 kcal/mol) that we obtained using SMD simulations. We further tested the evolution of the reaction coordinate along PW4 using the swarms-of-trajectories string method (115) to verify that PW4 was a reliable pathway (see Supporting materials and methods, Methods). We computed the average root mean squared deviation (RMSD) of the entire string across each iteration as a measure of convergence of the string (Fig. S14). The initial pathway generated using SMD converged after 13 iterations, whereas the main fluctuations in the string RMSD were primarily due to the motion of the peptide in the bulk water (Fig. S14). Therefore, these additional tests indicate that the reaction coordinate along PW4 is reasonable and accurate for understanding thermodynamics of dissociation of the peptide. We conducted umbrella sampling and swarms-of-trajectories string method calculations using the AMBER software package (116) combined with the AMBER force field for RNA (RNA.ROC) (90) and for the peptide (ff14sb) (91). Additionally, we note that the usage of rotational restraints improves convergence of the free-energy profile (Fig. S15 *A*); the usage of secondary structure restraints prevents the unfolding of the peptide (Fig. S15 *B*), whereas the choice of the ensemble used in SMD simulations does not significantly affect the free-energy profile (Fig. S15 *C*).

The decrease in free-energy barrier in PW4 is likely a result of the behavior of the R8 amino acid, which did not form any stable interactions between ~0.5 and ~2 ns in PW4 (Fig. 6 *A*) while it was forming stable hydrogen-bonding interactions in that time range in PW3 (*red* and *blue traces*, Fig. 5 *A*). This behavior of the R8 amino acid was also reflected in the van der Waals interaction energies (*purple traces*, Fig. S8, *C and D*), which showed that the R8 amino acid had stronger interactions with the U66 nucleotide in PW3 in comparison with PW4.

By analyzing the salt-bridging and hydrogen-bonding interactions in each pathway, we determined that R8, R14, and R15 were the most critical amino acids for the recognition of the peptide by the RRE RNA. Each of these amino acids were involved in a complex network of salt bridges and hydrogen bonds in PW2, PW3, and PW4 (Figs. 4, 5, and 6; Fig. S11). Moreover, these amino acids interacted with the RNA nucleotides in a stepwise pattern in which the rupture of existing interactions resulted in the formation of new interactions with other nucleotides during the dissociation process. PW3 and PW4 exhibited the formation of additional hydrogen bonds and salt bridges in comparison with PW1 and PW2, which resulted from the extended dissociation timescales. Furthermore, we recomputed the free-energy profile (Fig. S16) along PW4 by mutating each of the three key arginine residues (R8, R14, and R15) to alanine residues using the psfgen plugin in VMD (95). These results showed a decrease in the free-energy barrier on mutations of key arginine residues, indicating that the peptide is significantly destabilized without interactions of these residues with the RRE RNA, thereby supporting their critical role in binding of this peptide.

In particular, we believe that the R8 amino acid was the most critical amino acid in the least free-energy barrier pathway PW4. Firstly, as mentioned before, the R8 amino acid had decreased interactions with the nucleotides of the RRE RNA between ~0.5 and ~2 ns, which led to a decreased free-energy barrier in PW4. Secondly, after the peptide dissociated from the initial binding pocket, the R8 amino acid was the only amino acid that was forming a stable interaction with the RNA nucleotide after ~3 ns. Specifically, the NH2 atom of the R8 amino acid formed a salt bridge with the O1P atom of the U72 nucleotide between ~3 and ~3.4 ns (*green trace*, Fig. 6 *A*). Thus, we hypothesize that for the reverse process of peptide binding along PW4, the R8 amino acid will be the first amino acid to form a stable interaction with the U72 nucleotide of the RRE RNA.

Additionally, it is critical to note that the RSG-1.2 protein was synthesized by mutagenesis from the Rev peptide (109) that binds the RRE RNA during the HIV-1 replication

process. One important mutation in that study was the mutation of the arginine amino acid in the Rev protein at position 9 to a proline amino acid. It was hypothesized that this mutation resulted in a decrease in electrostatic contacts between the arginine amino acids in the N-terminus of the peptide and could be potentially coupled with the increased binding affinity to the RRE RNA (109). However, it was not clear how the RSG-1.2 peptide recognized the RRE RNA during the binding process and which amino acids contributed the most to this process. In our work, we observed that the R8 amino acid, which is located next to the Rev protein at position 9 amino acid in the polypeptide chain, formed stable hydrogen-bonding and salt-bridging interactions in each pathway. The R8 amino acid also was the last amino acid to interact with the RRE RNA during the dissociation and thus could be the first to interact with the RRE RNA during the binding process. Thus, the ability of the R8 amino acid to form these interactions was rooted in its flexibility that was coupled with the formation of various interactions with the RRE RNA nucleotides and resulted in the increased binding affinity and specificity with the RRE RNA in comparison with the Rev protein.

CONCLUSIONS

The binding-unbinding of proteins or short peptides to RNA is an important biophysical process that is poorly understood. We used nonequilibrium cv-SMD simulations to study the dissociation mechanism of a helical peptide along four different pathways from the RRE RNA binding pocket to obtain key insights into the peptide binding-unbinding process and the recognition mechanism of this peptide. In particular, we investigated the mechanistic details of each pathway to identify interactions that are important for the recognition of proteins and peptides. We analyzed the resulting free-energy profiles and observed that the final free-energy differences were 96.47 $\pm$ 12.63 kcal/mol for PW1, 96.1 $\pm$ 10.95 kcal/mol for PW2, 91.83 $\pm$ 9.81 kcal/mol for PW3, and 92 $\pm$ 11.32 kcal/mol for PW4. Consistent with the similar initial (bound) and final (unbound) states of the peptide in each pathway, the resulting free-energy differences (ΔG) are consistent among different pathways. However, the free-energy profiles for each pathway exhibited different magnitudes of the free-energy barriers for dissociation of the peptide, leading to the observation that PW4 is the preferred pathway of dissociation. In addition, the peptide dissociation was coupled with the formation of metastable states that resulted from a network of salt bridges formed between the arginine amino acids and the phosphate groups of the RNA backbone as well as from the hydrogen bonding. Specifically, we identified that the R8, R14, and R15 amino acids were important for the peptide recognition by the RRE RNA. Our results also suggest the R8 amino acid to be the most critical amino acid out of the three arginine amino acids because of its increased flexibility and the ability to form a primary or terminal salt-bridging interaction with the U72 nucleotide during the binding-unbinding process in PW4. These observations are potentially important for the recognition mechanism between the RNA molecules and the proteins and peptides that have charged amino acids. The simulation scripts used to generate data in this work are available in Appendices D and F of the doctoral thesis by Lev Levintov (117).

SUPPORTING MATERIAL

Supporting material can be found online at https://doi.org/10.1016/j.bpj.2021.10.007.

AUTHOR CONTRIBUTIONS

L.L. and H.V. designed the research. L.L. performed the research and analyzed the data. L.L. and H.V. wrote the article.

ACKNOWLEDGMENTS

We acknowledge financial support from the National Science Foundation (CBET-1554558) and the National Institutes of Health through grant R35GM138217. The content is solely the responsibility of the authors and does not necessarily represent the official views of the National Institutes of Health. We acknowledge computational support through the following resources: Premise, a central shared high-performance computing cluster at University of New Hampshire supported by the Research Computing Center; BioMade, a heterogeneous CPU-GPU cluster supported by the National Science Foundation Established Program to Stimulate Competitive Research award (OIA-1757371); and the National-Science-Foundation-supported (ACI-1548562) Extreme Science and Engineering Discovery Environment (XSEDE) (118) Comet resource at San Diego Supercomputer Center under grant TG-MCB160183 (to H.V.).

REFERENCES

1. Chen, Y., and G. Varani. 2005. Protein families and RNA recognition. *FEBS J.* 272:2088–2097.
2. Licatalosi, D. D., and R. B. Darnell. 2010. RNA processing and its regulation: global insights into biological networks. *Nat. Rev. Genet.* 11:75–87.
3. Holmqvist, E., and J. Vogel. 2018. RNA-binding proteins in bacteria. *Nat. Rev. Microbiol.* 16:601–615.
4. Guttman, M., and J. L. Rinn. 2012. Modular regulatory principles of large non-coding RNAs. *Nature.* 482:339–346.
5. Rinn, J. L., and H. Y. Chang. 2012. Genome regulation by long non-coding RNAs. *Annu. Rev. Biochem.* 81:145–166.
6. Francisco-Velilla, R., E. B. Azman, and E. Martinez-Salas. 2019. Impact of RNA–protein interaction modes on translation control: the versatile multidomain protein Gemin5. *BioEssays.* 41:e1800241.
7. Cooper, T. A., L. Wan, and G. Dreyfuss. 2009. RNA and disease. *Cell.* 136:777–793.
8. Esteller, M. 2011. Non-coding RNAs in human disease. *Nat. Rev. Genet.* 12:861–874.
9. Yildirim, I., D. Chakraborty, ..., G. C. Schatz. 2015. Computational investigation of RNA CUG repeats responsible for myotonic dystrophy 1. *J. Chem. Theory Comput.* 11:4943–4958.

10. Schmitt, A. M., and H. Y. Chang. 2016. Long noncoding RNAs in cancer pathways. *Cancer Cell.* 29:452–463.
11. Aboul-ela, F., J. Karn, and G. Varani. 1995. The structure of the human immunodeficiency virus type-1 TAR RNA reveals principles of RNA recognition by Tat protein. *J. Mol. Biol.* 253:313–332.
12. Bieniasz, P., and A. Telesnitsky. 2018. Multiple, switchable protein:RNA interactions regulate human immunodeficiency virus type 1 assembly. *Annu. Rev. Virol.* 5:165–183.
13. Ivanyi-Nagy, R., I. Kanevsky, …, J. L. Darlix. 2006. Analysis of hepatitis C virus RNA dimerization and core-RNA interactions. *Nucleic Acids Res.* 34:2618–2633.
14. Schmidt, N., C. A. Lareau, …, M. Munschauer. 2021. The SARS-CoV-2 RNA-protein interactome in infected human cells. *Nat. Microbiol.* 6:339–353.
15. Puglisi, J. D., L. Chen, …, A. D. Frankel. 1995. Solution structure of a bovine immunodeficiency virus Tat-TAR peptide-RNA complex. *Science.* 270:1200–1203.
16. Valverde, R., L. Edwards, and L. Regan. 2008. Structure and function of KH domains. *FEBS J.* 275:2712–2726.
17. Patel, D. J. 1999. Adaptive recognition in RNA complexes with peptides and protein modules. *Curr. Opin. Struct. Biol.* 9:74–87.
18. Dai, Y., J. E. Wynn, …, W. L. Santos. 2018. Discovery of a branched peptide that recognizes the Rev response element (RRE) RNA and blocks HIV-1 replication. *J. Med. Chem.* 61:9611–9620.
19. Walker, M. J., and G. Varani. 2019. Structure-based design of RNA-binding peptides. *Methods Enzymol.* 623:339–372.
20. Frankel, A. D. 2000. Fitting peptides into the RNA world. *Curr. Opin. Struct. Biol.* 10:332–340.
21. Frenkel-Pinter, M., J. W. Haynes, …, L. J. Leman. 2020. Mutually stabilizing interactions between proto-peptides and RNA. *Nat. Commun.* 11:3137.
22. Stefl, R., F. C. Oberstrass, …, F. H. Allain. 2010. The solution structure of the ADAR2 dsRBM-RNA complex reveals a sequence-specific readout of the minor groove. *Cell.* 143:225–237.
23. Corley, M., M. C. Burns, and G. W. Yeo. 2020. How RNA-binding proteins interact with RNA: molecules and mechanisms. *Mol. Cell.* 78:9–29.
24. Das, C., and A. D. Frankel. 2003. Sequence and structure space of RNA-binding peptides. *Biopolymers.* 70:80–85.
25. Iwazaki, T., X. Li, and K. Harada. 2005. Evolvability of the mode of peptide binding by an RNA. *RNA.* 11:1364–1373.
26. Hemmerich, P., S. Bosbach, …, U. Krawinkel. 1997. Human ribosomal protein L7 binds RNA with an alpha-helical arginine-rich and lysine-rich domain. *Eur. J. Biochem.* 245:549–556.
27. Godin, K. S., and G. Varani. 2007. How arginine-rich domains coordinate mRNA maturation events. *RNA Biol.* 4:69–75.
28. Truant, R., and B. R. Cullen. 1999. The arginine-rich domains present in human immunodeficiency virus type 1 Tat and Rev function as direct importin beta-dependent nuclear localization signals. *Mol. Cell. Biol.* 19:1210–1217.
29. Casu, F., B. M. Duggan, and M. Hennig. 2013. The arginine-rich RNA-binding motif of HIV-1 Rev is intrinsically disordered and folds upon RRE binding. *Biophys. J.* 105:1004–1017.
30. Scanlon, M. J., D. P. Fairlie, …, M. L. West. 1995. NMR solution structure of the RNA-binding peptide from human immunodeficiency virus (type 1) Rev. *Biochemistry.* 34:8242–8249.
31. Battiste, J. L., H. Mao, …, J. R. Williamson. 1996. Alpha helix-RNA major groove recognition in an HIV-1 rev peptide-RRE RNA complex. *Science.* 273:1547–1551.
32. Legault, P., J. Li, …, J. Greenblatt. 1998. NMR structure of the bacteriophage lambda N peptide/boxB RNA complex: recognition of a GNRA fold by an arginine-rich motif. *Cell.* 93:289–299.
33. Gosser, Y., T. Hermann, …, D. J. Patel. 2001. Peptide-triggered conformational switch in HIV-1 RRE RNA complexes. *Nat. Struct. Biol.* 8:146–150.
34. Daugherty, M. D., I. D'Orso, and A. D. Frankel. 2008. A solution to limited genomic capacity: using adaptable binding surfaces to assemble the functional HIV Rev oligomer on RNA. *Mol. Cell.* 31:824–834.
35. Tan, R., L. Chen, …, A. D. Frankel. 1993. RNA recognition by an isolated alpha helix. *Cell.* 73:1031–1040.
36. Bayer, T. S., L. N. Booth, …, A. D. Ellington. 2005. Arginine-rich motifs present multiple interfaces for specific binding by RNA. *RNA.* 11:1848–1857.
37. Daugherty, M. D., B. Liu, and A. D. Frankel. 2010. Structural basis for cooperative RNA binding and export complex assembly by HIV Rev. *Nat. Struct. Mol. Biol.* 17:1337–1342.
38. DiMattia, M. A., N. R. Watts, …, J. M. Grimes. 2010. Implications of the HIV-1 Rev dimer structure at 3.2 A resolution for multimeric binding to the Rev response element. *Proc. Natl. Acad. Sci. USA.* 107:5810–5814.
39. Acevedo, R., D. Evans, …, S. A. Showalter. 2016. Binding by TRBP-dsRBD2 does not induce bending of double-stranded RNA. *Biophys. J.* 110:2610–2617.
40. Krepl, M., M. Blatter, …, J. Sponer. 2017. Structural study of the Fox-1 RRM protein hydration reveals a role for key water molecules in RRM-RNA recognition. *Nucleic Acids Res.* 45:8046–8063.
41. Figiel, M., M. Krepl, …, M. Nowotny. 2018. Mechanism of polypurine tract primer generation by HIV-1 reverse transcriptase. *J. Biol. Chem.* 293:191–202.
42. Draper, D. E. 1999. Themes in RNA-protein recognition. *J. Mol. Biol.* 293:255–270.
43. Nadassy, K., S. J. Wodak, and J. Janin. 1999. Structural features of protein-nucleic acid recognition sites. *Biochemistry.* 38:1999–2017.
44. Iwakiri, J., H. Tateishi, …, N. Kenmochi. 2012. Dissecting the protein-RNA interface: the role of protein surface shapes and RNA secondary structures in protein-RNA recognition. *Nucleic Acids Res.* 40:3299–3306.
45. Krüger, D. M., S. Neubacher, and T. N. Grossmann. 2018. Protein-RNA interactions: structural characteristics and hotspot amino acids. *RNA.* 24:1457–1465.
46. Salmon, L., G. M. Giambaşu, …, H. M. Al-Hashimi. 2015. Modulating RNA alignment using directional dynamic kinks: application in determining an atomic-resolution ensemble for a hairpin using NMR residual dipolar couplings. *J. Am. Chem. Soc.* 137:12954–12965.
47. Umuhire Juru, A., N. N. Patwardhan, and A. E. Hargrove. 2019. Understanding the contributions of conformational changes, thermodynamics, and kinetics of RNA-small molecule interactions. *ACS Chem. Biol.* 14:824–838.
48. Mackerell, A. D., Jr., and L. Nilsson. 2008. Molecular dynamics simulations of nucleic acid-protein complexes. *Curr. Opin. Struct. Biol.* 18:194–199.
49. Reyes, C. M., and P. A. Kollman. 1999. Molecular dynamics studies of U1A-RNA complexes. *RNA.* 5:235–244.
50. Kurisaki, I., M. Takayanagi, and M. Nagaoka. 2014. Combined mechanism of conformational selection and induced fit in U1A-RNA molecular recognition. *Biochemistry.* 53:3646–3657.
51. Reyes, C. M., and P. A. Kollman. 2000. Structure and thermodynamics of RNA-protein binding: using molecular dynamics and free energy analyses to calculate the free energies of binding and conformational change. *J. Mol. Biol.* 297:1145–1158.
52. Blakaj, D. M., K. J. McConnell, …, A. M. Baranger. 2001. Molecular dynamics and thermodynamics of protein-RNA interactions: mutation of a conserved aromatic residue modifies stacking interactions and structural adaptation in the U1A-stem loop 2 RNA complex. *J. Am. Chem. Soc.* 123:2548–2551.
53. Kormos, B. L., Y. Benitex, …, D. L. Beveridge. 2007. Affinity and specificity of protein U1A-RNA complex formation based on an additive component free energy model. *J. Mol. Biol.* 371:1405–1419.

54. Guo, J. X., and W. H. Gmeiner. 2001. Molecular dynamics simulation of the human U2B" protein complex with U2 snRNA hairpin IV in aqueous solution. *Biophys. J.* 81:630–642.

55. Schmid, N., B. Zagrovic, and W. F. van Gunsteren. 2007. Mechanism and thermodynamics of binding of the polypyrimidine tract binding protein to RNA. *Biochemistry.* 46:6500–6512.

56. Krepl, M., A. Cléry, …, J. Sponer. 2016. Synergy between NMR measurements and MD simulations of protein/RNA complexes: application to the RRMs, the most common RNA recognition motifs. *Nucleic Acids Res.* 44:6452–6470.

57. Diarra Dit Konté, N., M. Krepl, …, F. H. Allain. 2017. Aromatic side-chain conformational switch on the surface of the RNA Recognition Motif enables RNA discrimination. *Nat. Commun.* 8:654.

58. Estarellas, C., M. Otyepka, …, J. Šponer. 2015. Molecular dynamic simulations of protein/RNA complexes: CRISPR/Csy4 endoribonuclease. *Biochim. Biophys. Acta.* 1850:1072–1090.

59. Figiel, M., M. Krepl, …, M. Nowotny. 2017. Coordination between the polymerase and RNase H activity of HIV-1 reverse transcriptase. *Nucleic Acids Res.* 45:3341–3352.

60. Palermo, G., Y. Miao, …, J. A. McCammon. 2016. Striking plasticity of CRISPR-Cas9 and key role of non-targetDNA, as revealed by molecular simulations. *ACS Cent. Sci.* 2:756–763.

61. Palermo, G., Y. Miao, …, J. A. McCammon. 2017. CRISPR-Cas9 conformational activation as elucidated from enhanced molecular simulations. *Proc. Biophys J.* 114:7260–7265.

62. Castrignanò, T., G. Chillemi, …, A. Desideri. 2002. Molecular dynamics simulation of the RNA complex of a double-stranded RNA-binding domain reveals dynamic features of the intermolecular interface and its hydration. *Biophys J.* 83:3542–3552.

63. Xia, Z., Z. Zhu, …, R. Zhou. 2009. Recognition mechanism of siRNA by viral p19 suppressor of RNA silencing: a molecular dynamics study. *Biophys. J.* 96:1761–1769.

64. Yang, J., J. Song, …, C. Ji. 2015. Effect of mismatch on binding of ADAR2/GluR-2 pre-mRNA complex. *J. Mol. Model.* 21:222.

65. Wang, X., L. Vukovic, …, S. Myong. 2015. Dynamic profiling of double-stranded RNA binding proteins. *Nucleic Acids Res.* 43:7566–7576.

66. Drusin, S. I., I. P. Suarez, …, D. M. Moreno. 2016. dsRNA-protein interactions studied by molecular dynamics techniques. Unravelling dsRNA recognition by DCL1. *Arch. Biochem. Biophys.* 596:118–125.

67. Xue, Q., Q. C. Zheng, …, H. X. Zhang. 2014. Exploring the mechanism how Marburg virus VP35 recognizes and binds dsRNA by molecular dynamics simulations and free energy calculations. *Biopolymers.* 101:849–860.

68. Harikrishna, S., and P. I. Pradeepkumar. 2017. Probing the binding interactions between chemically modified siRNAs and human Argonaute 2 using microsecond molecular dynamics simulations. *J. Chem. Inf. Model.* 57:883–896.

69. Réblová, K., N. Spacková, …, J. Sponer. 2004. Long-residency hydration, cation binding, and dynamics of loop E/helix IV rRNA-L25 protein complex. *Biophys. J.* 87:3397–3412.

70. Créty, T., and T. E. Malliavin. 2007. The conformational landscape of the ribosomal protein S15 and its influence on the protein interaction with 16S RNA. *Biophys. J.* 92:2647–2665.

71. Chen, K., J. Eargle, …, Z. Luthey-Schulten. 2010. Functional role of ribosomal signatures. *Biophys. J.* 99:3930–3940.

72. Krepl, M., K. Réblová, …, J. Sponer. 2013. Bioinformatics and molecular dynamics simulation study of L1 stalk non-canonical rRNA elements: kink-turns, loops, and tetraloops. *J. Phys. Chem. B.* 117:5540–5555.

73. Yamasaki, S., S. Nakamura, …, K. Shimizu. 2007. Mechanism of the difference in the binding affinity of *E. coli* tRNAGln to glutaminyl-tRNA synthetase caused by noninterface nucleotides in variable loop. *Biophys. J.* 92:192–200.

74. Ghosh, A., and S. Vishveshwara. 2007. A study of communication pathways in methionyl- tRNA synthetase by molecular dynamics simulations and structure network analysis. *Proc. Natl. Acad. Sci. USA.* 104:15711–15716.

75. Sethi, A., J. Eargle, …, Z. Luthey-Schulten. 2009. Dynamical networks in tRNA:protein complexes. *Proc. Natl. Acad. Sci. USA.* 106:6620–6625.

76. Bhattacharyya, M., A. Ghosh, …, S. Vishveshwara. 2010. Allostery and conformational free energy changes in human tryptophanyl-tRNA synthetase from essential dynamics and structure networks. *Proteins.* 78:506–517.

77. Ghosh, A., R. Sakaguchi, …, Y. M. Hou. 2011. Allosteric communication in cysteinyl tRNA synthetase: a network of direct and indirect readout. *J. Biol. Chem.* 286:37721–37731.

78. Bushnell, E. A. C., W. Huang, …, J. W. Gauld. 2012. Molecular dynamics investigation into substrate binding and identity of the catalytic base in the mechanism of Threonyl-tRNA synthetase. *J. Phys. Chem. B.* 116:5205–5212.

79. Li, R., L. M. Macnamara, …, S. S. Cho. 2015. MD simulations of tRNA and aminoacyl-tRNA synthetases: dynamics, folding, binding, and allostery. *Int. J. Mol. Sci.* 16:15872–15902.

80. Nifosì, R., C. M. Reyes, and P. A. Kollman. 2000. Molecular dynamics studies of the HIV-1 TAR and its complex with argininamide. *Nucleic Acids Res.* 28:4944–4955.

81. Reyes, C. M., R. Nifosì, …, P. A. Kollman. 2001. Molecular dynamics and binding specificity analysis of the bovine immunodeficiency virus BIV Tat-TAR complex. *Biophys. J.* 80:2833–2842.

82. Mu, Y., and G. Stock. 2006. Conformational dynamics of RNA-peptide binding: a molecular dynamics simulation study. *Biophys. J.* 90:391–399.

83. Michael, L. A., J. A. Chenault, …, M. C. Nagan. 2009. Water, shape recognition, salt bridges, and cation-pi interactions differentiate peptide recognition of the HIV rev-responsive element. *J. Mol. Biol.* 392:774–786.

84. Mori, M., U. Dietrich, …, M. Botta. 2010. Molecular dynamics and DFT study on HIV-1 nucleocapsid protein-7 in complex with viral genome. *J. Chem. Inf. Model.* 50:638–650.

85. Do, T. N., E. Ippoliti, …, M. Parrinello. 2012. Counterion redistribution upon binding of a Tat-protein mimic to HIV-1 TAR RNA. *J. Chem. Theory Comput.* 8:688–694.

86. Li, C. H., Z. C. Zuo, …, C. X. Wang. 2013. The interactions and recognition of cyclic peptide mimetics of Tat with HIV-1 TAR RNA: a molecular dynamics simulation study. *J. Biomol. Struct. Dyn.* 31:276–287.

87. Šponer, J., G. Bussi, …, M. Otyepka. 2018. RNA structural dynamics as captured by molecular simulations: a comprehensive overview. *Chem. Rev.* 118:4177–4338.

88. Fernandes, J., B. Jayaraman, and A. Frankel. 2012. The HIV-1 Rev response element: an RNA scaffold that directs the cooperative assembly of a homo-oligomeric ribonucleoprotein complex. *RNA Biol.* 9:6–11.

89. Phillips, J. C., R. Braun, …, K. Schulten. 2005. Scalable molecular dynamics with NAMD. *J. Comput. Chem.* 26:1781–1802.

90. Aytenfisu, A. H., A. Spasic, …, D. H. Mathews. 2017. Revised RNA dihedral parameters for the Amber force field improve RNA molecular dynamics. *J. Chem. Theory Comput.* 13:900–915.

91. Maier, J. A., C. Martinez, …, C. Simmerling. 2015. ff14SB: improving the accuracy of protein side chain and backbone parameters from ff99SB. *J. Chem. Theory Comput.* 11:3696–3713.

92. Jorgensen, W. L., J. Chandrasekhar, …, M. L. Klein. 1983. Comparison of simple potential functions for simulating liquid water. *J. Chem. Phys.* 79:926.

93. Li, P., L. F. Song, and K. M. Merz, Jr. 2015. Systematic parameterization of monovalent ions employing the nonbonded model. *J. Chem. Theory Comput.* 11:1645–1657.

94. Roe, D. R., and T. E. Cheatham, III. 2013. PTRAJ and CPPTRAJ: software for processing and analysis of molecular dynamics trajectory data. *J. Chem. Theory Comput.* 9:3084–3095.

95. Humphrey, W., A. Dalke, and K. Schulten. 1996. VMD: visual molecular dynamics. *J. Mol. Graph.* 14:33–38, 27–28.

96. Ettig, R., N. Kepper, …, K. Rippe. 2011. Dissecting DNA-histone interactions in the nucleosome by molecular dynamics simulations of DNA unwrapping. *Biophys. J.* 101:1999–2008.

97. Gupta, A., and M. Bansal. 2016. The role of sequence in altering the unfolding pathway of an RNA pseudoknot: a steered molecular dynamics study. *Phys. Chem. Chem. Phys.* 18:28767–28780.

98. Vashisth, H., and C. F. Abrams. 2008. Ligand escape pathways and (un)binding free energy calculations for the hexameric insulin-phenol complex. *Biophys. J.* 95:4193–4204.

99. Capelli, A. M., and G. Costantino. 2014. Unbinding pathways of VEGFR2 inhibitors revealed by steered molecular dynamics. *J. Chem. Inf. Model.* 54:3124–3136.

100. Di Palma, F., F. Colizzi, and G. Bussi. 2013. Ligand-induced stabilization of the aptamer terminal helix in the add adenine riboswitch. *RNA.* 19:1517–1524.

101. Do, T. N., P. Carloni, …, G. Bussi. 2013. RNA/peptide binding driven by electrostatics-insight from bidirectional pulling simulations. *J. Chem. Theory Comput.* 9:1720–1730.

102. Levintov, L., and H. Vashisth. 2020. Ligand recognition in viral RNA necessitates rare conformational transitions. *J. Phys. Chem. Lett.* 11:5426–5432.

103. Zonta, F., D. Buratto, …, F. Mammano. 2014. Molecular dynamics simulations highlight structural and functional alterations in deafness-related M34T mutation of connexin 26. *Front. Physiol.* 5:85.

104. Nishihara, Y., and A. Kitao. 2015. Gate-controlled proton diffusion and protonation-induced ratchet motion in the stator of the bacterial flagellar motor. *Proc. Natl. Acad. Sci. USA.* 112:7737–7742.

105. Park, S., and K. Schulten. 2004. Calculating potentials of mean force from steered molecular dynamics simulations. *J. Chem. Phys.* 120:5946–5961.

106. Shen, L., J. Shen, …, H. Jiang. 2003. Steered molecular dynamics simulation on the binding of NNRTI to HIV-1 RT. *Biophys. J.* 84:3547–3563.

107. Moore, D. S., C. Brines, …, I. G. Tikhonova. 2018. Steered molecular dynamics simulations reveal critical residues for (un)binding of substrates, inhibitors and a product to the malarial M1 aminopeptidase. *PLoS Comput. Biol.* 14:e1006525.

108. Patel, J. S., A. Berteotti, …, A. Cavalli. 2014. Steered molecular dynamics simulations for studying protein-ligand interaction in cyclin-dependent kinase 5. *J. Chem. Inf. Model.* 54:470–480.

109. Harada, K., S. S. Martin, …, A. D. Frankel. 1997. Molding a peptide into an RNA site by *in vivo* peptide evolution. *Proc. Natl. Acad. Sci. USA.* 94:11887–11892.

110. Jensen, M. Ø., S. Park, …, K. Schulten. 2002. Energetics of glycerol conduction through aquaglyceroporin GlpF. *Proc. Natl. Acad. Sci. USA.* 99:6731–6736.

111. Jarzynski, C. 1997. Nonequilibrium equality for free energy differences. *Phys. Rev. Lett.* 78:2690–2694.

112. Park, S., F. Khalili-Araghi, …, K. Schulten. 2003. Free energy calculation from steered molecular dynamics simulations using Jarzynski's equality. *J. Chem. Phys.* 119:3559–3566.

113. Darby, J. F., A. P. Hopkins, …, M. Fischer. 2019. Water networks can determine the affinity of ligand binding to proteins. *J. Am. Chem. Soc.* 141:15818–15826.

114. Torrie, G. M., and J. P. Valleau. 1977. Nonphysical sampling distributions in Monte Carlo free-energy estimation: umbrella sampling. *J. Comput. Phys.* 23:187–199.

115. Maragliano, L., B. Roux, and E. Vanden-Eijnden. 2014. Comparison between mean forces and swarms-of-trajectories string methods. *J. Chem. Theory Comput.* 10:524–533.

116. Case, D. A., I. Y. Ben-Shalom, …, P. A. Kollman. 2018. AMBER 2018. University of California, San Francisco.

117. Levintov, L. 2021. Molecular simulation studies of dynamics and interactions in nucleic acids. University of New Hampshire, PhD thesis.

118. Towns, J., T. Cockerill, …, N. Wilkins-Diehr. 2014. XSEDE: accelerating scientific discovery. *Comput. Sci. Eng.* 16:62–74.

Biophysical Journal, Volume *120*

Supplemental information

Role of salt-bridging interactions in recognition of viral RNA by arginine-rich peptides

Lev Levintov and Harish Vashisth

Table of Contents

Supplemental Methods.......... pg. S2
Supplemental Results.......... pg. S3
Table S1.......... pg. S4
Table S2.......... pg. S5
Figure S1.......... pg. S6
Figure S2.......... pg. S7
Figure S3.......... pg. S7
Figure S4.......... pg. S8
Figure S5.......... pg. S9
Figure S6.......... pg. S10
Figure S7.......... pg. S11
Figure S8.......... pg. S12
Figure S9.......... pg. S12
Figure S10.......... pg. S13
Figure S11.......... pg. S14
Figure S12.......... pg. S15
Figure S13.......... pg. S16
Figure S14.......... pg. S17
Figure S15.......... pg. S18
Figure S16.......... pg. S19

SUPPLEMENTAL METHODS

Umbrella sampling simulations

We also computed the free-energy profile of peptide dissociation along PW4 using umbrella sampling (US) simulations to compare with the free-energy profile computed using cv-SMD simulations. In US simulations, the reaction coordinate is divided into a series of continuous windows in which a harmonic "umbrella" potential is added in addition to the force-field to bias the system and sample the conformational space limited to a given window. As the initial states for each window, we used the snapshots from the SMD simulation that required the least amount of work for peptide dissociation along PW4.
As a reaction coordinate, we used a distance collective variable which connected the C5 atom of the G47 nucleotide and the center of mass of the peptide residues Gly11 through Ala22. This collective variable was aligned with the vector of the pulling direction in PW4. The reaction coordinate was divided into 1 Å intervals from the initial binding pocket (r = 0 Å) to the unbound state (r = 50 Å). The bias potentials of each US window were set to 12 kcal $mol^{-1}Å^{-2}$ and the width of each window was set to 2 Å. We generated two independent data-sets of 50 windows each, where each window was sampled for 10 ns.
We maintained the temperature and pressure at 310 K and 1 atm using the Langevin thermostat and the Berendsen barostat, respectively. We applied restraints with k = 1000 kcal $mol^{-1}Å^{-2}$ to the phosphorous (P) atoms in the RNA backbone to prevent the overall rotation and translation of the RNA molecule. We also applied restraints to the atoms forming hydrogen bonds in the peptide residues Gly11 through Ala22 to maintain the secondary structure of the peptide during the dissociation. Based on two independent datasets, the free-energy profile was constructed using the weighted histogram analysis method (WHAM) software by the Grossfield lab (Fig. S13).[a]

Swarms-of-trajectories string method

We further used the swarms-of-trajectories string method (STSM) to test the reliability of the lowest free-energy pathway (PW4). The STSM is a path-finding algorithm that can refine the previously identified transition pathway by defining a string with a particular number of nodes or images from the original pathway which connect the initial and the final states. These images are then reparameterized in a high-dimensional space of defined collective variables, whose position is updated after each iteration. At first, a restrained simulation around the center of each image is conducted to generate representative conformations before allowing a small change in this center to occur in a series of free unrestrained simulation for the next iteration. The change in the center of each image is estimated by averaging over the drifts of a number of short unbiased trajectories which start from the conformations generated using the constrained simulations. Thus, the iteration consists of a series of simulations with and without restraints.
As the reaction coordinate, we used a distance collective variable which connected the C5 atom of the G47 nucleotide and the center of mass of the peptide residues Gly11 through Ala22. This collective variable was aligned with the vector of the pulling direction in PW4. As the initial states for 12 images in the string, we used 12 snapshots from the SMD simulation that required the least amount of work to be performed in PW4. We divided the reaction coordinate in 2 Å intervals from r = 0 Å to r = 22 Å. For each image, we conducted 10 MD simulations, each 100 ps long, in all 1000 ps simulation time per image per iteration. In total, we conducted 20 iterations per image. We maintained the temperature and pressure at 310 K and 1 atm using the Langevin thermostat and the Berendsen barostat, respectively. We applied restraints with k = 1000 kcal $mol^{-1}Å^{-2}$ to the phosphorous (P) atoms in the RNA backbone to prevent the overall rotation and translation of the RNA molecule.

PMF of the mutated RSG-1.2 peptide

To test the effect of mutations on the stability of the RSG-1.2 peptide, we conducted additional cv-SMD simulations by mutating each of the three key arginine residues (R8, R14, and R15) to an alanine residue. We energy minimized the system via the steepest descent minimization for 1000 steps. We then briefly equilibrated the volume of the simulation domain via a 500 ps long MD simulation in the NPT ensemble with a 2 fs timestep. We used the coordinates from the end of this MD simulation for subsequent cv-SMD simulations (10 simulations in total) in the NPT

[a] `http://membrane.urmc.rochester.edu/?page_id=126`

ensemble. The parameters and settings for these cv-SMD simulations were identical to the cv-SMD simulations conducted along the reaction coordinate for PW4. The resulting free energy profile is presented in Fig. S16.

SUPPLEMENTAL RESULTS

Effect of restraints and ensemble on the free energy profiles

In our work, we applied rotational restraints to prevent the peptide from rotating while it dissociates, which improved the convergence of the free-energy profile. At first, we conducted 350 cv-SMD simulations along each pathway without the application of rotational restraints. In Fig. S15A, we show the free-energy profiles along PW4 based on 350 cv-SMD simulations without the rotational restraints (red trace) and based on 75 cv-SMD simulations with the rotational restraints (blue trace). When rotational restraints were not applied, the free-energy profile did not converge (Fig. S15A). Additionally, we observed that the free-energy profile of simulations without rotational restraints resulted in a higher free-energy difference in comparison to the free-energy profile of simulations with rotational restraints. Thus, when rotational restraints were applied, the free-energy profile converged faster than without rotational restraints.

We also applied secondary structure restraints to prevent the peptide from unfolding during the cv-SMD simulations. If the secondary structure restraints were not applied, the peptide would unfold due to the pulling force experienced in cv-SMD simulations. Thus, the peptide unfolding would result from the unbinding forces and not from the natural forces of interaction with the RRE RNA or water molecules. We show this unphysical behavior in snapshots from a cv-SMD simulations along PW1 (Fig. S15B).

We further tested the effect of choice of ensemble on the free energy profile along PW4. Specifically, we conducted 30 cv-SMD simulations along PW4 in the NVT ensemble with the same set of parameters and settings as in the cv-SMD simulations with the NPT ensemble except for the ensemble settings. We present the resulting free-energy profile based on the NVT and NPT ensembles in Fig. S15C. We observed that the final free-energy difference between r = 0 Å and r = 50 Å in the NVT ensemble was 95.44 $\pm$ 13.04 kcal/mol which was comparable to the final free-energy difference in the NPT ensemble of 92 $\pm$ 11.32 kcal/mol (Fig. S15C). Thus, the final free-energy differences were similar and the effect of ensemble choice was minimal.

Table S1. Details on all simulation systems. The simulation details on all four pathways (PWs) are presented, including the dimensions of the simulation domain of each system (column labeled *system dimensions*), number of atoms (column labeled *system size*), pulling distance, simulation time of a single run, and the number of runs. The system size is slightly distinct in each pathway due to the initial reorientation of the RNA-peptide complex and resolvation.

PW	system dimensions	system size (atoms)	pulling distance (Å)	time/run (ns)	# runs
1	68 Å × 84 Å × 126 Å	63919	80	13	75
2	65 Å × 79 Å × 133 Å	61171	80	13	75
3	64 Å × 84 Å × 129 Å	61618	80	13	75
4	70 Å × 83 Å × 130 Å	66511	80	13	75

Table S2. Details on salt bridging interactions. The details on the atom of the amino acid (*Peptide*) and the atom of the nucleotide (*RNA*) that participate in salt bridging interactions are presented for each pathway (PW).

PW	Peptide	RNA
1	NH2/R15	O1P/U45
2	NH1/R8	O1P/G48
	NH2/R14	O2P/A68
	NH2/R15	O2P/U45
	NH1/R15	O1P/C44
3	NH2/R8	O2P/A68
	NH2/R14	O2P/A68
	NH2/R15	O2P/G42
4	NH1/R8	O1P/U72
	NH2/R14	O2P/A68
	NH1/R14	O1P/C69
	NH2/R15	O1P/C44

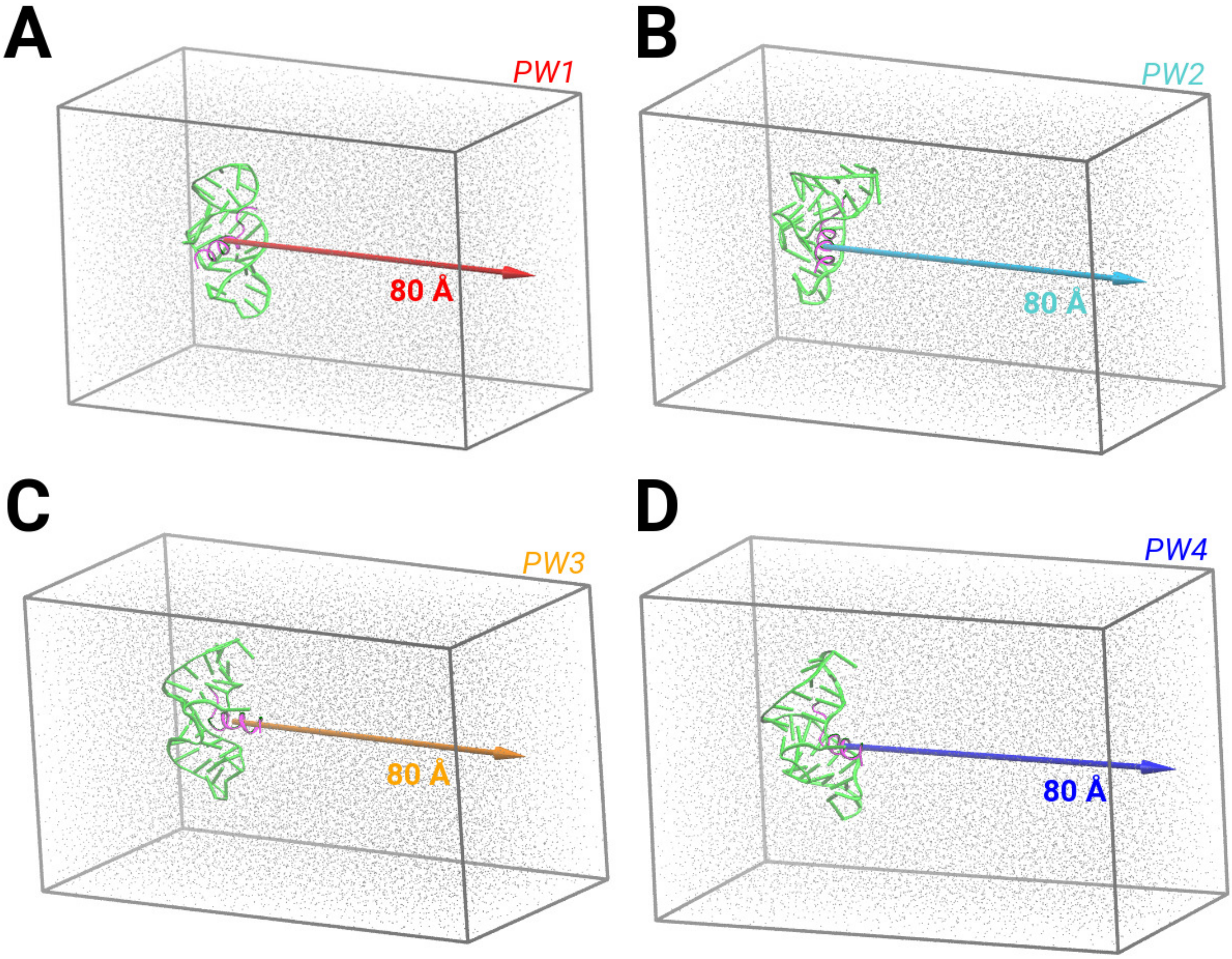

Figure S1. System setup: Shown are the side-views of the simulation domains along (A) pathway 1 (PW1), (B) pathway 2 (PW2), (C) pathway 3 (PW3), and (D) pathway 4 (PW4). In each snapshot, RNA is represented as a green cartoon; peptide as a purple cartoon; water molecules as gray points; and the bounding box in gray. The arrow in each panel indicates the reaction coordinate for each pathway that was used to conduct non-equilibrium cv-SMD simulations.

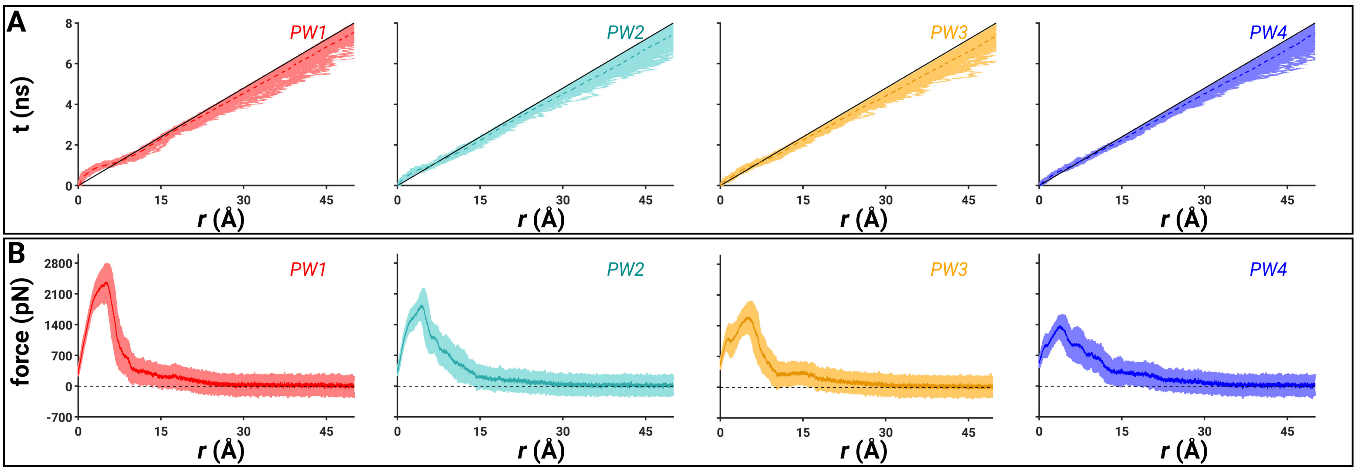

Figure S2. The reaction coordinates (*r*) and the unbinding force profiles (A) The center-of-mass (COM) trajectories of the peptide are shown in unique colors for PW1 (red), PW2 (cyan), PW3 (orange), and PW4(blue). The black solid lines represent the actual ***r***; the dark dotted lines represent the average trace across 75 trajectories for the corresponding PW; and the lighter shaded lines represent all SMD trajectories for the corresponding PW. (B) The unbinding force profiles are shown in unique colors for PW1 (red), PW2 (cyan), PW3 (orange), and PW4 (blue) with the average force traces (darker solid lines) and standard deviations (lighter shades).

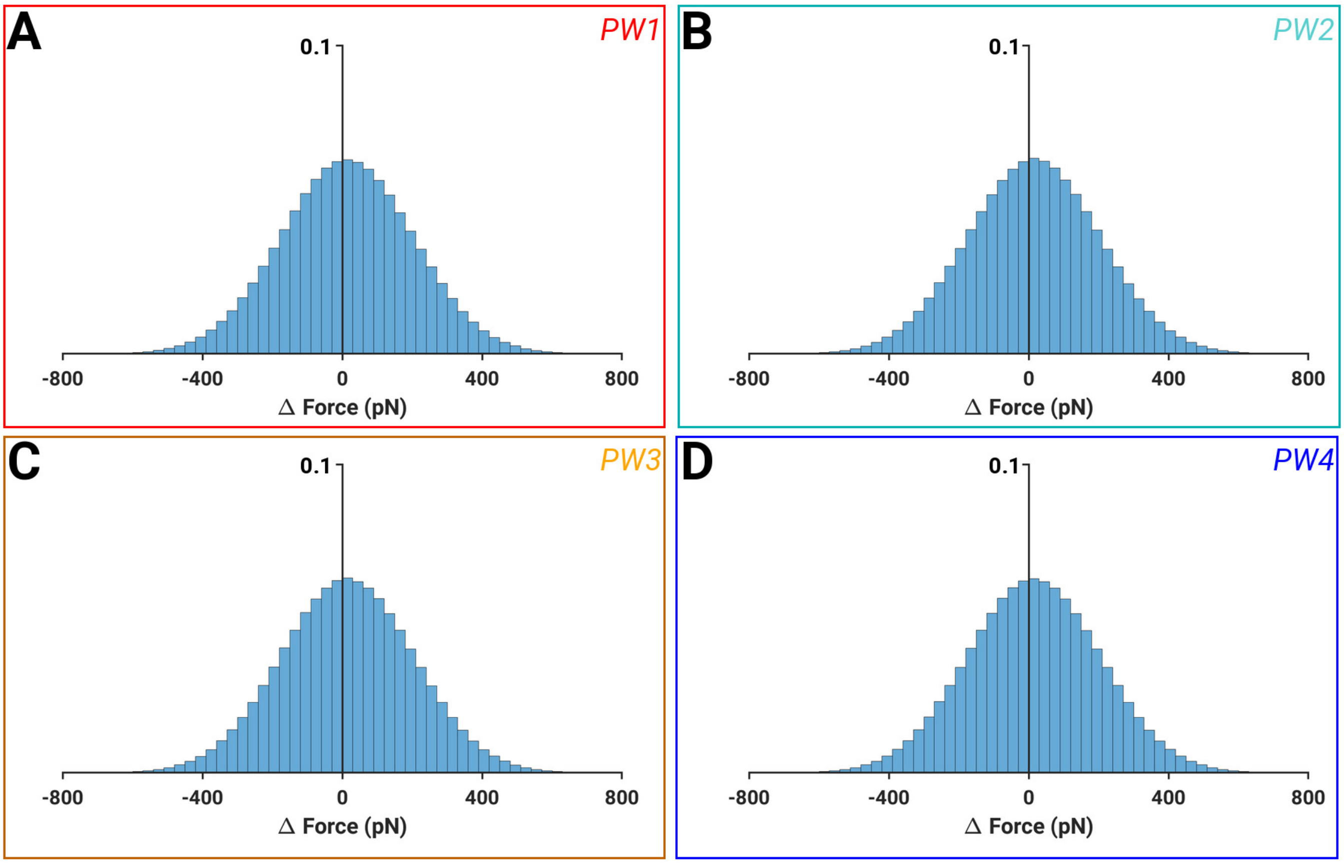

Figure S3. Force convergence data: Shown are the distributions of ΔForce values, defined as a difference between zero and the actual force value after the average force profile converged to zero for (A) PW1, (B) PW2, (C) PW3, and (D) PW4. The ΔForce values were measured after a distance of 40 Å along the reaction coordinate. See also Fig. S2B.

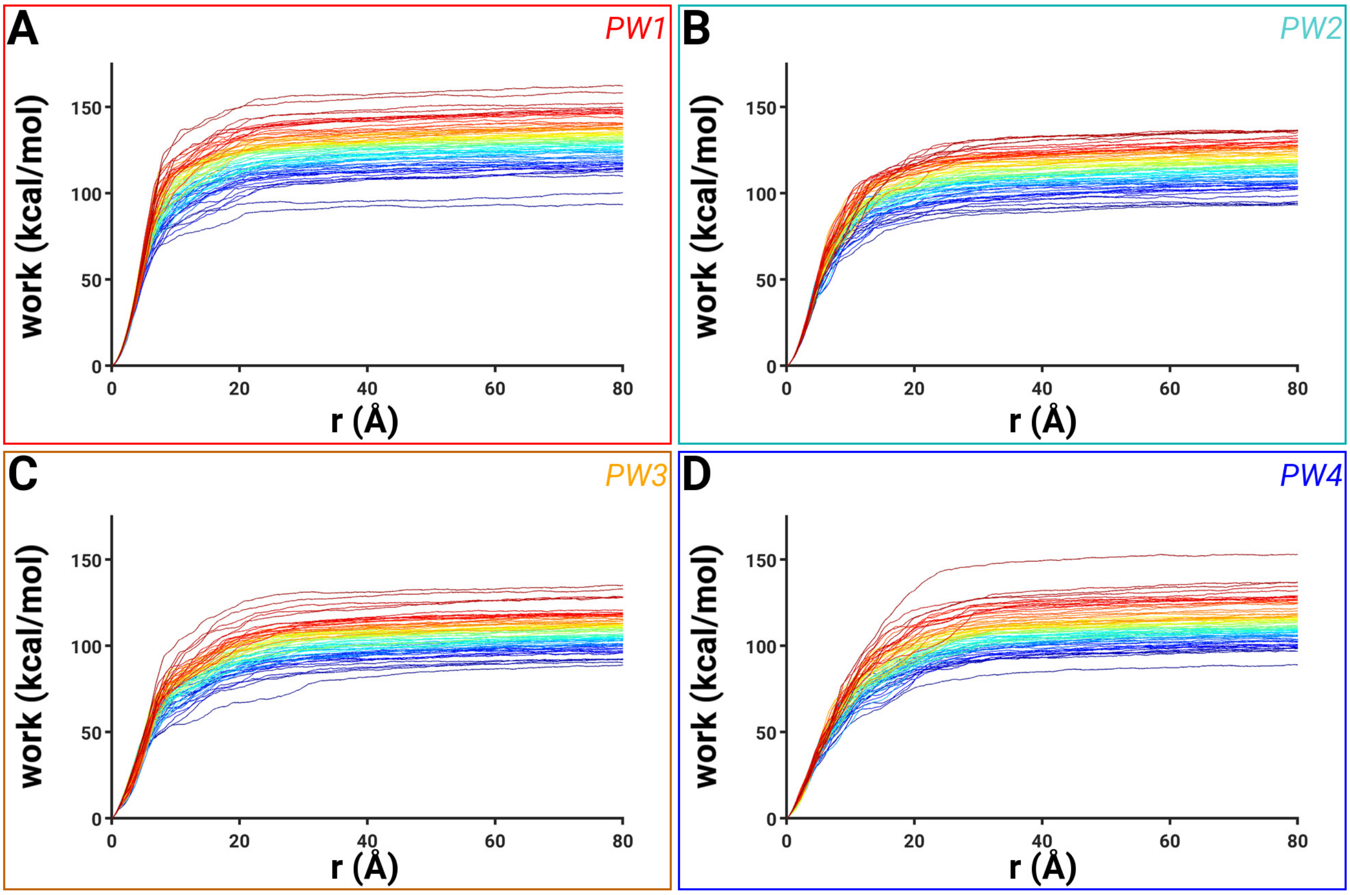

Figure S4. Non-equilibrium work profiles: The non-equilibrium work values obtained from 75 independent cv-SMD simulations for (A) PW1, (B) PW2, (C) PW3, and (D) PW4. The lower work values are indicated in blue traces and the higher work values in red traces.

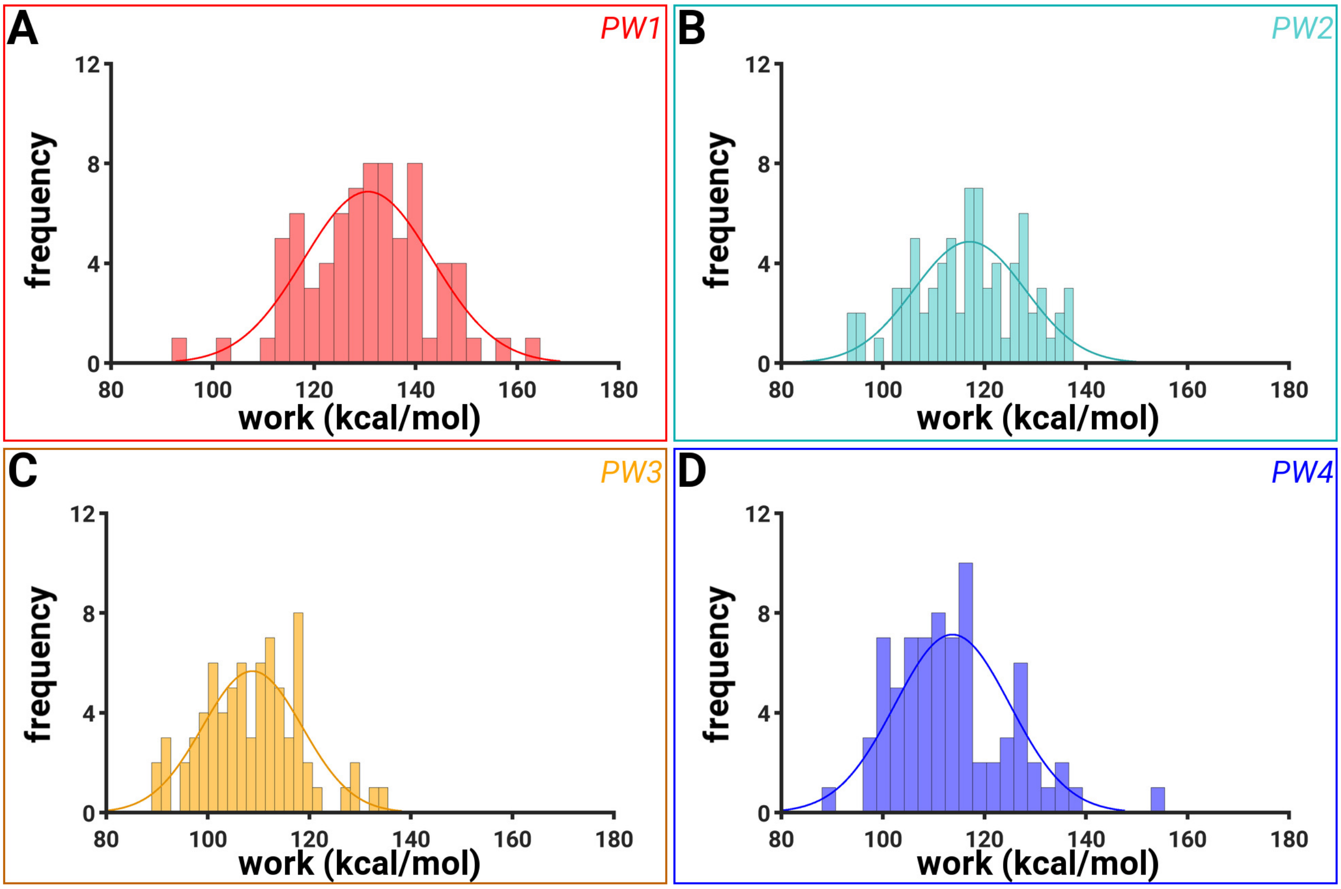

Figure S5. Distributions of the final work values: The histograms of all final work values during cv-SMD simulations are shown with a best-fit distribution line for (A) PW1, (B) PW2, (C) PW3, and (D) PW4.

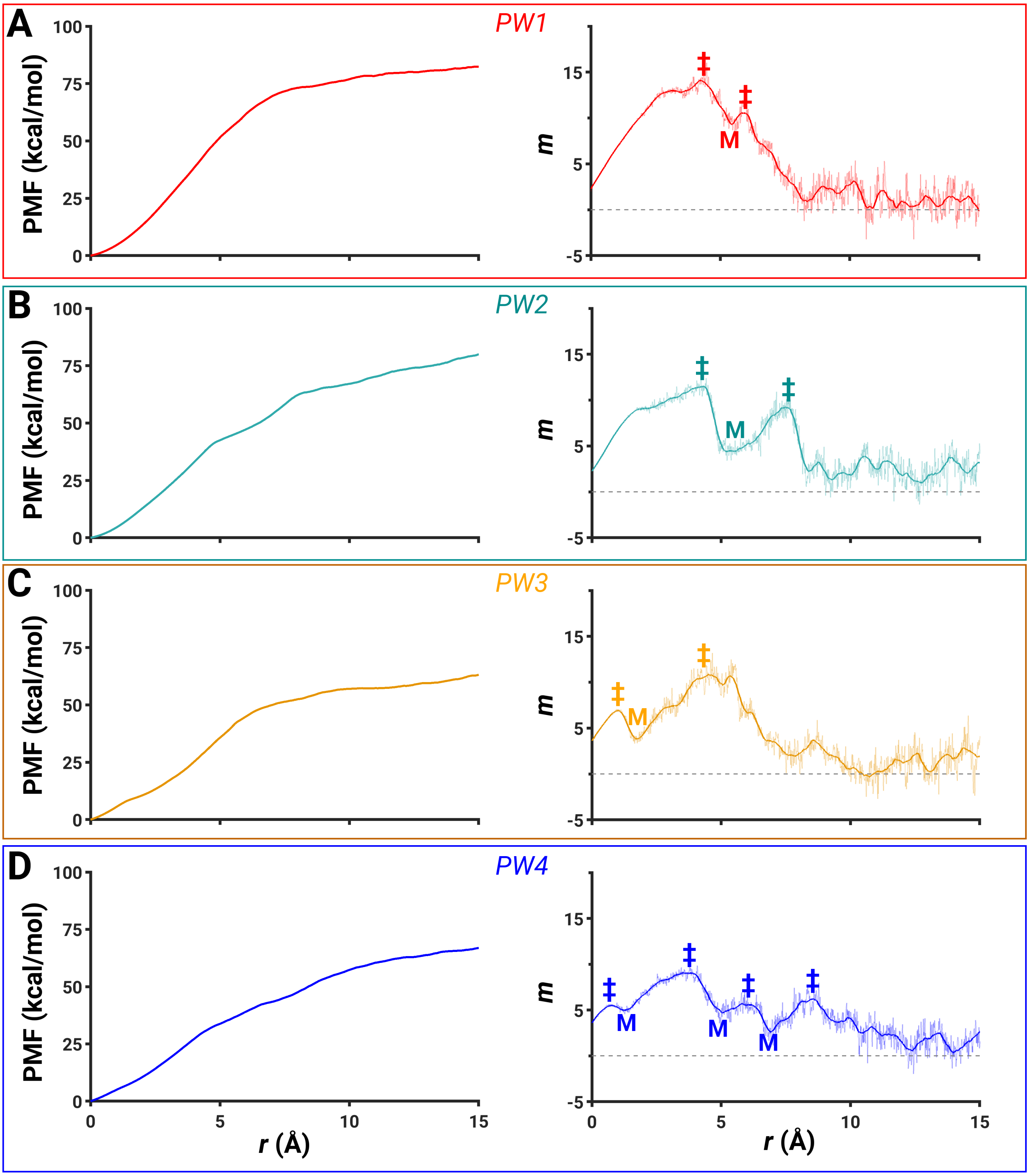

Figure S6. The free-energy and the corresponding first-order derivative profiles: A zoomed view of each free-energy profile (*left*) and the corresponding first-order derivative profile (*m*; *right*) computed every 100 points for r values between 0 Å and 15 Å are shown for (A) PW1, (B) PW2, (C) PW3, and (D) PW4. The fluctuations of the first-order derivatives are shown in light shaded colors. The free-energy barriers (indicated by ‡) and metastable states (indicated by M) are also shown and labeled.

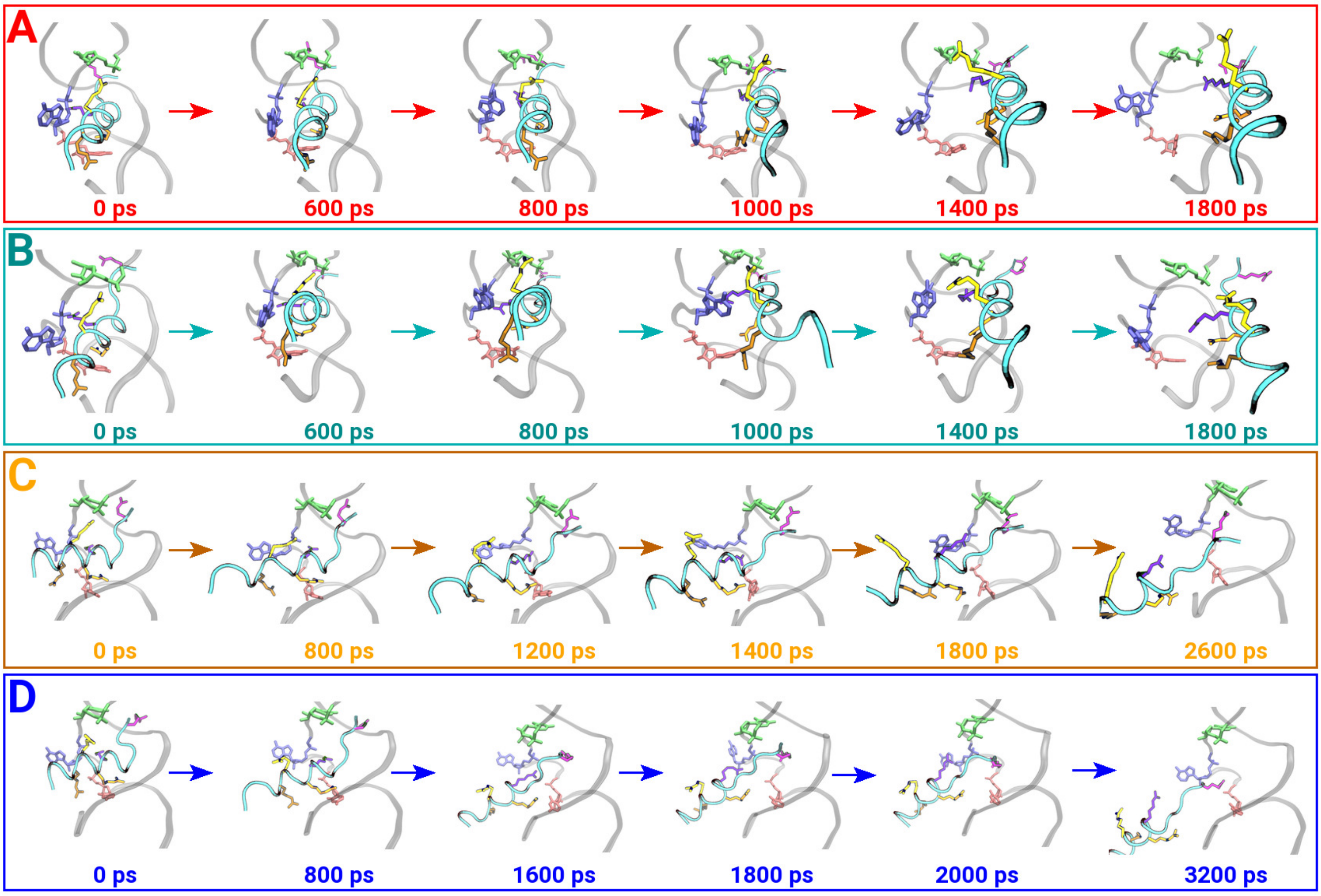

Figure S7. Dissociation pathways: Shown are the snapshots of the peptide (cyan tube with key amino acids highlighted) dissociating from the RRE RNA (gray cartoon) from the least work cv-SMD simulation for (A) PW1 (red), (B) PW2 (cyan), (C) PW3 (orange), and (D) PW4 (blue).

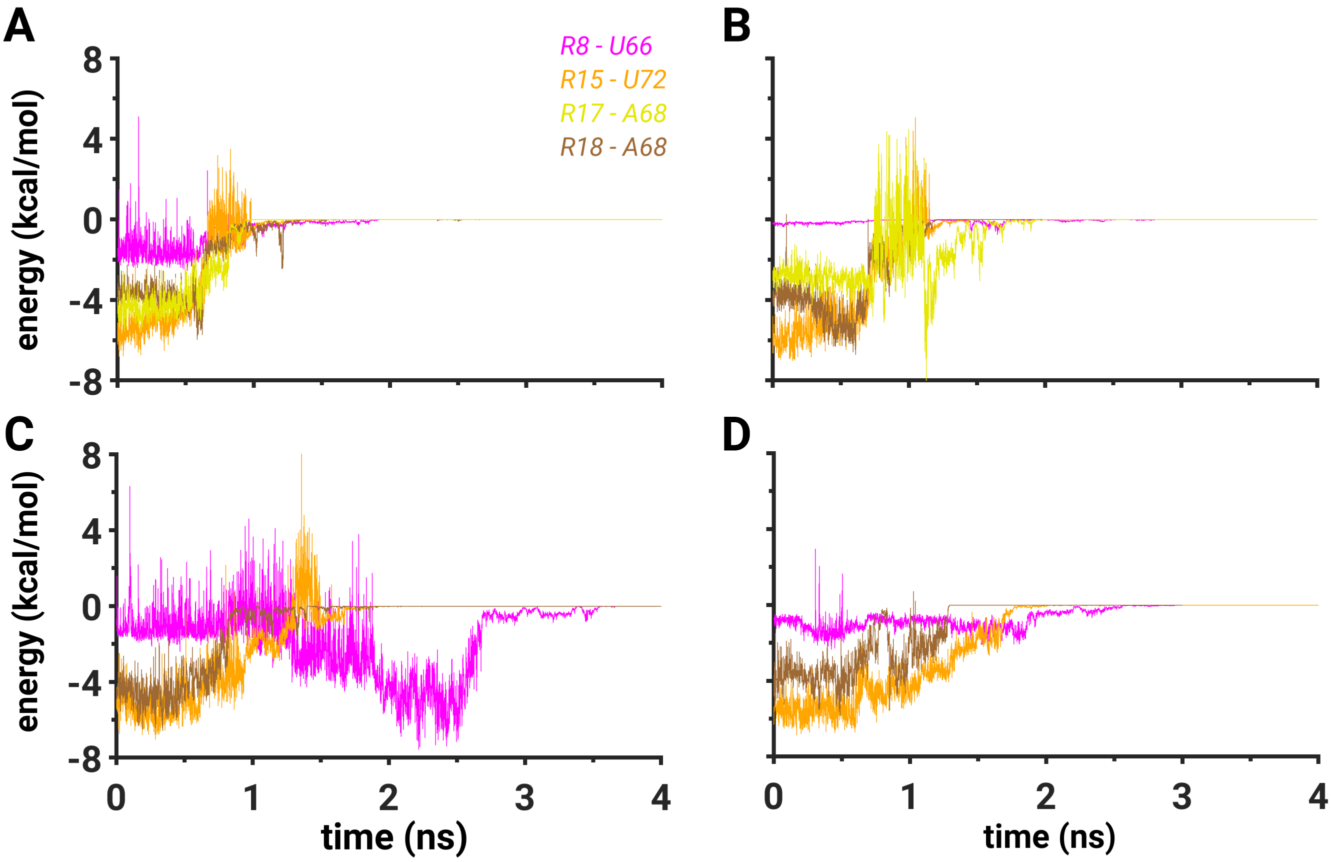

Figure S8. Van der Waals interaction energies: Shown are the time traces of the van der Waals interaction energy computed between the following amino acid - nucleotide pairs: the Arg8 (R8) amino acid and the U66 nucleotide (purple); the Arg15 (R15) amino acid and the U72 nucleotide (orange); the Arg17 (R17) amino acid and the A68 nucleotide (yellow); the Arg18 (R18) amino acid and the A68 nucleotide (brown). The energies were computed from the simulation with the least work in (A) PW1, (B) PW2, (C) PW3, and (D) PW4. Data after 4 ns are truncated due to the convergence to zero of each van der Waals energy trace.

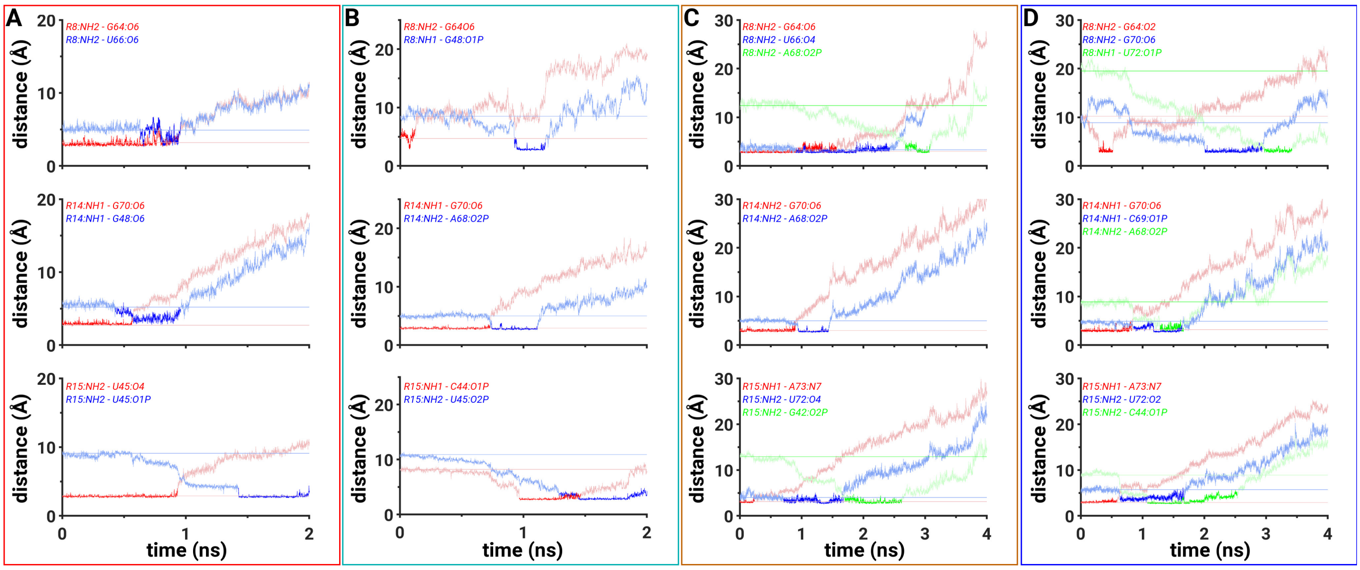

Figure S9. Key interatomic distance traces along each pathway: Shown are the traces of distances for each pathway (panel A, PW1; panel B, PW2; panel C, PW3; and panel D, PW4) corresponding to which data are presented in Figs. 3-6, where the y-axis scale is limited to 15 Å (Figs. 3 and 4) or 20 Å (Figs. 5 and 6).

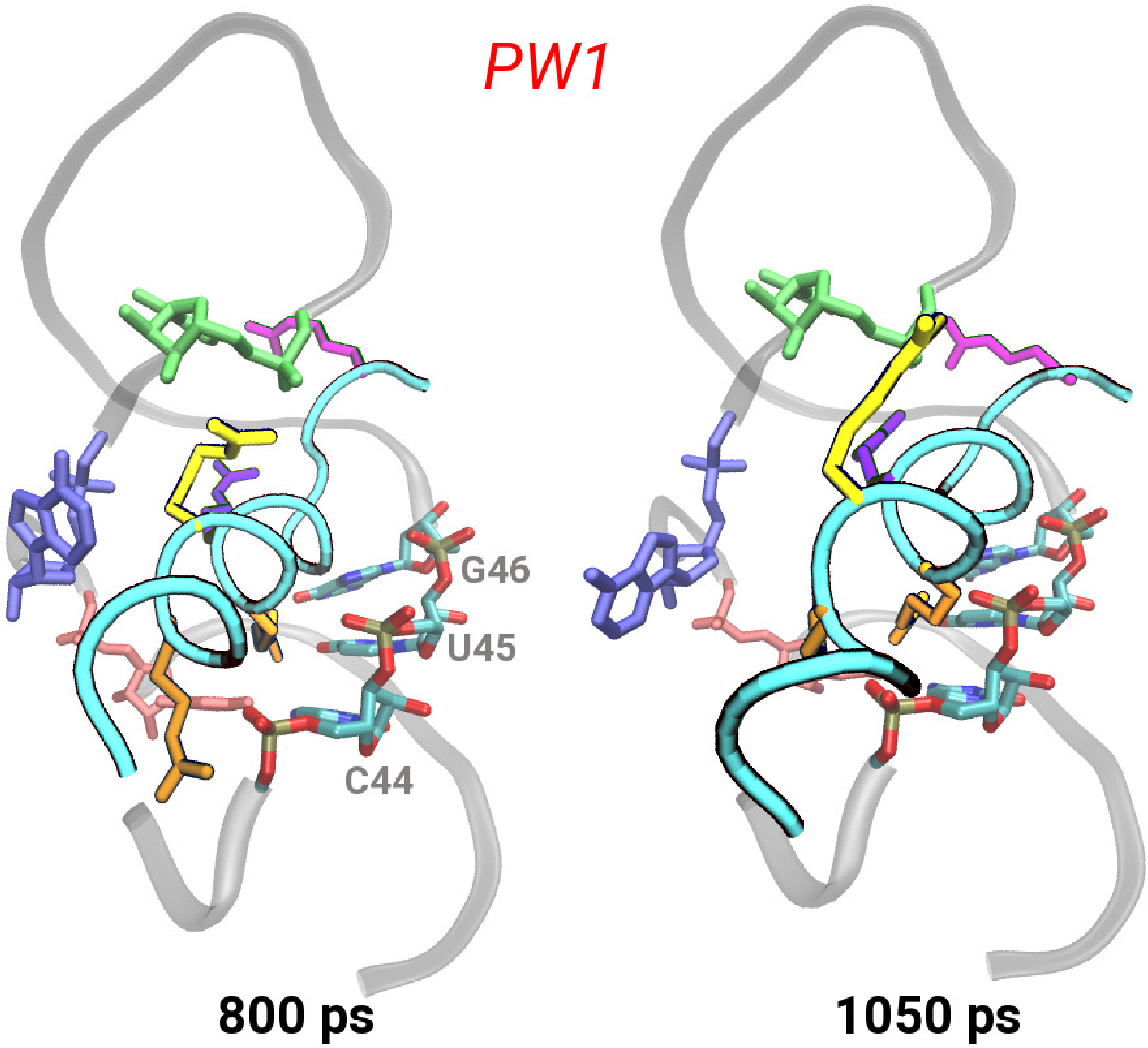

Figure S10. Snapshots from PW1: Shown are the snapshots of the peptide (cyan tube with key amino acids highlighted) dissociating from the RRE RNA (gray cartoon) from the lowest work simulation of PW1. Three nucleotides (U44, U45, and G46) which interact with the peptide through the atoms in the backbone are each shown in a stick representation. The color scheme is the same as in Fig. 1B.

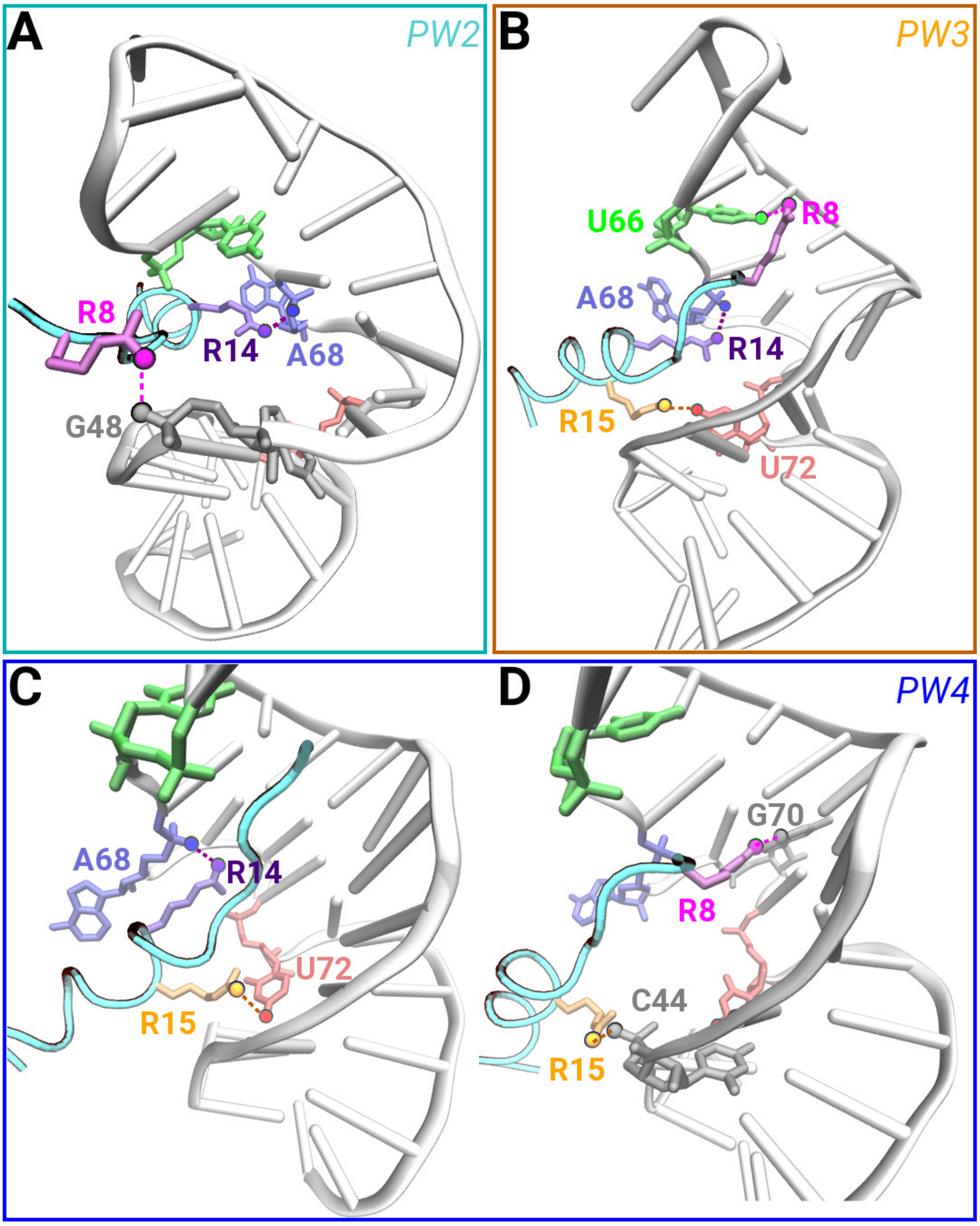

Figure S11. Networks of salt bridging and hydrogen bonding interactions: Shown are the snapshots of the peptide (cyan tube with key amino acids highlighted) dissociating from the RRE RNA (white cartoon) and forming a network of salt bridging and hydrogen bonding interactions from the least work cv-SMD simulations in (A) PW2, (B) PW3, and (C-D) PW4. Each amino acid, each nucleotide or an atom that participate in hydrogen bonding or salt bridging interactions (marked by a dashed line), are uniquely colored. The color scheme is the same as in Fig. 1B.

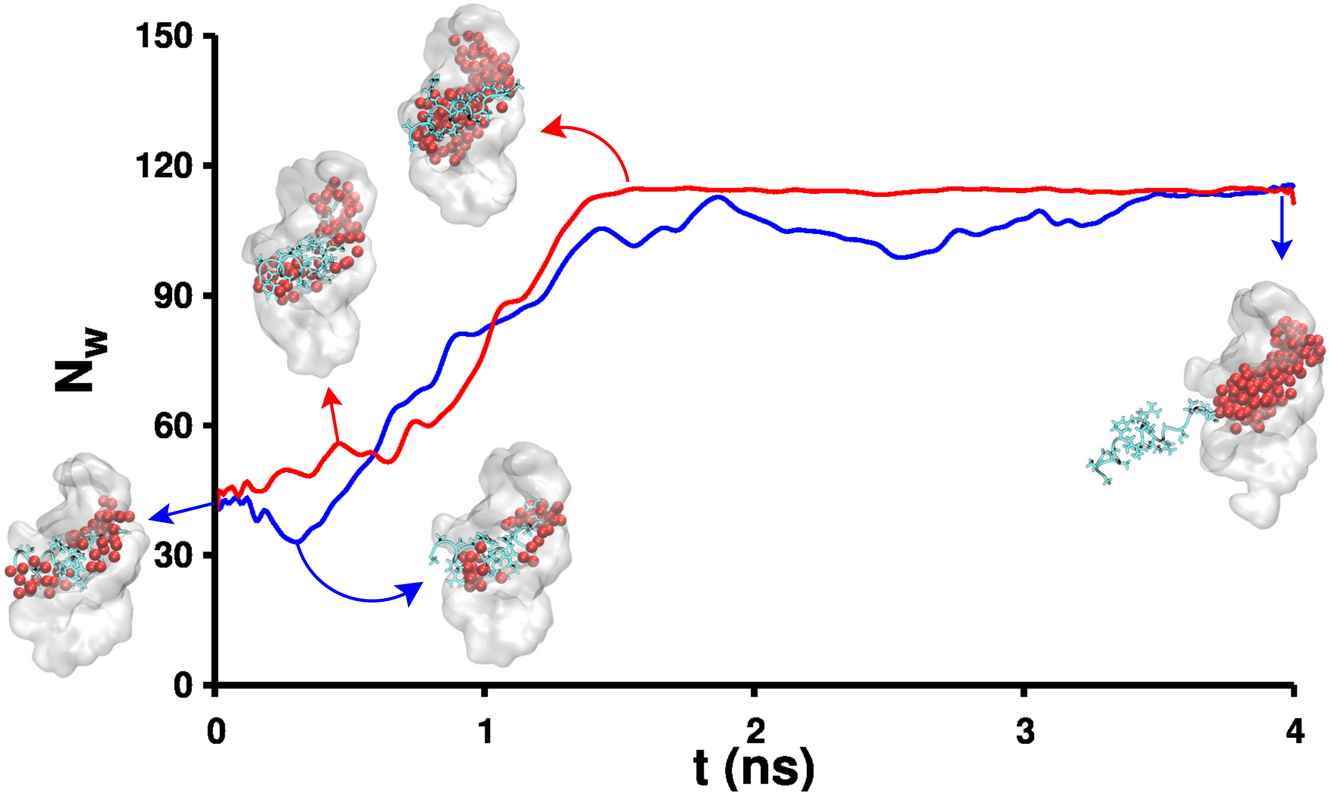

Figure S12. Solvation mechanism of the binding pocket: Shown are the time traces of the number of water molecules (N_w) solvating the binding pocket of the RRE RNA along PW1 (red) and PW4 (blue). The side-view snapshots are also shown of the solvation of the binding pocket at various time-points along each pathway. The RRE RNA is represented as a white surface, the peptide as a cyan cartoon, and the oxygen atoms of the water molecules as red spheres.

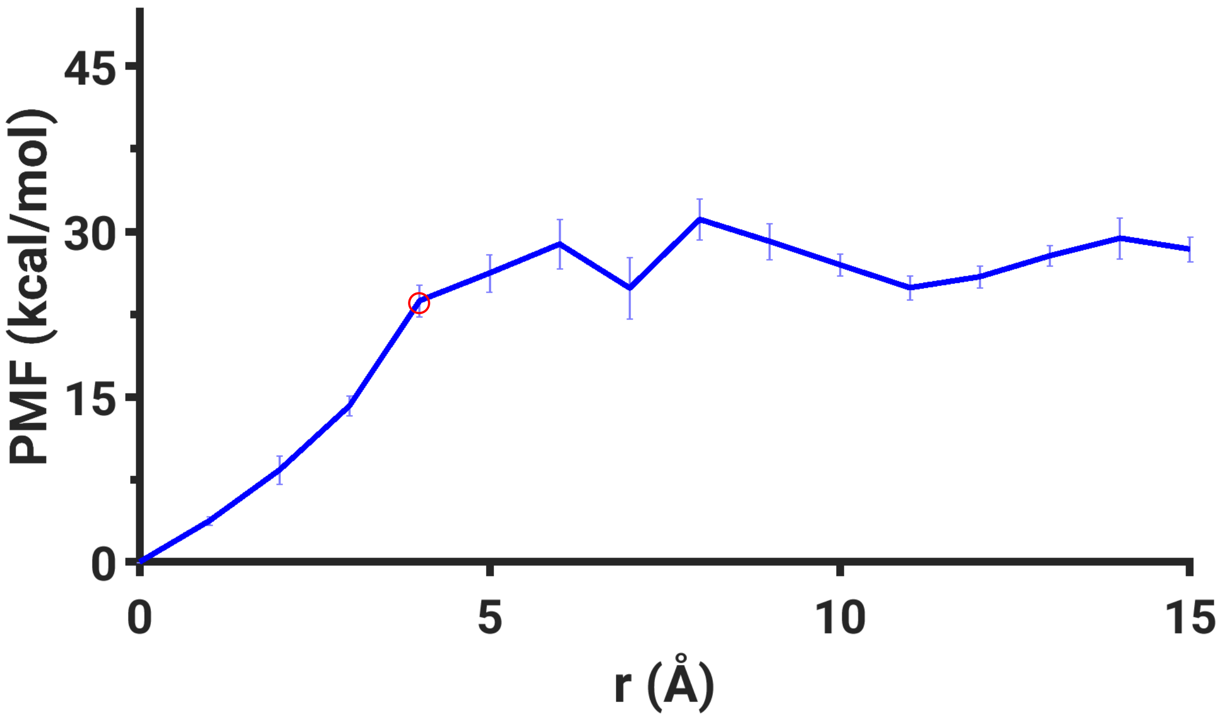

Figure S13. Umbrella sampling calculations for PW4. The free-energy profile along PW4 based on umbrella sampling simulations is shown along with the free-energy barrier (red circle) and the error bars (blue). The barrier calculated from the umbrella sampling calculations was 23.72±1.46 kcal/mol at ∼4 Å which is comparable to the free energy barrier of 24.46±2.08 kcal/mol at ∼3.8 Å that we have identified using cv-SMD simulations for PW4.

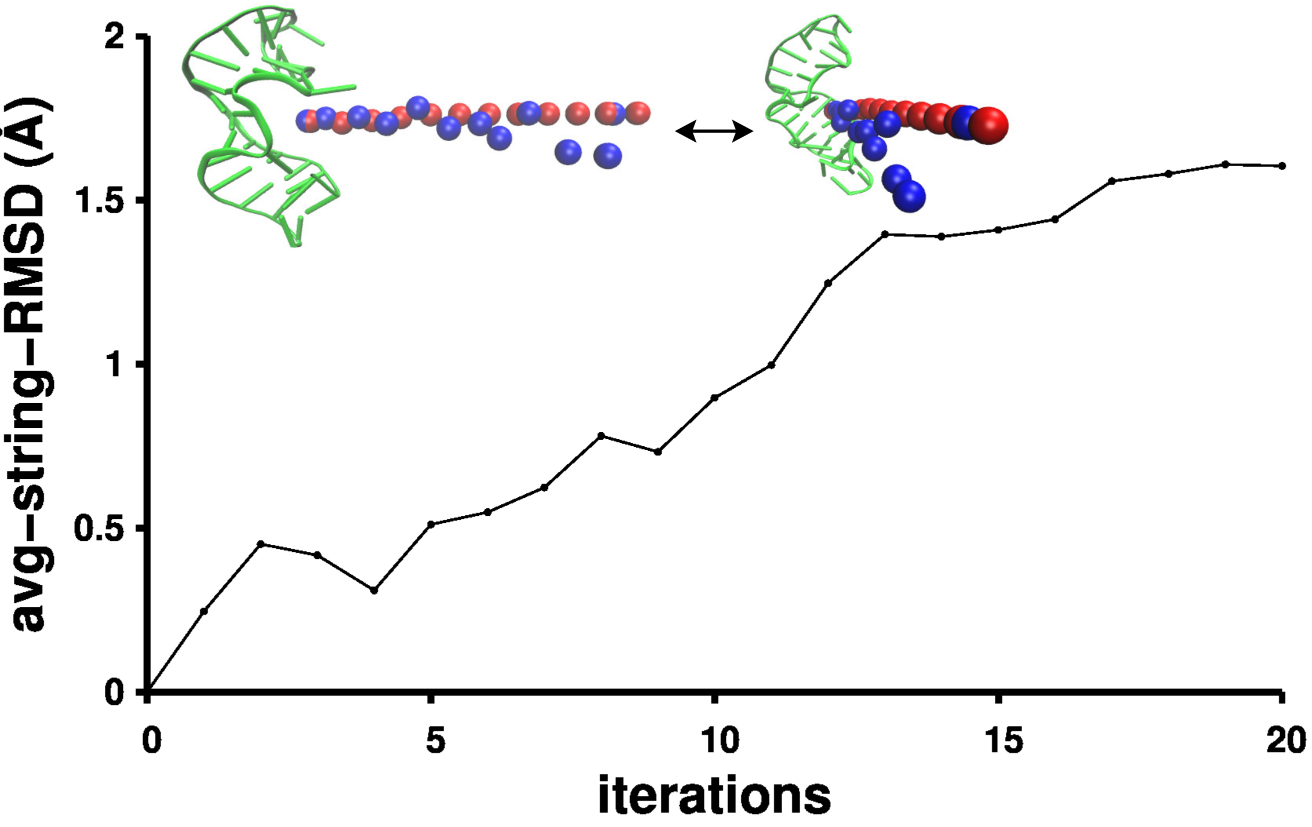

Figure S14. Convergence of the string. The average RMSD of the entire string for each iteration is shown for the application of the strong method to PW4. (*inset*) Shown are two side-view snapshots of the RNA (green cartoon) with the initial positions of the images on the string (red spheres) and the positions of the images at the final iteration (blue spheres). The cv-SMD pathway and the converged pathway from the string method only differ marginally in the bulk solvent.

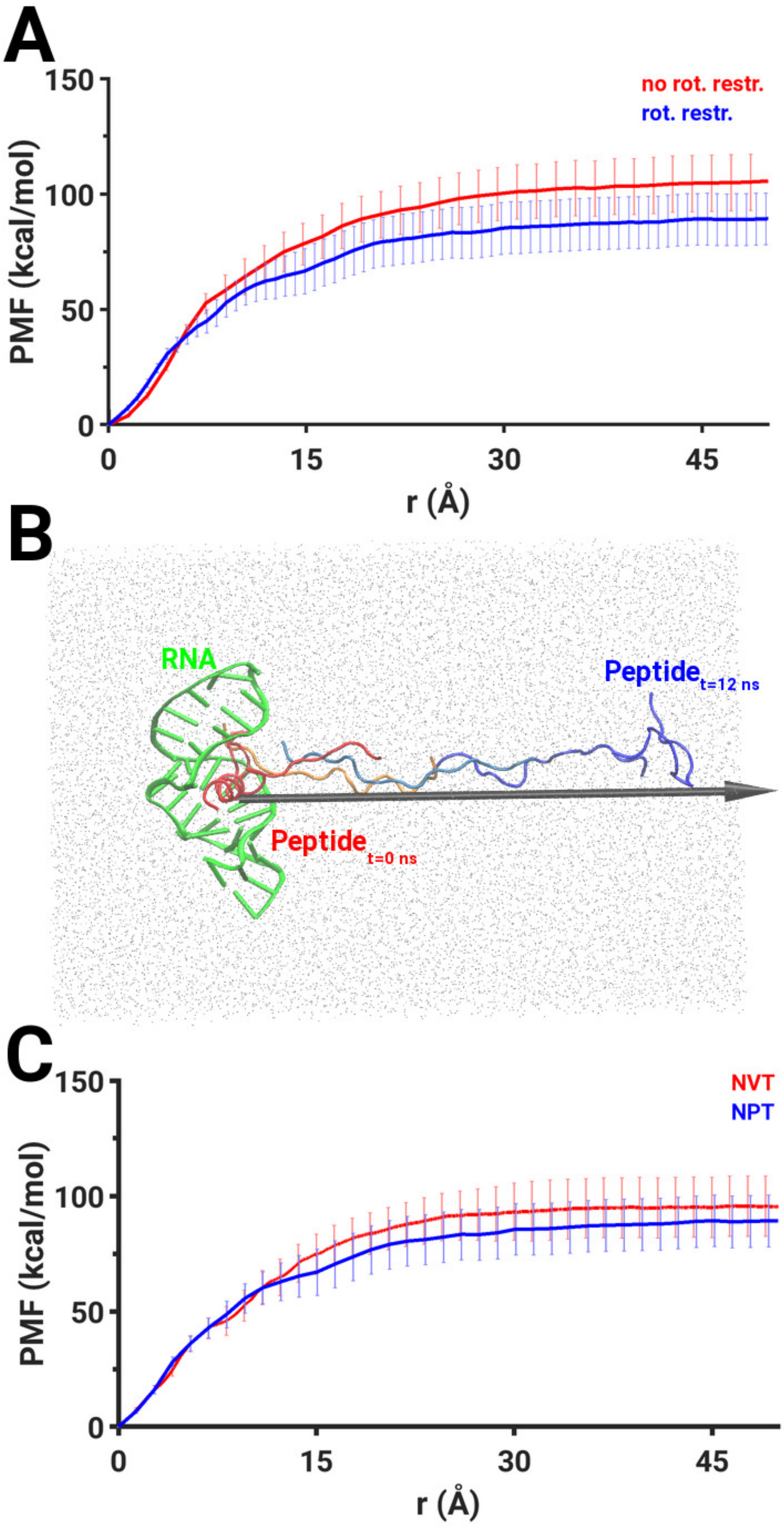

Figure S15. Effect of restraints and ensemble on PMF: (A) The free-energy profiles along PW4 are shown for a system without rotational restraints (red) and with rotational restraints (blue). (B) Shown are the side-view snapshots of dissociation of the peptide along PW1 in the absence of secondary structure restraints. The RNA is represented as a green cartoon and the peptide is shown in a cartoon representation at various time-points during the simulation (red, beginning; blue, end); water molecules are shown as gray points. The black arrow indicates the cv-SMD reaction coordinate. (C) The free-energy profiles along PW4 based on simulations conducted in the NVT (red) and NPT (blue) ensembles are shown.

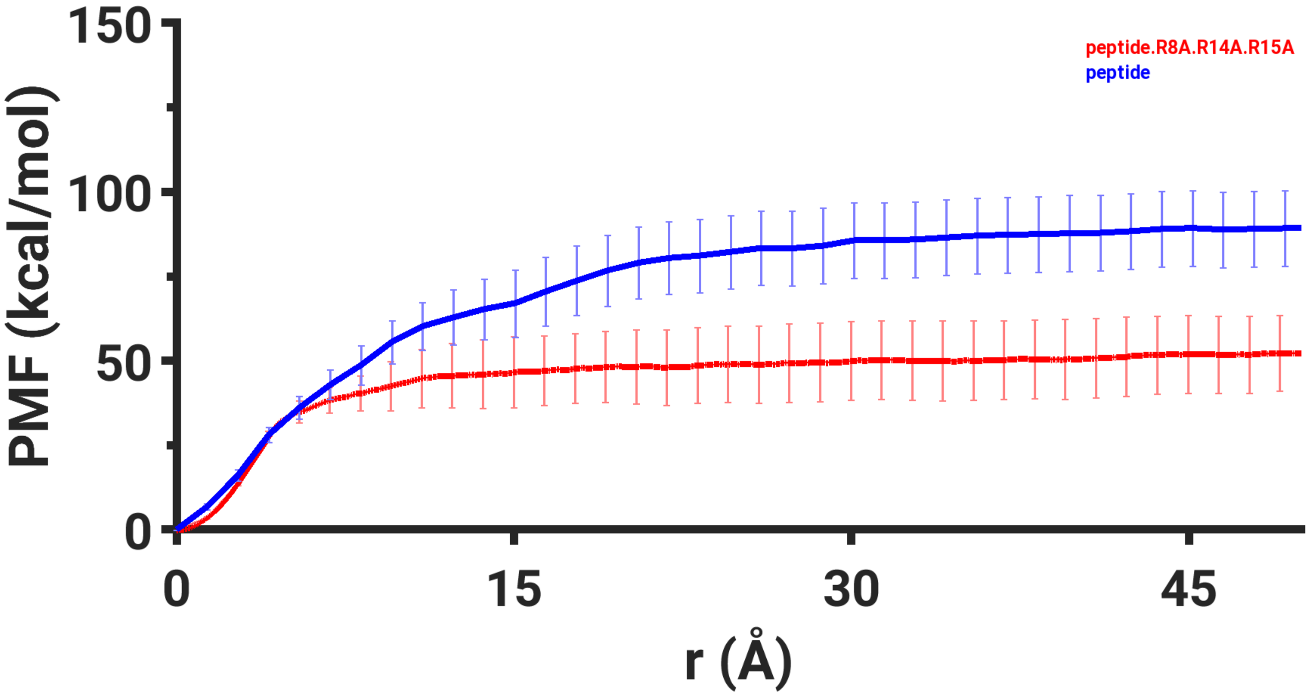

Figure S16. The free-energy profile of the mutated peptide: A comparison of the free-energy profiles is shown along PW4 for the RSG-1.2 peptide (blue trace) and its mutant peptide (red trace) with triple alanine mutations in three key arginine residue (R8, R14, and R15).
